# Synergistic Effects Between Metal Nanoparticles and Commercial Antimicrobial Agents: A Review

**DOI:** 10.1021/acsanm.1c03891

**Published:** 2022-03-04

**Authors:** Ana Isabel Ribeiro, Alice Maria Dias, Andrea Zille

**Affiliations:** † 2C2T - Centre for Textile Science and Technology, Department of Textile Engineering, University of Minho, Campus de Azurém, 4800-058 Guimarães, Portugal; ‡ Centre of Chemistry, Department of Chemistry, University of Minho, Campus de Gualtar, 4710-057 Braga, Portugal

**Keywords:** antimicrobial agents, metal nanoparticles, antibiotics, antifungals, antivirus, synergism

## Abstract

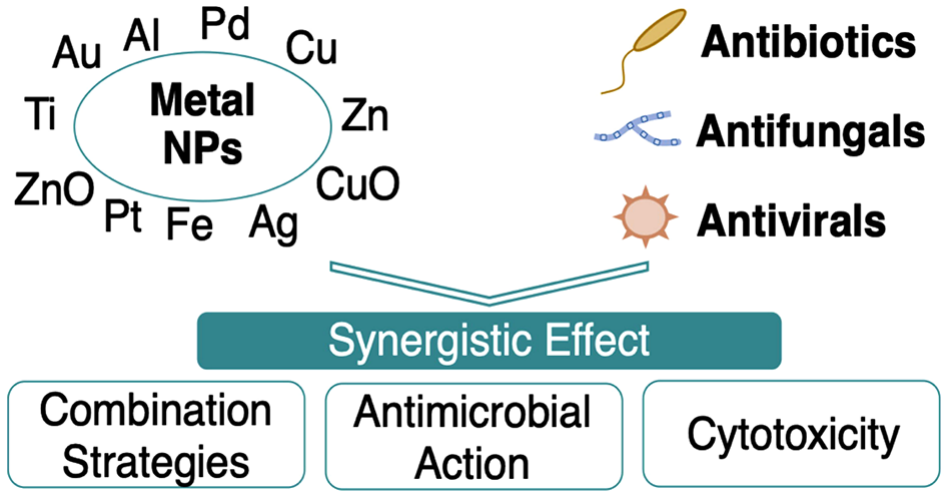

Nanotechnology has expanded into a broad range of clinical applications. In particular, metal nanoparticles (MNPs) display unique antimicrobial properties, a fundamental function of novel medical devices. The combination of MNPs with commercial antimicrobial drugs (e.g., antibiotics, antifungals, and antivirals) may offer several opportunities to overcome some disadvantages of their individual use and enhance effectiveness. MNP conjugates display multiple advantages. As drug delivery systems, the conjugates can extend the circulation of the drugs in the body, facilitate intercellular targeting, improve drug stabilization, and possess superior delivery. Concomitantly, they reduce the required drug dose, minimize toxicity, and broaden the antimicrobial spectrum. In this work, the common strategies to combine MNPs with clinically used antimicrobial agents are underscored. Furthermore, a comprehensive survey about synergistic antimicrobial effects, the mechanism of action, and cytotoxicity is depicted.

## 1 Introduction

The emergence of infectious diseases due to new pathogens and multidrug-resistant (MDR) strains has been a global health threat over the past decades.^1^ A wide range of microbes survive and thrive on living and nonliving surfaces contributing to the development of infectious diseases outbreaks, high levels of healthcare-associated infections, and an increase of MDR pathogens. Consequently, significant health and financial costs occur due to the slower patient treatments, increasing hospitalization times, the disruption of daily activities, discomfort, or even death.^2,3^ Despite promising studies in the development of novel antimicrobial drugs, this field has not been able to keep up with the rapid increase of infections caused by MDR pathogens.^4−6^ It is estimated that antibiotic resistance is causing 700 000 deaths annually worldwide. This number is expected to rise to more than 10 million deaths per year by 2050.^7^ In addition, the effectiveness of conventional antimicrobial drugs is rapidly declining due to mass overconsumption and imprudent dosage. Governments were forced to launch propaganda to inform the mass population of adequate antibiotic consumption.^8^ MDR pathogens pose a particularly grievous threat to human health and even more so with the increasing number of immune-compromised individuals, aging, transplant complications, and stress.^9,10^ In addition, the global pandemic of COVID-19 caused by severe acute respiratory syndrome coronavirus 2 (SARS-CoV-2) intensified the problem of MDR pathogens and the demand for more effective antimicrobial agents. Similarly to other viral infections, severely ill patients are at increased risk of secondary bacterial or fungal infections that can be fatal.^11^ The existing therapeutics are target selective with specific mechanisms of action. Different drugs are combined to provide additional mechanisms of action and broad-spectrum activity. This approach commonly increases the dosage and the adverse side effects.^12^ Thus, the World Health Organization (WHO) has launched an action plan to foment the discovery of effective and safe antimicrobial agents with multiple mechanisms of action.^13^ Moreover, strategies to decrease the risk of microorganism colonization are taken into account to develop new materials that can kill or inhibit microbial growth and adhesion onto surfaces.^14^

Nanotechnology is changing the way healthcare solutions are developed and provided, offering innovative routes to address the progress in antimicrobial therapy, drug delivery, and the development of advanced materials.^15,16^ Metal nanoparticles (MNPs) have been widely applied and studied due to their unique properties: their small size and high surface-volume ratio, ability to act at the cellular level, improved solubility, surface adaptability, and multifunctionality.^17−20^ Despite their exceptional properties and wide range of applications, nanoparticles pose a risk of adverse health effects in humans. *In vitro* and *in vivo* studies have shown that MNPs can penetrate the cells, leading to oxidative stress, inflammation, DNA damage, and organ toxicity and limiting their application.^21^

Few MNPs have been approved for clinical use by the U.S. Food and Drug Administration (FDA) and European Medicines Agency (EMEA), and very few are under clinical trials. The complexity of nanotechnology requires regulatory frameworks related to the inherent risks of nanoparticles (toxicity), effects of exposure, and administration routes. The approved drugs are mainly used for anticancer therapy, iron-replacement therapy, antimicrobial agents, and bone graft substitutes.^22−26^ It is imperative to study the pharmacokinetics of MNP drugs using appropriate models to improve the translatability of MNPs to clinical practice. The MNPs should reach the target site undamaged with high selectivity and reduced accumulation in nontargeted cells, tissues, and organs. Optimal therapeutic benefits can be obtained by functionalizing the nanoparticles with appropriate ligands or combining them with other drugs.^27−30^

Synergistic approaches combine two or more substances together to result in superior efficacy compared to that of any of the individual substances. The conjugation of MNPs with other antimicrobial compounds may enhance their effectiveness. New approaches in the fight against pathogens may be explored, including the revival of old antibiotics, to overcome the current drug resistance emergency.^31,32^ These NP conjugates may exhibit several advantages, such as (i) having multiple targets and mechanisms of action; (ii) suppressing the emergence of resistant pathogens; (iii) improving self-assembly into nanostructures for delivery systems; (iv) facilitating intracellular targeting; (v) prolonging the circulation and stabilization of drugs in the body’s systems; (vi) decreasing the individual dosages that consequently minimize host toxicity; (vii) increasing the spectrum of antimicrobial coverage during therapies.^12,33^ The drug combination is a common strategy in clinical practice, and its therapeutic success has been attained for acquired immunodeficiency syndrome (AIDS), cancer, cardiovascular disease, and microbial infections.^34^

**Chart 1:**
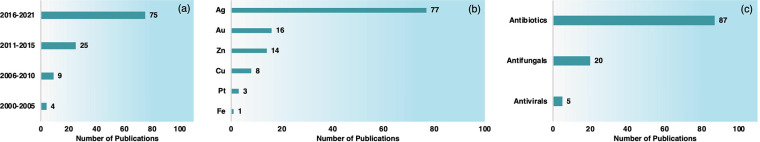
Number of Publications (a) Per Year, (b) Per Type of MNPs, and (c) Per Conjugated Drugs from 2000 until December 2021 in Google Scholar, Web of Science, PubMed, Scopus, and Science Directa

The synergistic effects between MNPs and commercial antimicrobial drugs have been studied for several years. Nevertheless, a relevant increasing number of publications have occurred in the last five years. Most of the synergistic studies focus on silver nanoparticles (AgNPs). However, other MNPs were also reported, such as gold (Au), copper (Cu), copper oxide (CuO), copper sulfide (CuS), iron (Fe), iron oxide (Fe_3_O_4_/Fe_2_O_3_), zinc, zinc oxide (ZnO), and platinum (Pt). MNPs have been combined with several antibiotic, antifungal, and antiviral agents (Chart 1). However, a high number of compounds and MNPs remain unexplored.

a The survey was conducted with a combination of keywords using particular terms related to MNPs, the combining agent, and antimicrobial properties.

This Review focuses on research works conjugating MNPs and commercial antimicrobial drugs such as antibiotics, antifungals, and antivirals to obtain novel antimicrobial formulations. Therefore, conjugation of MNPs with other antimicrobial agents (e.g., disinfectants, antimicrobial peptides, novel organic molecules, essential oils) was not considered. The experimental methodologies to obtain the conjugates are described. The conjugates’ antimicrobial efficacy, mechanism of action, and cytotoxicity are also depicted. Hence, this work envisages contributing to new advances on this topic and promoting the transfer of this knowledge and applications to clinical practice.

## 2 Metal Nanoparticles as Antimicrobial Agents

MNPs’ research has increased due to their improved properties compared to bulk materials. They have allowed the development of novel drugs and materials by tailoring their size, morphology, distribution, and surface charge properties. However, MNP toxicity to humans and the environment has been broadly reported. The main properties of MNPs responsible for their toxicological effects have been attributed to (i) size (NPs below 10 nm usually display high antimicrobial activity but also high cytotoxicity due to their rapid diffusion into human cells and their ability to cross the blood–brain barrier (<200 nm));^35−37^ (ii) agglomeration, which contributes to the sedimentation process and reduces the diffusion of NPs, resulting in higher effective doses;^38^ (iii) surface charge (the charge of NPs presents an essential role in regulating the protein binding to NPs, cellular uptake, oxidative stress, autophagy, inflammation, and apoptosis; charged NPs were shown to be more cytotoxic than neutral forms, and positively charged NPs were more cytotoxic than negative variants of a similar size).^39,40^ Currently, MNPs can be designed to reduce their toxicity to humans.^41^ The size can be tailored for optimal efficacy, and capping agents can be used to prevent agglomeration, avoid undesirable nanoparticle oxidation, and enhance ion release. Commonly used capping agents are oleic acid, poly(acrylic acid), polyethylene glycol (PEG), poly(vinyl alcohol) (PVA), and polyvinylpyrrolidone (PVP).^42−45^

**Scheme 1:**
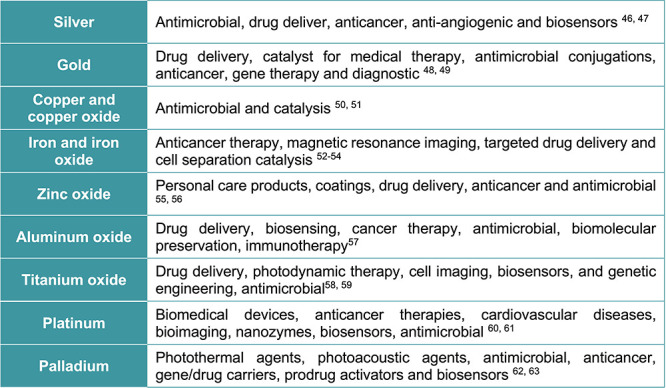
MNPs Used in Biomedical Applications

Therefore, the most important biomedical MNPs applied in antimicrobial formulations, which include silver, gold, copper, iron, zinc, titanium dioxide (TiO_2_), aluminum oxide (Al_2_O_3_), platinum, and palladium (Pd), are described in this section (Scheme 1). The most common experimental strategies used for their synthesis and surface functionalization are also depicted.

Overall, the application of MNPs in biomedicine presents several advantages and some limitations, particularly patients’ toxicity. Numerous challenges encompass a broad spectrum of fields of knowledge, such as biological, chemical, and materials engineering. A comprehensive approach to convert all the research generated information into suitable clinical practices is highly demanding. Nevertheless, the conjugation of tailored nanoparticles with other materials/molecules is an unlimited exploration field that could provide exceptional biomedical applications.

### 2.1 Silver Nanoparticles (AgNPs)

AgNPs are the most prevalent inorganic nanoparticles applied as antimicrobial agents. AgNPs have demonstrated high antimicrobial activity compared to that of the Ag ionic form. However, several concerns have emerged regarding their cytotoxicity. The toxicity mechanisms, long-term accumulation effects, and dose–response relationship are still grievously unknown.^64^

### 2.2 Gold Nanoparticles (AuNPs)

AuNPs are extremely valuable in developing antibacterial agents due to their low toxicity, high propensity for functionalization, eclectic effects, easy detection, and photothermal activity. AuNPs per se possess very low antimicrobial activity, but numerous studies on the antimicrobial activity of AuNPs conjugated with small molecules, such as drugs, vaccines, and antibodies, have been reported.^65−67^

### 2.3 Copper Nanoparticles (CuNPs)

CuNPs have also been widely researched due to their antimicrobial properties and higher biocompatibility. Copper, after silver, is one of the most commonly used nanomaterials due to its low cost and ready availability, although its synthesis remains challenging due to the high oxidation proneness of copper. Copper is susceptible to air oxidation, and its oxidized forms are thermodynamically more stable.^50^

### 2.4 Iron Oxide Nanoparticles (Fe_*x*_O_*y*_NPs)

The FDA approved Fe_3_O_4_/Fe_2_O_3_NPs in clinical applications mainly due to their high versatility in surface modification and stability. Iron oxides are the preferable nanomaterials in medical sciences since they display marginal toxicity, good biocompatibility, and excellent physicochemical properties such as superparamagnetism and stability in aqueous solutions. Nevertheless, the antimicrobial properties can only be observed at relatively high concentrations. Their activity can be adjusted by changing the surface potential, surface functional groups, and the iron oxidation state.^53,54,68^

### 2.5 Zinc Oxide Nanoparticles (ZnONPs)

ZnONPs are inexpensive, possess bactericidal properties, and have high biocompatibility with human skin.^69^ They have been presented as one of the most interesting and promising MNPs.^55,56^

### 2.6 Titanium Oxide Nanoparticles (TiO_2_NPs)

The antimicrobial activity of the TiO_2_NPs has been widely studied. It was found that the photocatalytic effect on TiO_2_ allows the inactivation of microorganisms due to its strong generation of radical oxygen species. One limitation of TiO_2_ is the activation mechanism. Photons with enough energy are required to activate the surface of these MNPs to promote the catalytic processes. Thus, the incorporation of dopants has been a strategy to improve the antibacterial performance of TiO_2_.^59,70−72^

### 2.7 Aluminum Oxide Nanoparticles (Al_2_O_3_NPs)

Al_2_O_3_NPs are low-cost, easy to handle, and effective against pathogenic microorganisms, including MDR bacteria. Nonetheless, the neurotoxicity and blood toxicity of the Al_2_O_3_NPs represent a concerning limitation. Thus, novel engineering strategies are needed to improve Al_2_O_3_NP biocompatibility.^57,73^

### 2.8 Platinum Nanoparticles (PtNPs)

PtNPs promote bacterial growth inhibition by catalyzing the hyperproduction of adenosine triphosphate (ATP). Although they have potential, the antimicrobial activity of PtNPs has been poorly studied. The PtNPs did not show any cytotoxicity, but further studies are still needed. The conjugation of PtNPs with other materials can be applied to develop novel applications that require control of bacterial growth.^60^

### 2.9 Palladium Nanoparticles (PdNPs)

The potential of PdNPs as an antimicrobial agent has been shown to be similar or superior to other MNPs and standard drugs (streptomycin and ampicillin) already in use.^62,63^ New studies involving these nanostructures need to be carried out to better understand the antimicrobial effect, the mechanism of action, and also possible toxic effects.

## 3 Metal Nanoparticle Synthesis

Generally, MNPs can be synthesized using two different approaches: (i) top-down, where the bulk material is reduced by sputtering, chemical etching, thermal ablation, and ball milling processes; (ii) bottom-up, where single atoms are accumulated via condensation, vapor deposition, sol–gel processes, spray pyrolysis, chemical or electrochemical deposition, aerosol methods, or reduction processes (electrochemical, chemical, biogenic, or photochemical reduction).^74^ To improve MNP stabilization and avoid aggregation, surface-stabilizing agents are commonly used. The synthesis process defines the physicochemical properties of nanoparticles, which governs their size, shape, surface charge, and oxidation state.^75−77^ These properties will considerably influence the interactions between MNPs and conjugated agents and, consequently, their antimicrobial performance and cytotoxicity.

Chemical reduction and sol–gel have been the most employed methods for MNP synthesis due to their simplicity. However, these protocols present high costs and are prone to generate toxic byproducts.^78^ The most common reducing agents in the chemical synthesis of MNPs may be replaced by biological materials such as bacteria, fungi, or plant extracts. Nanoparticles synthesized from biological materials are known as biogenic nanoparticles. Their main advantages are cost-effectiveness and negligible environmental impact.^79^ Nevertheless, the biosynthesis of MNPs currently still possess a high polydisperse index and low reproducibility.^80^

Therefore, methods for MNP synthesis should be carefully pondered to design the MNP properties according to the interactions required in the following steps.

## 4 Methods to Combine MNPs and Other Antimicrobial Agents

**Scheme 2:**
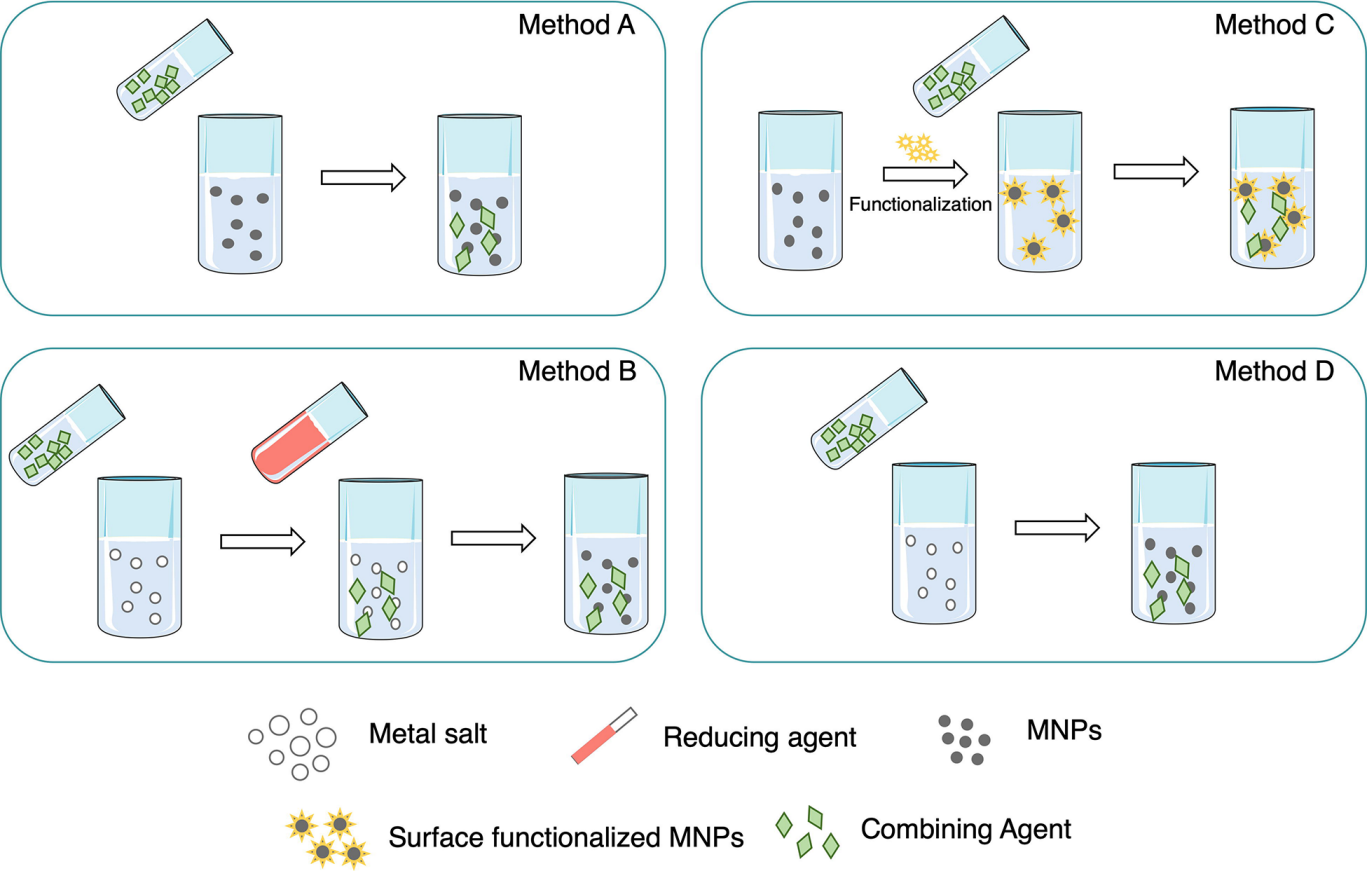
Common Methods to Conjugate MNPs and Antibiotics, Antifungals, or Antivirals

The preparation of MNP conjugates with antimicrobial drugs (including antibiotics, antifungals, and antivirals) is generally carried out via one of the following methods: method A, the MNPs’ synthesis and their posterior mixture with other agents’ solutions; method B, MNPs’ synthesis in the presence of the combining agents; method C, MNPs’ synthesis, subsequent functionalization, and posterior conjugation step; method D, MNPs’ synthesis using the conjugating agents also as reducing agents (Scheme 2).

In method A, the MNPs are synthesized, and the solutions containing the conjugating agents are prepared separately. Subsequently, both solutions are mixed and characterized (Scheme 2, method A).

In the case of method B, the MNPs’ synthesis occurs in the presence of conjugating agents. The conjugating agent may or not act as a reducing agent. However, it is always associated with another reducing agent during the synthesis (Scheme 2, method B).

Method C conjugates MNPs and antimicrobial compounds through a three-step preparation: (i) MNP synthesis, (ii) MNP surface functionalization; (iii) MNP mixing with conjugating agents (Scheme 2, method C). In this case, the MNP synthesis is an independent step that can be performed using any previously referred MNP preparation methods. Afterward, the MNPs’ surface is functionalized. The surface functionalization of metal and metal oxide nanoparticles has been used as a powerful tool to create bonds with organic molecules and biological cells, increasing the local concentration of MNPs in specific targets.^66^ MNPs were mostly modified by thiols, disulfides, amines, nitriles, carboxylic acids, and phosphines. Metal oxide nanoparticles were mainly functionalized by phosphonates or silanes. In addition, metal alkoxides, epoxides, metals, or metalloids can cover the NP surface to form an oxide film.^81,82^ Finally, the MNPs are mixed with the conjugating agents in the desired proportion.

In the last approach, method D, the conjugation is obtained in one single step. The synthesis of the MNPs unfolds using antimicrobials as reducing agents (Scheme 2, method D). In this case, the MNP synthesis is performed using fewer chemicals, but it requires a long reaction time. Hur et al. described the functionalization of AuNPs and AgNPs with ampicillin, where ampicillin simultaneously acted as the conjugating, stabilizing, and reducing agent.^83^

## 5 Therapeutic Agents Conjugated with MNPs and Antimicrobial Effect

Numerous works report the combination of MNPs and commercial antimicrobial drugs. Thus, this section is divided according to the drugs used: antibiotics, antifungals, and antivirals.

The antimicrobial methods to evaluate the synergistic effects between MNPs and other agents alone and in combination are mainly based on *in vitro* tests by calculating the inhibition zones (ZoIs), minimum inhibitory concentrations (MICs), and the colony reductions by plate counting techniques or through optical density (OD) measurements. The checkerboard method is the most common and is based on the calculation of the fractional inhibitory concentration (FIC) index obtained by dividing the MIC value of the combined antimicrobial agent by the MIC of the antimicrobial agent per se. When the FIC value is ≤0.5, the agents are considered synergic. FICs in the range of >0.5 to ≤1.0 are not synergistic or additive. FICs between >1.0 and ≤4.0 are negligible (indifferent), and FICs > 4.0 are antagonistic.^84^ This is a simple and effective procedure to assess synergistic effects. However, several literature references only depict MIC values and disregard FICs. Furthermore, other researchers estimate the synergism on the basis of the obtained ZoI.

Unfortunately, the calculation of synergism is obtained using a wide range of different methods making it difficult to compare various reports adequately.^31^ It is imperative to reach a consensus concerning the method used to calculate synergism between MNPs and antimicrobial drugs. Therefore, the development of a standard is urgently needed.

### 5.1 Antibiotics

Antibiotic resistance is recognized as one of the most critical threats to human health. The generalized overconsumption of broad-spectrum antibiotics, such as glycylcyclines, oxazolidinones, carbapenems, and polymyxins, has increased during the last years. Efforts are needed to revitalize the antibiotic pipeline and develop novel antibiotics effective against antibiotic-resistance pathogens.^85^ Antibiotic combinations are frequently used in clinical practice to circumvent antimicrobial resistance though little is known about their impact on the human body.^86^ The conjugation of antibiotics with MNPs could re-establish antibiotic capability to destroy resistant bacteria. MNP–antibiotic combinations have shown an increase in the concentration of antibiotics at their interaction site on bacteria.^31^ The combination of MNPs with antibiotics is the most documented compared to that with other agents (87 studies in the total of 111 reports), and all classes of antibiotics may be found (Table 1).

**Table 1:** Antibiotics and Corresponding Classes Used in Synergistic Studies with MNPs

β-lactams		macrolides	quinolones	aminoglycosides
amoxicillin	ceftriaxone	azithromycin	ciprofloxacin	amikacin
amoxicillin/clavulamic acid	cefuroxime	clindamycin	enoxacin	gentamicin
ampicillin	cephalexin	erythromycin	levoflaxacin	kanamycin
aztreonam	cephalothin	nitrofurantoin	nalidixic acid	neomycin
biapenem	cephazolin	rifampicin	ofloxacin	streptomycin
carbenicillin	feropenem	oleandomycin	oxolinic acid	
cefaclor	imipenem			
cefazolin	meropenem			
cefepime	methicillin			
cefoperazone	oxacillin			
cefotaxime	penicillin			
cefoxitin	penicillin G			
cefpodoxime	piperacillin			
ceftazidime				

glycopeptides	sulfonamides	tetracyclines	polymixins	others
norvancomycin	co-trimoxazole	doxycycline	colistin	bacitracin
teicoplanin	trimethoprim	oxytetracycline	polymyxin B	chloramphenicol
vancomycin	sulfanilamide	tetracycline		fosfomycin
		tigecycline		fusidic acid
				lincomycin
				novobiocin

**Chart 2:**
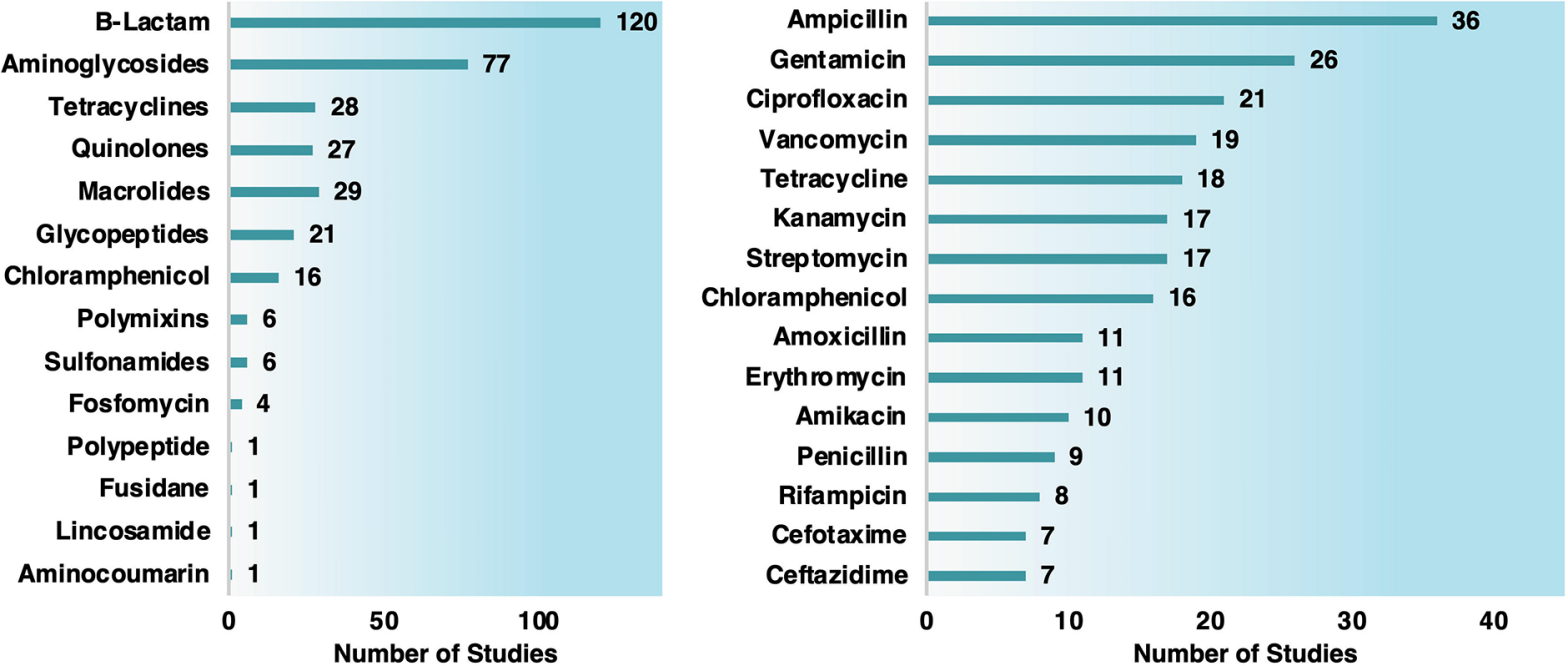
Number of Studies Organized by Antibiotic Class (Left Graph) and Antibiotic Type (Right Graph) Conjugated with MNPs

**Chart 3:**
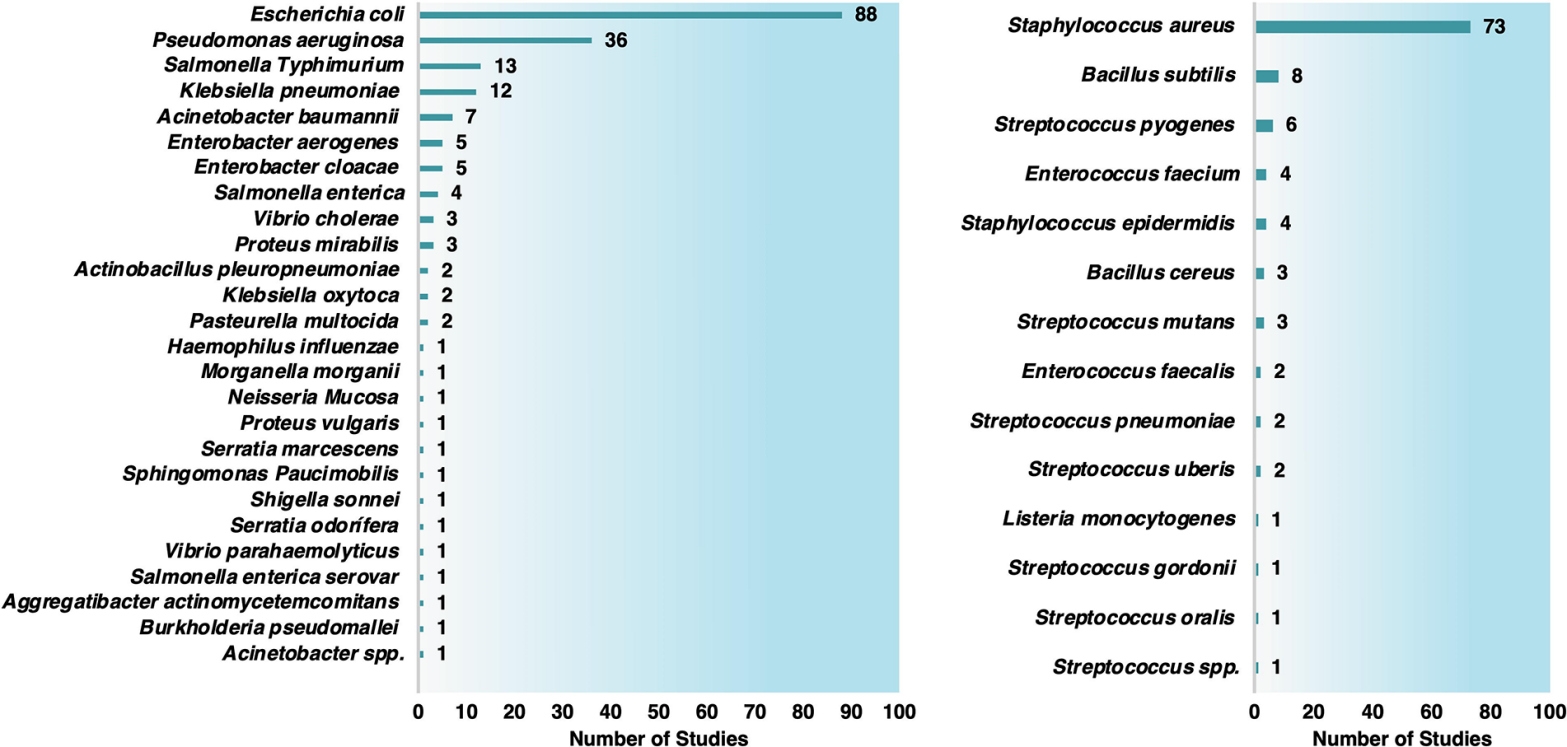
Number of Studies Involving Antibiotics and MNPS against Gram-Negative (Left Graph) and Gram-Positive Bacteria (Right Graph)

β-Lactams and aminoglycosides were the most common antibiotics used in synergistic tests (Chart 2). Studies with Gram-negative bacteria were the most prevalent (191 studies), encompassing *Escherichia coli* (88 studies), *Pseudomonas aeruginosa* (36 reports), *Salmonella typhimurium* (13 studies), and *Klebsiella pneumoniae* (12 documents) (Chart 3). The Gram-positive studies (107) were mainly focused on *Staphylococcus aureus* (73 studies), including multiresistant *S. aureus* (MRSA).

Regarding the MNPs conjugated to antibiotics, only AgNPs (61 studies), AuNPs (13 studies), ZnNPs or ZnONPs (11 studies), CuNPs or CuONPs (6 studies), PtNPs (2 studies), and FeNPs (1 study) were tested for synergistic activity. The referred research works in the following sections are presented according to the conjugation method, MNP synthesis method, and MNP type. Some examples are described for each type of MNPs, and a particular focus was given to the antimicrobial results and characterization methods when available.

#### 5.1.1 Method A

Among the different strategies, method A (Section 4, Scheme 2) using MNPs’ synthesis and their posterior mixture with antibiotics solutions is the most used method to conjugate MNPs and antibiotics (Table 2).

**Table 2:** Synergic Studies between MNPs and Antibiotics Obtained by the MNPs’ Synthesis and Their Posterior Combination with Antibiotics: Method A

MNPs, size (nm)	MP synthesis	reducing agent	stabilizing agent	combined antibiotics	bacterial strains	test method/synergic results	ref
Ag, 3.0	chemical	n.a.	n.a.	ampicillin, chloramphenicol, and kanamycin	*E. faecium*, *S. aureus*, *S. mutans*, *E. coli*, and *P. aeruginosa*	FIC: *E. faecium*, ampicillin and chloramphenicol; *S. mutans*, ampicillin and kanamycin; *E. coli*, ampicillin and kanamycin; *P. aeruginosa*, chloramphenicol and kanamycin	(87)
Ag, 5.0–12.0	chemical	sodium borohydride and trisodium citrate	trisodium citrate	polymyxin B, rifampicin, and tigecycline	resistant *A. baumannii* strain	FIC: *A. baumannii*, polymyxin B, and rifampicin	(88)
Ag, 8.0	chemical	d-maltose and sodium borohydride	gelatin	amoxycillin, penicillin G, gentamicin, and colistin	*S. enterica*, *S. aureus*, *E. coli*, *A. pleuropneumoniae*, *P. multocida*, and *S. uberis*	FIC: *A. pleuropneumoniae*, penicillin G; *E. coli*, colistin; *S. aureus*, gentamicin	(89)
Ag, 8.6	chemical	gallic acid	gallic acid	ampicillin and amikacin	clinical isolates (*E. faecium*, *S. aureus*, *A. baumannii*, *E. cloacae*, three different isolates of *E. coli*, *K. pneumoniae*, *M. morgannii*, and *P. aeruginosa*)	FIC: *E. faecium*, *A. baumannii*, *K. pneumoniae*, *M. morganii*, and *P. aeruginosa*, ampicillin and amikacin; *S. aureus*, *E. coli*, and *E. cloacae*, amikacin	(90)
Ag, 10.0	chemical	sodium borohydride, trisodium citrate dihydrate, and hydrazine	PVP and trisodium citrate dihydrate	cephalexin nanoparticles (96 nm)	*S. aureus*	ZoI: *S. aureus*, cephalexin	(91)
Ag, 16.0	chemical	sodium borohydride and trisodium citrate dihydrate	PVP	streptomycin, ampicillin, and tetracycline	*E. coli* and *S. aureus*	ZoI: *E. coli*, streptomycin, ampicillin, and tetracycline; *S. aureus*, streptomycin, ampicillin, and tetracycline	(92)
Ag, 19.3	chemical	sodium borohydride and trisodium citrate dihydrate	SDS	streptomycin, ampicillin, and tetracycline	*E. coli* and *S. aureus*	ZoI: *E. coli*, streptomycin, ampicillin, and tetracycline; *S. aureus*, streptomycin, ampicillin, and tetracycline	(92)
Ag, 20.0	chemical	sodium citrate	sodium citrate	ampicillin	*S. aureus*, *S. epidermidis*, *E. coli*, *P. aeruginosa*, and *K. pneumoniae*	MIC: *E. coli*, *K. pneumoniae*, and *P. aeruginosa*, ampicillin	(93)
Ag, 20.0	chemical	ascorbic acid	n.a.	amoxicillin	*E. coli*	MIC: *E. coli*, amoxicillin	(94)
Ag, 20.0 nm	chemical	sodium borohydride	PVP	vancomycin and amikacin	*S. aureus* and *E. coli*	ZoI: all combinations	(95)
Ag, 20.0–40.0 nm	chemical	Tween 80	Tween 80	gentamicin	*S. epidermidis*	FIC: synergism	(96)
Ag, 23.0	chemical	sodium citrate	sodium citrate	tetracycline, neomycin, and penicillin G	*S. typhimurium*	MIC and inhibition (plate counting): *S. typhimurium*, tetracycline, neomycin, and penicillin G	(97)
Ag, 25.0	chemical	ethylene glycol	PVP	gentamicin	*E. coli* and *S. aureus*	FIC: *E. coli* and *S. aureus*, gentamicin	(98)
Ag, 25.0	chemical	sodium citrate	sodium citrate	vancomycin	*S. aureus* and *E. coli*	ZoI: all combinations	(99)
Ag, 26.0	chemical	d-glucose	starch	erythromycin, ampicillin, chloramphenicol, cephalothin, clindamycin, tetracycline, gentamycin, amoxicillin, ciprofloxacin, ampicillin, cefpodoxime, and cefuroxime	*S. aureus*, MRSA, *S. mutans*, *S. oralis*, *S. gordonii*, *E. faecalis*, *E. coli*, *A. actinomycetemcomitans*, and *P. aeruginosa*	ZoI: *S. aureus*, erythromycin, clindamycin, and tetracycline; *S. mutans*, gentamycin, amoxicillin, ciprofloxacin, cefpodoxime, and cefuroxime; *S. gordonii*, erythromycin, cephalothin, clindamycin, and tetracycline; *A. actinomycetemcomitans*, all combinations; *P. aeruginosa*, erythromycin, chloramphenicol, and ciprofloxacin	(100)
Ag, 26.0	chemical	d-maltose in alkaline media	gelatin	ampicillin, ampicillin/sulbactam, cefazolin, cefuroxime, cefoxitin, gentamicin, co-trimoxazole, colistin, oxolinic acid, ofloxacin, tetracycline, aztreonam, piperacillin, piperacillin/tazobactam, meropenem, ceftazidime, cefoperazone, cefepime, amikacin, ciprofloxacin, penicillin, oxacillin, chloramphenicol, erythromycin, clindamycin, ciprofloxacin, teicoplanin, and vancomycin	*E. coli*, *P. aeruginosa*, and *S. aureus*	MIC: *E. coli*, ampicillin, ampicillin/sulbactam, aztreonam, cefazolin, cefoxitin, cefuroxime, cotrimoxazole, colistin, gentamicin, ofloxacin, oxolinic acid, and tetracycline; *P. aeruginosa*, amikacin, aztreonam, cefepime, cefoperazone, ceftazidime, ciprofloxacin, colistin, gentamicin, meropenem, ofloxacin, piperacillin, and piperacillin/tazobactam; *S. aureus*, ampicillin/sulbactam, chloramphenicol, ciprofloxacin, clindamycin, cotrimoxazole, erythromycin, gentamicin, oxacillin, penicillin, teicoplanin, tetracycline, and vancomycin	(101)
Ag, 28.0	chemical	d-maltose and sodium borohydride	gelatin	amoxycillin, penicillin G, gentamicin, and colistin	*S. enterica*, *S. aureus*, *E. coli* eae+, *A. pleuropneumoniae*, *P. multocida*, and *S. uberis*	FIC: *A. pleuropneumoniae*, amoxycillin and gentamicin; *E. coli*, gentamicin; *S. aureus*, gentamicin	(89)
Ag, 28.0	chemical	d-maltose	d-maltose	cefotaxime, ceftazidime, meropenem, ciprofloxacin, and gentamicin	susceptible and resistant *E. coli* and *K. pneumoniae*	FIC: synergism in all resistant strains except to *K. pneumoniae* carbapenemase (additive effect)	(102)
Ag, 29.8	chemical	sodium citrate	sodium citrate	ampicillin, penicillin, enoxacin, kanamycin, neomycin, and tetracycline	*S. typhimurium*	colony counting: all combinations	(103)
Ag, 29.8	chemical	sodium citrate	sodium citrate	neomycin, kanamycin, enoxacin, and tetracycline	multidrug-resistant *S. typhimurium*	inhibition (plate counting): *S. typhimurium*, enoxacin, kanamycin, neomycin, and tetracycline	(103)
Ag, 38.3	chemical	sodium borohydride and trisodium citrate dihydrate	trisodium citrate dihydrate	streptomycin, ampicillin, and tetracycline	*E. coli* and *S. aureus*	ZoI: *E. coli*, streptomycin, ampicillin, and tetracycline; *S. aureus*, streptomycin, ampicillin, and tetracycline	(92)
Ag, 70.0	chemical	trisodium citrate	trisodium citrate	vancomycin	*S. aureus* and *E. coli*	ZoI: all combinations	(99)
Au, 15.0–20.0	chemical	trisodium citrate	trisodium citrate	ciprofloxacin	n.a.	n.a.	(104)
CuO, 15.0	chemical	hydrazine	polyethylene glycol	meropenem and ciprofloxacin	multidrug-resistant *P. aeruginosa*	FIC: synergism using ciprofloxacin and additive using meropenem	(105)
Fe and Cu, 6.0–9.0	chemical	hydrazine hydrate and sodium borohydride	n.a.	gentamicin	*E. coli*, *P. aeruginosa*, and *B. cereus*	ZoI: synergism	(106)
ZnO, 15.0	chemical	sol–gel with potassium hydroxide	n.a.	cefotaxime, ampicillin, ceftriaxone, and cefepime	*E. coli*, *K. pneumoniae*, *S. paucimobilis*, and *P. aeruginosa*	ZoI: *E. coli*, cephotaxime, ampicillin, ceftriaxome, and cefepime; *K. pneumoniae*, cephotaxime, ceftriaxome, and cefepime; *S. paucimobilis*, ampicillim and cefepime; *P. aeruginosa*, cephotaxime, ampicillin, and cefepime	(107)
ZnO, 35.0	chemical	polyethylene glycol	polyethylene glycol	meropenem and ciprofloxacin	multidrug-resistant *P. aeruginosa*	FIC: synergism with ciprofloxacin and additive for meropenem	(105)
ZnO, 47.6	chemical	sodium hydroxide	gelatin	cefazolin	*S. aureus*	MIC: *S. aureus*, cefazolin	(108)
Mg-doped ZnO, 33.0	chemical	PEG and sodium hydroxide	PEG	chloramphenicol	*E. aerogens*, *S. aureus*, *E. lentum*, and *P. vulgaris*	ZoI: *E. aerogens*, *S. aureus*, *E. lentum*, and *P. vulgaris*, chloramphenicol	(109)
Ag–Au, 27.5	chemical	sodium hydroxide	sodium acrylate	doxycycline	*E. coli*, *S. aureus*, *P. aeruginosa*, and *M. luteus*	ZoI: *E. coli*, *S. aureus*, *P. aeruginosa*, and *M. luteus*, doxycycline	(110)
Cu–Zn, 21.0	chemical	triethylene glycol	triethylene glycol	meropenem and ciprofloxacin	multidrug-resistant *P. aeruginosa*	FIC: all combinations	(105)
ZnO, 20.0–45.0	mechano-chemical–milling process	n.a.	n.a.	ciprofloxacin	*S. aureus* and *E. coli* clinical isolates	ZoI: *S. aureus* and *E. coli*, ciprofloxacin	(111)
Ag, 5.0–20.0	biogenic, bacteria	*Streptomyces* calidiresistants IF17 strain	biomolecules from actinobacterial strains	ampicillin, kanamycin, and tetracycline	*E. coli*, *S. aureus*, and *B. subtilis*	FIC: *E. coli*, tetracycline; *S. aureus*, ampicillin, kanamycin, and tetracycline; *B. subtilis*, ampicillin, kanamycin, and tetracycline	(112)
Ag, 5.0–32.0	biogenic, bacteria	biomass from *Klebsiella pneumoniae*	protein caps from biomass	penicillin, amoxicillin, erythromycin, and vancomycin	clinical isolates of *S. aureus* and *E. coli*	ZoI: *E. coli*, amoxicillin, erythromycin, penicillin, and vancomycin; *S. aureus*, amoxicillin, erythromycin, penicillin, and vancomycin	(113)
Ag, 5.0–50.0	biogenic, bacteria	*Streptomyces* calidiresistants IF11 strain	biomolecules from actinobacterial strains	ampicillin, kanamycin, and tetracycline	*E. coli*, *S. aureus*, and *B. subtilis*	FIC: *B. subtilis*, kanamycin	(112)
Ag, 17.0	biogenic, bacteria	*Actinomycetes* strain	protein caps from biomass	ampicillin	*S. aureus*, *S. epidermidis*, *E. coli*, *P. aeruginosa*, and *K. pneumoniae*	MIC and ZoI: *E. coli*, *K. pneumoniae*, and *P. aeruginosa*, ampicillin	(93)
Ag, 20.0	biogenic, bacteria	biomass from *Klebsiella pneumoniae*	protein caps from biomass	chloramphenicol, gentamicin, and chloramphenicol/gentamicin	*E. faecalis*	ZoI: *E. faecalis*, chloramphenicol, chloramphenicol/gentamicin, and gentamicin	(114)
Ag, 35.0–60.0	biogenic, bacteria	silver-resistant estuarine *P. aeruginosa* strain	biomass from bacteria	ampicillin and ciprofloxacin	resistant *S. aureus* strain VN3 and ciprofloxacin-resistant *V. cholera* strain VN1	ZoI: all combinations	(115)
Ag–Au, 5.0–50.0	biogenic, bacteria	*Pseudomonas veronii* strain AS41G inhabiting *Annona squamosa L.*	biomolecules from *Pseudomonas veronii strain* AS41G	bacitracin, chloramphenicol, erythromycin, gentamicin, kanamycin, and streptomycin	*B. subtilis*, *E. coli*, *K. pneumoniae*, and *S. aureus*	ZoI: all combinations	(116)
Ag, 5.0–30.0	biogenic, fungal	biomass from *Aspergillus flavus*	protein molecules from biomass	imipenem, gentamicin, vancomycin, and ciprofloxacin	*E. coli*, *P. aeruginosa*, and *E. faecalis* resistant to trimethoprim, vancomycin, and ciprofloxacin; *S. aureus* resistant to trimethoprim and vancomycin; *M. luteus* resistant to trimethoprim, gentamycin, and vancomycin; *A. baumanii* resistant to imipenem, trimethoprim, gentamycin, and vancomycin;*K. pneumoniae* and *Bacillus* spp. resistant to trimethoprim	ZoI: *A. baumanii*, ciprofloxacin; *Bacillus* spp., ciprofloxacin, gentamicin, imipenem, and vancomycin; *E. faecalis*, imipenem; *E. coli*, imipenem; *K. pneumoniae*, ciprofloxacin, gentamicin, imipenem, and vancomycin; *M. luteus*, ciprofloxacin, gentamicin, imipenem, and vancomycin; *P. aeruginosa*, ciprofloxacin, gentamicin, imipenem, and vancomycin; *P. aeruginosa*, gentamicin, imipenem, and vancomycin; *S. aureus*, gentamycin	(117)
Ag, 5.0–40.0	biogenic, fungal	biomass from *Trichoderma viride*	protein molecules from biomass	erythromycin, kanamycin, chloramphenicol, and ampicillin	*S. typhi*, *E. coli*, *S. aureus*, and *M. luteus*	ZoI: *E. coli*, ampicillin, chloramphenicol, erythromycin, and kanamycin; *M. luteus*, ampicillin, chloramphenicol, and kanamycin; *S. typhi*, ampicillin, chloramphenicol, erythromycin, and kanamycin; *S. aureus*, ampicillin, chloramphenicol, erythromycin, and kanamycin	(118)
Ag, 8.0–12.0	biogenic, fungal	enzymes such as nitrate reductase and phytochelatin synthase from *Acinetobacter calcoaceticus*	biomolecules secreted by the cells	amikacin, gentamicin, kanamycin, amoxicillin, ampicillin, penicillin, ceftazidime, ceftriaxone, vancomycin, ciprofloxacin, doxycycline, tetracycline, chloramphenicol, and trimethoprim	*E. aerogenes*, *E. coli*, *P. aeruginosa*, *S. sonnie*, *S. typhimurium*, *S. aureus*, *S. mutans*, and *A. baumannii*	ZoI or MIC: *A. baumannii*, amikacin, amoxicillin, ampicillin, chloramphenicol, ciprofloxacin, doxycycline, gentamicin, tetracycline, trimethoprim, and vancomycin; *E. aerogenes*, amikacin, amoxicillin, ampicillin, ceftriaxone, chloramphenicol, ciprofloxacin, doxycycline, gentamicin, kanamycin, penicillin, tetracycline, trimethoprim, and vancomycin; *E. coli*, amikacin, amoxicillin, ampicillin, ceftazidime, ceftriaxone, chloramphenicol, ciprofloxacin, doxycycline, gentamicin, kanamycin, penicillin, tetracycline, trimethoprim, and vancomycin; *P. aeruginosa*, amikacin, amoxicillin, ampicillin, ceftazidime, ceftriaxone, chloramphenicol, ciprofloxacin, doxycycline, gentamicin, kanamycin, penicillin, tetracycline, trimethoprim, and vancomycin; *S. typhimurium*, amikacin, ampicillin, ceftazidime, ceftriaxone, chloramphenicol, ciprofloxacin, doxycycline, gentamicin, kanamycin, penicillin, tetracycline, trimethoprim, and vancomycin; *S. sonnie*, amikacin, amoxicillin, ampicillin, ceftazidime, ceftriaxone, ciprofloxacin, chloramphenicol, ciprofloxacin, doxycycline, gentamicin, kanamycin, tetracycline, trimethoprim, and vancomycin; *S. aureus*, amikacin, amoxicillin, ampicillin, ceftazidime, ceftriaxone, chloramphenicol, ciprofloxacin, doxycycline, gentamicin, kanamycin, penicillin, tetracycline, trimethoprim, and vancomycin; *S. mutans*, amikacin, amoxicillin, ampicillin, ceftazidime, ceftriaxone, chloramphenicol, ciprofloxacin, doxycycline, kanamycin, penicillin, tetracycline, trimethoprim, and vancomycin	(119)
Ag, 30.0–70.0	biogenic, fungal	biomass from *Cryphonectria* sp.	n.a.	streptomycin and amphotericin	*S. aureus*, *S. typhi*, and *E. coli*	ZoI: *S. aureus*, *S. typhi*, and *E. coli*, streptomycin	(120)
Ag, 66.7	biogenic, fungal	biomass from *Emericella nidulans*	biomolecules from biomass	amikacin, kanamycin, oxytetracycline, and streptomycin	*E. coli*, *P. aeruginosa*, and *S. aureus*	FIC: *E. coli*, amikacin and streptomycin	(121)
Ag, 81.1	biogenic, fungal	biomass from *Aspergillus flavus*	biomolecules from biomass	amikacin, kanamycin, oxytetracycline, and streptomycin	*E. coli*, *P. aeruginosa*, and *S. aureus*	FIC: *E. coli*, amikacin and streptomycin; *S. aureus*, kanamycin, oxytetracycline, and streptomycin	(121)
Ag, 2.0	biosynthesis, plant	*Dioscorea bulbifera* tuber extract	*Dioscorea bulbifera* tuber extract	streptomycin, rifampicin, chloramphenicol, novobiocin, and ampicillin	*E. coli*, *P. aeruginosa*, and *S. aureus*	ZoI: all combinations	(122)
Ag, 2.0	biosynthesis, plant	*Dioscorea bulbifera* tuber extract	*Dioscorea bulbifera* tuber extract	streptomycin, rifampicin, chloramphenicol, novobiocin, and ampicillin	*E. coli*, *P. aeruginosa*, and *S. aureus*	ZoI: all combinations	(122)
Ag, 5.0–30.0	biosynthesis, plant	extract from *Dioscorea bulbifera*	protein molecules from biomass	amikacin, gentamycin, kanamycin, streptomycin, amoxicillin, ampicillin, penicillin, piperacillin, feropenem, ceftazidime, ceftriaxone, cefotaxime, polymyxin, vancomycin, erythromycin, nalidixic acid, rifampicin, tetracycline, doxycycline, chloramphenicol, nitrofurantoin, and trimethoprim	*A. baumannii*, *E. cloacae*, *E. coli*, *H. influenzae*, *K. pneumoniae*, *N. mucosa*, *P. mirabilis*, *P. aeruginosa*, *S. typhi*, *Serratia odorífera*, *V. parahemolyticus*, *B. subtilis*, and *S. aureus*	ZoI: *A. baumannii*, amoxicillin, ampicillin, cefotaxime, erythromycin, gentamycin, kanamycin, nalidixic acid, nitrofurantoin, penicillin, piperacillin, rifampicin, and rimethoprim; *B. subtilis*, ampicillin, cefotaxime, chloramphenicol, nalidixic acid, nitrofurantoin, penicillin, piperacillin, streptomycin, trimethoprim, and vancomycin; *E. cloacae*, amikacin, amoxicillin, erythromycin, nalidixic acid, and penicillin; *E. coli*, amikacin, erythromycin, kanamycin, nalidixic acid, polymyxin, streptomycin, and trimethoprim; *H. influenzae*, cefotaxime, ceftriaxone, nitrofurantoin, and trimethoprim; *K. pneumoniae*, amoxicillin, ampicillin, chloramphenicol, erythromycin, feropenem, nitrofurantoin, penicillin, rifampicin, trimethoprim, and vancomycin; *N. mucosa*, amikacin, ampicillin, erythromycin, feropenem, gentamycin, nitrofurantoin, penicillin, polymyxin, tetracycline, trimethoprim, and vancomycin; *P. mirabilis*, erythromycin, nalidixic acid, and vancomycin; *P. aeruginosa*, amikacin, amoxicillin, ampicillin, chloramphenicol, doxycycline, erythromycin, feropenem, gentamycin, kanamycin, nalidixic acid, nitrofurantoin, penicillin, streptomycin, trimethoprim, and vancomycin; *S. typhi*, amikacin, amoxicillin, ampicillin, cefotaxime, ceftriaxone, chloramphenicol, erythromycin, gentamycin, kanamycin, nalidixic acid, nitrofurantoin, penicillin, piperacillin, polymyxin, streptomycin, trimethoprim, and vancomycin; *Serratia odorífera*, ceftazidme, erythromycin, nalidixic acid, nitrofurantoin, trimethoprim, and vancomycin; *S. aureus*, amikacin, amoxicillin, ampicillin, ceftazidme, erythromycin, kanamycin, nalidixic acid, polymyxin, streptomycin, and trimethoprim; *V. parahemolyticus*, ampicillin, cefotaxime, ceftriaxone, kanamycin, nalidixic acid, nitrofurantoin, polymyxin, and trimethoprim	(123)
Ag, 5.0–40.0	biosynthesis, plant	extract of *Argyreia nervosa*	organic molecules from leaf extract	vancomycin, streptomycin, tetracycline, gentamicin, amoxicillin/clavulamic acid, erythromycin, and ciprofloxacin	*S. aureus* and *E. coli*	ZoI: *S. aureus*, amoxicillin/clavulamic acid, ciprofloxacin, erythromycin, gentamicin, streptomycin, tetracycline, and vancomycin; *E. coli*, amoxicillin/clavulamic acid, erythromycin, streptomycin, tetracycline, and vancomycin	(124)
Ag, 5.8	biosynthesis, plant	gum kondagogu	gum kondagogu	streptomycin, gentamicin, and ciprofloxacin	*S. aureus*, *S. aureus*, *E. coli*, and *P. aeruginosa*	FIC: *S. aureus*, gentamicin and streptomicin; *S. aureus*, streptomicin; *E. coli*, streptomicin; *P. aeruginosa*, streptomicin	(125)
Ag, 7.4–18.3	biosynthesis, plant	*Rosa damascenes* extract	*Rosa damascenes* extract	cefotaxime	*E. coli* and MRSA	ZoI: all combinations	(126)
Ag, 15.0	biosynthesis, plant	extract from *Ulva fasciata*	n.a.	azithromycin, gentamicin, oxacillin, cefotaxime, neomycin, ampicillin/sulbactam, cefuroxime, fosfomycin, chloramphenicol, and oxytetracycline	*S. aureus*, *S. enterica*, and *E. coli*	ZoI: *E. coli*, cefotaxime, cefuroxime, fosfomycin, chloramphenicol, azithromycin, and gentamicin; *S. enterica*, azithromycin, gentamicin, oxacillin, cefotaxime, neomycin, ampicillin/sulbactam, cefuroxime, fosfomycin, chloramphenicol, and oxytetracycline; *S. aureus*, azithromycin, oxacillin, cefotaxime, neomycin, ampicillin/sulbactam, cefuroxime, fosfomycin, chloramphenicol, and oxytetracycline	(127)
Ag, 15.0–20.0	biosynthesis, plant	*Eurotium cristatum* extract	*Eurotium cristatum* extract	vancomycin, oleandomycin, ceftazidime, rifampicin, penicillin G, neomycin, cephazolin, novobiocin, carbenicillin, lincomycin, tetracycline, and erythromycin	*C. albicans*, *P. aeruginosa*, and *E. coli*	ZoI: all combinations	(128)
Ag, 20.0–30.0	biosynthesis, plant	extract from *Urtica dioica Linn.*	extract from *Urtica dioica Linn.*	streptomycin, amikacin, kanamycin, vancomycin, tetracycline, ampicillin, cefepime, amoxicillin, and cefotaxime	*B. cereus*, *S. epidermidis*, *S. aureus*, *B. subtilis*, *E. coli*, *S. typhimurium*, *K. pneumoniae*, and *S. marcescens*	ZoI: *B. cereus*, streptomycin, amikacin, kanamycin, vancomycin, tetracycline, ampicillin, cefepime, amoxicillin, and cefotaxime; *S. epidermidis*, streptomycin, amikacin, kanamycin, tetracycline, ampicillin, cefepime, and amoxicillin; *S. aureus*, streptomycin, amikacin, kanamycin, vancomycin, tetracycline, cefepime, amoxicillin, and cefotaxime; *B. subtilis*, streptomycin, amikacin, kanamycin, vancomycin, tetracycline, ampicillin, cefepime, amoxicillin, and cefotaxime; *E. coli*, streptomycin, amikacin, vancomycin, tetracycline, ampicillin, cefepime, amoxicillin, and cefotaxime; *S. typhimurium*, streptomycin, amikacin, kanamycin, vancomycin, tetracycline, ampicillin, cefepime, amoxicillin, and cefotaxime; *K. pneumoniae*, streptomycin, amikacin, kanamycin, vancomycin, tetracycline, ampicillin, cefepime, amoxicillin, and cefotaxime; *S. marcescens*, streptomycin, kanamycin, tetracycline, ampicillin, amoxicillin, and cefotaxime	(129)
Ag, 45.3	biosynthesis, plant	*Zea may* extract	extracts of corn leaves	kanamycin and rifampicin	*B. cereus*, *L. monocytogenes*, *S. aureus*, *E. coli*, and *S. Typhimurium*	ZoI: *B. cereus*, *E. coli*, *L. monocytogenes*, *S. Typhimurium*, and *S. aureus*, kanamycin and rifampicin	(130)
Cu, 22.7	biosynthesis, plant	extracts from *Zingiber* and *Allium* sp.	extracts from *Zingiber* and *Allium* sp.	doxycycline	*P. aeruginosa* and *E. coli*	ZoI: synergism	(131)
CuO, 40.0–50.0	biosynthesis, plant	aqueous extract of *Tamarindus indica L.*	aqueous extract of *Tamarindus indica L.*.	amoxiclav	*P. mirabilis* and *S. aureus*	FIC: synergism	(132)
ZnO, 66.0	biosynthesis, plant	*Ficus carica* plant extract	phytochemicals from plant extract	*E. coli*, gentamicin, erythromycin, and fosfomycin; *P. aeroginosa*, gentamicin, amikacin, and ciprofloxacin; *S. aureus*, fusidic acid, oxacillin, and rifampicine; *Acinetobacter*, tigecycline, amikacin, and rifampicine; *P. mirabilis*, gentamicin, erythromycin, and fosfomycin	*E. coli*, *P. aeroginosa*, *S. aureus*, *Acinetobacter*, and *P. mirabilis*	ZoI: all combinations	(133)
ZnO, 187.0	biosynthesis, plant	*Ulva fasciata* alga extract	n.a.	azithromycin, gentamicin, oxacillin, cefotaxime, neomycin, ampicillin/sulbactam, cefuroxime, fosfomycin, chloramphenicol, and oxytetracycline	*S. aureus*, *S. enterica* subsp. *Bukuru*, and *E. coli*	ZoI: *E. coli*, azithromycin, oxacillin, cefotaxime, ampicillin/sulbactam, cefuroxime, fosfomycin, and oxytetracycline; *S. enterica*, azithromycin, gentamicin, oxacillin, cefotaxime, neomycin, ampicillin/sulbactam, cefuroxime, fosfomycin, chloramphenicol, and oxytetracycline; *S. aureus*, azithromycin, oxacillin, cefotaxime, neomycin, ampicillin/sulbactam, cefuroxime, fosfomycin, chloramphenicol, and oxytetracycline	(127)
ZnO, 200.0	biosynthesis, plant	*Pongamia pinnata* leaf extract	*Pongamia pinnata* leaf extract	eErythromycin	*P. aeruginosa*	ZoI: *P. aeruginosa*, erythromycin	(134)
Ag–Pt, 2.0	biosynthesis, plant	*Dioscorea bulbifera* tuber extract	*Dioscorea bulbifera* tuber extract	streptomycin, rifampicin, chloramphenicol, novobiocin, and ampicillin	*E. coli*, *P. aeruginosa*, and *S. aureus*	ZoI: all combinations except *P. aeruginosa* with novobiocin	(122)
Ag, 10.0–15.0	commercial	n.a.	n.a.	ampicillin, kanamycin, gentamycin, and clindamycin	*A. baummannii*	optical density: all combinations	(135)
Ag, 15.2	commercial	n.a.	starch	ceftazidime, imipenem, meropenem, and gentamicin sulfate	clinical isolates (3) of *B. pseudomallei*	FIC: all combinations with the exception of one isolate with ceftazidime and imipenem	(136)
Ag, 35.0	commercial	n.a.	PVP	chloramphenicol, kanamycin, biapenem, and aztreonam	*E. coli*, *S. enterica* serovar *S. Typhimurium*, *S. aureus*, and *B. subtilis*	FIC: *E. coli*, *S. typhimurium*, and *S. aureus*, kanamycin	(137)
Ag, 10.0	n.a.	n.a.	PVP	ampicillin	MRSA	colony counting: synergism	(138)
ZnO, 17.1	solvothermal	glycerol	ammonium citrate	ciprofloxacin and ceftazidime	*A. baumannii*	ZoI: all combinations	(139)

In the research works combining AgNPs and antibiotics, AgNPs were mainly obtained by chemical or biochemical reduction with particle sizes varying between 2.0 and 81.0 nm. The AgNPs obtained using bacteria, fungi, or plants presented a higher polydispersity index (PdI) when compared with the chemically synthesized nanoparticles. It should underscore the favorable antimicrobial properties achieved by combining AgNPs with commercial antibiotics, even against MDR strains.

Wan et al. reported a synergistic effect using AgNPs combined with the antibiotics polymixin B and rifampicin and an additive effect using AgNP–tigecycline. *In vivo* tests found that AgNP–antibiotic combinations led to superior survival ratios in *A. baumannii*-infected mouse peritonitis.^88^ Smekalova et al. performed 40 different combination tests, where 7, 17, and 16 were synergistic, additive, and indifferent, respectively. None of the tested combinations showed an antagonistic effect. The majority of the synergistic effects were observed for the combinations of AgNPs with gentamicin. However, the highest enhancement of antibacterial activity was found in the combined therapy with penicillin G against *A. pleuropneumoniae.* Moreover, *A. pleuropneumoniae* and *P. multocida*, which are resistant to amoxicillin, gentamicin, and colistin, were sensitive to these antibiotics when combined with AgNPs.^89^

Lopez-Carrizales et al. tested the activity of two classes of conventional antimicrobial agents (ampicillin and amikacin) alone and in combination with AgNPs against a set of ten MDR clinical isolates and two reference strains. The authors indicate that infections caused by MDR microorganisms could be treated using a synergistic combination of antimicrobial drugs and AgNPs. In this case, the combination of AgNPs with antibiotics promotes a decrease in the size of the nanoparticles, observed in transmission electron microscopy (TEM), from 8.57 ± 1.17 nm to 4.01 ± 0.80 nm using ampicillin and 6.03 ± 0.87 nm using amikacin. The dynamic light scattering (DLS) and zeta potential results showed more stable nanoparticles when combined with ampicillin but less stable nanoparticles when amikacin was used.^90^

Salarian et al. showed the synergistic antibacterial properties of cephalexin NPs combined with AgNPs against *S. aureus*.^91^ Rogowska et al. assessed the antibacterial activity of biologically and chemically synthesized AgNPs functionalized with ampicillin against bacterial strains. The biosynthesized ampicillin–AgNPs showed a synergistic effect against *E. coli*, *K. pneumoniae*, and *P. aeruginosa*, whereas chemically synthesized AgNPs only exhibited synergism against *K. pneumoniae* and *P. aeruginosa*. These results may be related to the differences in the stability of the nanoparticles when conjugated with ampicillin, since biologically synthesized AgNPs were more stable than chemically generated AgNPs (zeta potentials of −18.50 ± 0.99 and −11 ± 0.20 mV, respectively).^93^ In another work, the authors combined chemically synthesized AgNPs with vancomycin and amikacin, demonstrating a synergistic antimicrobial effect against *S. aureus* and *E. coli*. Here, the characterization of the nanoparticles with and without antibiotics was performed by comparing UV–vis spectroscopy to the corresponding surface plasmon resonance (SPR). The AgNPs alone showed a SPR at 431 nm, and a blue shift was observed by adding vancomycin (2 nm) and amikacin (15 nm). This effect can be attributed to the charge transfer between the antibiotics and PVP-coated AgNPs. In addition, in the case of amikacin, it can also be due to the electronic transitions between different orbitals with the possibility of a nucleophilic substitution reaction between a lone pair of electrons in the oxygen atom of PVP and the hydrogen atom of the amikacin amine group. Furthermore, electronic transitions may occur between the bonding or nonbonding orbital and the antibonding orbital.^95^ In a similar work, Kaur et al. showed synergistic antimicrobial results combining citrate-capped AgNPs with vancomycin against *S. aureus* and *E. coli*. In this case, a red shift in SPR was observed in the UV–vis spectra after the addition of vancomycin. The PdI and zeta potential showed an inferior PdI and superior stability of vancomycin-conjugated AgNPs. X-ray diffraction (XRD) analysis studies showed that the crystalline nature of the AgNPs after antibiotic functionalization remains intact.^99^

McShan et al. suggested that the combination of the ineffective tetracycline or neomycin with AgNPs against *S. typhimurium* inhibits the growth of this bacterium. Nevertheless, the same was not verified for penicillin.^97^ Wang et al. showed the enhanced antibacterial activities of AgNPs against three bacterial strains: *S. aureus*, *E. coli*, and gentamicin-resistant *E. coli*, indicating that gentamicin considerably promotes the dissolution of PVP-AgNPs, which not only increases the concentration of silver ions but also assists in the attachment of PVP-AgNPs onto the surface of bacteria by mitigating the negative charge of the NPs.^98^

Panáček et al. performed a systematic study to quantify the synergistic effects of antibiotics with different modes of action and different chemical structures combined with AgNPs against *E. coli*, *P. aeruginosa*, and *S. aureus*. The researchers did not notice any trends for synergistic effects of antibiotics with different modes of action, which indicates a nonspecific synergistic effect. Notably, a low amount of AgNPs was required for effective antibacterial action.^101^ Deng et al. combined citrate stabilized AgNPs with several antibiotics against nonresistant and MDR *S. typhimurium* and observed several synergistic combinations. In this work, a particular study was performed by Raman spectroscopy to verify the interaction between AgNPs and antibiotic molecules. The authors found that ampicillin and penicillin did not replace the stabilizing molecules used during synthesis. On the contrary, the antibiotics enoxacin, kanamycin, neomycin, and tetracycline strongly interact with AgNPs, replacing the surface citrate molecules and forming antibiotic–AgNP complexes. These antibiotics readily caused the agglomeration of AgNPs.^103^

Just one work was found combining AuNPs and antibiotics using method A. The work was developed by Tom et al.; the authors used ciprofloxacin to protect the AuNPs, but no antimicrobial analyses were performed.^104^

Bhande et al. demonstrated the potential of ZnONPs to act as β-lactam antibiotics.^107^ Rath et al. combined ZnONPs and cefazolin, showing a higher antibacterial activity.^108^ Abo-Shama et al. tested the synergistic effect of antibiotics (azithromycin, oxacillin, cefotaxime, cefuroxime, fosfomycin, and oxytetracycline) against *E. coli*. The results showed a significant increase in the presence of ZnONPs when compared to the antibiotic alone. They also tested the synergistic effect of antibiotics (azithromycin, cefotaxime, cefuroxime, fosfomycin, chloramphenicol, and oxytetracycline) against *S. aureus*, which also showed significantly increased antimicrobial effects in the presence of ZnONPs.^127^ Eleftheriadou et al. studied the potential of polyol-coated CuONPs and ZnONPs combined with meropenem and ciprofloxacin as efflux pump inhibitors against MDR *P. aeruginosa*. The results demonstrated that all tested NPs act synergistically in the presence of the antibiotics, depending on the concentration.^105^ MadhumitaGhosh et al. showed synergistic results combining ZnO NPs with erythromycin against *P. aeruginosa*.^140^ All these works confirm the synergistic effect of ZnONPs with different classes of antibiotics.

Cu and CuONPs have revealed synergistic effects when combined with gentamicin, doxycycline, and amoxicillin/clavulamic acid *againstE. coli*, *P. aeruginosa*, *B. cereus*, *P. mirabilis*, and *S. aureus*.^106,131,132^

Vernaya et al. showed the efficacy of FeNPs as promising precursors of targeted drug delivery systems. In this work, gentamycin was combined with chemically synthesized FeNPs.^106^

Only three works were found to display the development of bimetallic NPs and posterior conjugation with antibiotics, in particular Ag–Au, Ag–Pt, and Cu–Zn nanoparticles. Fakhri et al. tested the synergistic antimicrobial activity of doxycycline-conjugated bimetallic Ag–AuNPs against *P. aeruginosa*, *E. coli*, *S. aureus*, and *M. luteus*, showing promising results for burn healing therapy.^110^ In more recent work, Cu–ZnNPs were, for the first time, tested by Eleftheriadou et al. The Cu–ZnNPs and meropenem combination resulted in an additive effect at 25 μg/mL and partially in a synergistic or additive effect at the two highest concentrations tested (50 and 100 μg/mL) against *P. aeruginosa*.^105^ Lastly, Ranpariya et al. studied the bimetallic Ag–PtNPs combined with streptomycin, rifampicin, chloramphenicol, novobiocin, and ampicillin against *E. coli*, *P. aeruginosa*, and *S. aureus*. The inhibitory activity of Ag–PtNPs was more efficient against all pathogens than that of individual AgNPs or PtNPs. In the antimicrobial synergy tests, the activity of rifampicin and novobiocin combined with Ag–PtNPs showed a significant result against *S. aureus*.^122^ The bimetallic MNP-conjugated antibiotics showed interesting antimicrobial properties and may be a promising tool for developing novel agents.

#### 5.1.2 Method B

In the case of method B, the MNP synthesis was performed mostly using chemical methods and in the presence of antibiotics (Section 4, Scheme 2). In this approach, the antibiotics may or not act as a reducing agent, but a stronger reducing agent is always applied (Table 3).

**Table 3:** Synergic Studies between MNPs and Antibiotics Obtained by the MNP Synthesis in the Presence of Antibiotics: Method B

MNPs, size (nm)	MP synthesis	reducing agent	stabilizing agent	combined antibiotics	bacterial strains	antimicrobial results	ref
Au, >14.0	chemical	sodium borohydride and ampicillin	ampicillin	ampicillin	*E. coli*, *M. luteus*, and *S. aureus*	MIC: *E. coli*, *M. luteus*, and *S. aureus*, ampicillin (slight synergism)	(141)
Au, >14.0	chemical	sodium borohydride and streptomycin	streptomycin	streptomycin	*E. coli*, *M. luteus*, and *S. aureus*	MIC: *E. coli*, *M. luteus*, and *S. aureus*, streptomycin (significant synergism)	(141)
Au, >14.0	chemical	sodium borohydride and kanamycin	kanamycin	kanamycin	*E. coli*, *M. luteus*, and *S. aureus*	MIC: *E. coli*, *M. luteus*, and *S. aureus*, kanamycin (significant synergism)	(141)
Ag, 270.0	chemical	ammonia, polydopamine, and vancomycin	polydopamine	vancomycin	*E. coli* and *S. aureus*	colony counting: synergism	(142)
Ag, 5.0–33.0	chemical	trisodium citrate and ampicillin or penicillin or vancomycin	trisodium citrate and ampicillin or penicillin or vancomycin	ampicillin, penicillin, and vancomycin	*E. coli*, *K. pneumonia*, and *S. aureus*	FIC: *E. coli*, ampicillin and penicillin; *S. aureus*, penicillin and vancomycin; *K. pneumonia*, vancomycin	(143)
Ag, 18.5	chemical	formic acid and sulfanilamide	PVA and chitosan	sulfanilamide	*E. coli*, *P. aeruginosa*, and *S. aureus*	ZoI: *E. coli*, *P. aeruginosa*, and *S. aureus*, sulfanilamide	(144)
Ag, 5.0–33.0	biosynthesis and chemical	*Pyrenacantha grandiflora Baill* extract and ampicillin or penicillin or vancomycin	*Pyrenacantha grandiflora Baill* extract and ampicillin or penicillin or vancomycin	ampicillin, penicillin, and vancomycin	*E. coli*, *K. pneumonia*, and *S. aureus*	FIC: *E. coli*, vancomycin and penicillin; *K. pneumonia*, penicillin and ampicillin	(143)

The antibiotics were conjugated with AgNPs or AuNPs by reducing the corresponding metal salts with sodium borohydride, trisodium citrate, ammonia, formic acid, or plant extracts. The first demonstration of method B was performed by Saha et al. The authors tested the synthesis of AuNPs using antibiotics (ampicillin, streptomycin, and kanamycin) as reducing agents. However, the results showed that the used antibiotics did not exhibit sufficient reducing power to perform the redox reaction. The reaction time to obtain AuNPs took 4 h when ampicillin was used and 24 h with streptomycin or kanamycin. In addition to the extended reaction time, the obtained antibiotic-conjugated AuNPs showed high agglomeration and quickly precipitated, whereas the AuNPs produced using the combined reducing properties of both sodium borohydride and the antibiotics displayed superior stability. The SPR of antibiotic-conjugated AuNPs appeared in a more bluish region of UV–vis spectra, suggesting larger NPs as confirmed by TEM. Scanning electron microscopy (SEM) images showed different shapes of AuNPs using distinct antibiotics: cubic structure with ampicillin, rectangular rod-shaped with streptomycin, and star-like structures with kanamycin. The AuNP-conjugated antibiotics displayed superior bactericidal activity. The MIC values of the conjugates were determined against *E. coli*, *M. luteus*, and *S. aureus*. Among them, streptomycin and kanamycin conjugates showed a significant reduction in MIC values. In contrast, AuNP–ampicillin showed a slight decrease in the MIC value when compared to its free form.^141^ Ganesh et al. prepared AgNPs decorated with chitosan and poly(vinyl alcohol) (PVA) using formic acid as a reducing agent. The AgNPs were prepared in one solution containing sulphanilamide. The main objective of this experiment was to produce nanofibers with incorporated AgNPs. Thus, PVA was introduced to allow the electrospinning of the mixture. The antimicrobial tests and *in vivo* wound healing evaluation demonstrated superior and synergistic activity due to the combination of AgNPs and sulphanilamide.^144^ Ma et al. developed an efficient nanohybrid using vancomycin-carrying polydopamine with AgNPs. Zeta potential, XRD, and X-ray photoelectron spectroscopy (XPS) analysis proved the successful AgNP modification. In the XPS analysis, the survey spectra showed the presence of two specific peaks centered at 368.0 and 374.0 eV assigned to Ag 3d5/2 and Ag 3d3/2 electrons of Ag^0^, respectively. It proves the assembly of AgNPs (Ag^0^) with polydopamine. The synthesized hybrid showed synergistic antibacterial performance against both *S. aureus* and *E. coli* strains. The development of this hybrid allowed the drug dosage to be reduced, decreasing the chance to develop drug resistance.^142^ In another work, *Pyrenacantha grandiflora* tuber extracts were combined with ampicillin, penicillin, vancomycin, and AgNPs. The antimicrobial activity was assessed against *E. coli*, *S. aureus*, and *K. pneumoniae*. The overall results demonstrated that the conjugation of antibiotics with AgNPs are an effective option to improve the activity of antibiotics that have become less effective.^143^

#### 5.1.3 Method C

In the literature, few methods were found using method C (Section 4, Scheme 2). In this method, MNPs were synthesized, and surfaces were functionalized and subsequently combined with antibiotics (Table 4).

**Table 4:** Synergic Studies between MNPs and Antibiotics Obtained by MNP Synthesis, Subsequent MNP Functionalization, and a Combination of Antibiotics: Method C

MNPs, size (nm)	MP synthesis	reducing agent	stabilizing agent	combined antibiotics	MNP surface functionalization method	bacterial strains	antimicrobial results	ref
Ag, 4.0	chemical	sodium borohydride	trisodium citrate dihydrate	ampicillin	thioether group from ampicillin	*P. aeruginosa*, *E. aerogenes*, *E. coli*, *V. cholerae*, and methicillin-resistant *S. aureus* and *E. coli*	MBC: *P. aeruginosa*, *E. aerogenes*, *E. coli*, *V. cholerae*, and methicillin-resistant *S. aureus* and *E. coli*, ampicillin	(145)
Au, 4.0	chemical	sodium borohydride	trisodium citrate dihydrate	ampicillin	thioether group from ampicillin	*P. aeruginosa*, *E. aerogenes*, *E. coli*, *V. cholerae*, and methicillin-resistant *S. aureus* and *E. coli*	MBC: *P. aeruginosa*, *E. aerogenes*, *E. coli*, *V. cholerae,* and methicillin-resistant *S. aureus* and *E. coli*, ampicillin	(145)
Au, 4.0–5.0	chemical	sodium borohydride	n.a.	vancomycin	bis(vancomycin) cystamide	*E. faecium*, *E. faecalis*, *E. faecalis* resistant, and *E. coli*	MIC: *E. faecium*, *E. faecalis* resistant, and *E. coli*, vancomycin	(146)
Ag, 12.0	chemical	ethylene glycol	PVP	ampicillin	treatment with TEOS, reaction with APTES, and ampicillin addition	susceptible and ampicillin-resistant *E. coli*	inhibition (plate counting): the synergism was not assessed; the AgNP–ampicillin conjugates showed a good antimicrobial effect for both strains with low cytotoxicity	(147)
Ag, 16.0	chemical	sodium borohydride	mercaptoacetic acid	norvancomycin	EDAC activated the reaction between the carboxyl of mercaptoacetic acid and the amide group of norvancomycin	*E. coli*	OD and inhibition (plate counting): *E. coli*, norvancomycin	(148)
Au, 2.0	chemical	sodium borohydride in the presence of 1-pentanethiol	thiol groups	ciprofloxacin and levofloxacin	pentane-thiol capped AuNPs mixed with antibiotic	MDR *E. coli*	FIC: MDR *E. coli*, ciprofloxacin and levofloxacin	(149)
Au, 2.0	chemical	sodium borohydride in the presence of 1-pentanethiol	thiol groups	ciprofloxacin and levofloxacin	synthesis of AuNPs, functionalization with pentane-thiol, mixture with antibiotics	MDR *E. coli*	FIC: MDR *E. coli*, ciprofloxacin and levofloxacin	(149)
ZnO, 20.0–24.0	chemical	sodium hydroxide	starch	ciprofloxacin	amine functionalization of nanoparticles using 3-ethyldimethylaminopropyl carbodiimide/*N*-hydroxysuccinimide (EDC/NHS)	*B. subtilis*, *Streptococcus* spp., and *E. coli*	ZoI: all combinations	(150)
Au, 15.0	biogenic, fungal	biomass of *Trichoderma viride*	biomolecules from biomass	vancomycin	ionic interaction between the amino group of vancomycin and negative surface charge of AuNPs	*E. coli*, *S. aureus*, and vancomycin-resistant *S. aureus* strains	MIC: *E. coli*, *S. aureus*, and vancomycin-resistant *S. aureus*, vancomycin	(151)

**Figure 1:**
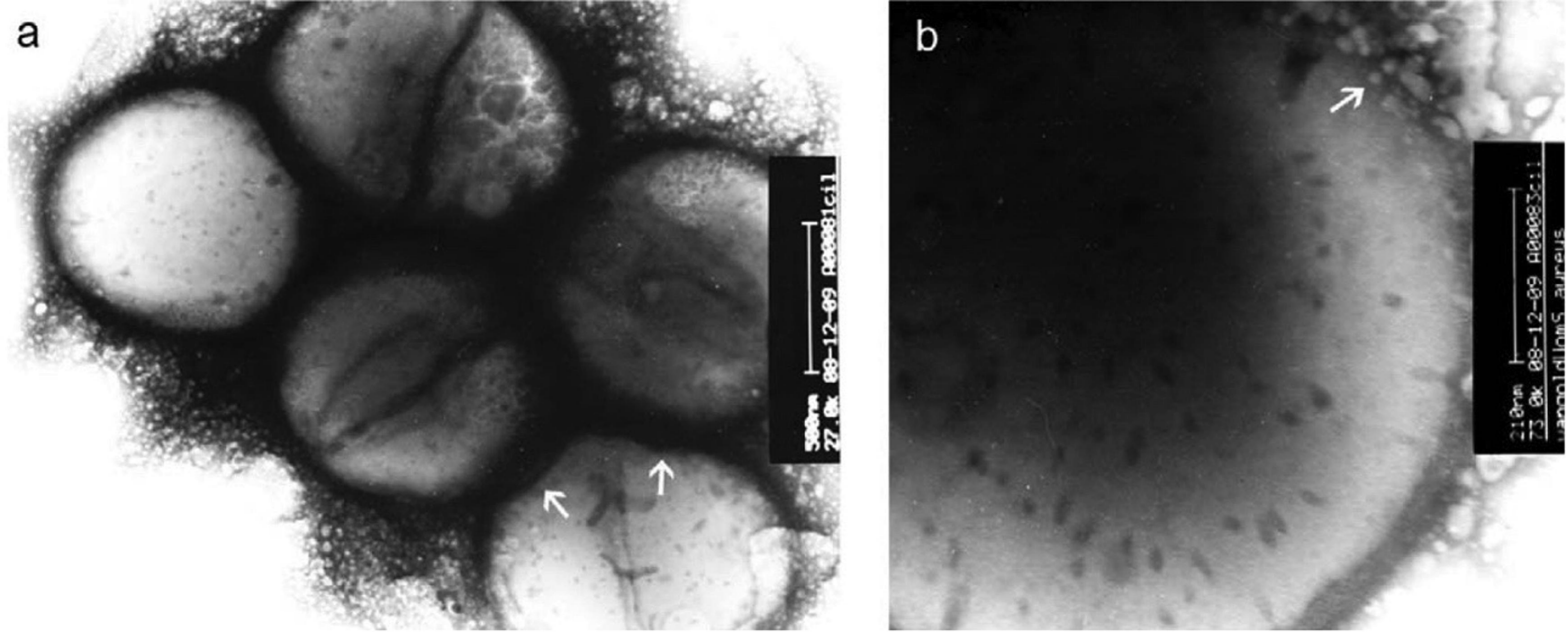
(a) TEM images of vancomycin-resistant *S. aureus* cells treated with vancomycin–AuNPs conjugates. (b) Expanded view of an individual cell membrane of a vancomycin-resistant *S. aureus* bacterial cell treated with vancomycin–AuNP conjugates. Reproduced with permission from ref (151). Copyright 2011 Elsevier.

Chemical and biogenic methods may be used for MNP synthesis when following method C. The functionalization of the MNP surface is always a posterior step. AuNPs, AgNPs, and ZnONPs were the only reported MNPs according to this method. AuNPs are the most frequent, probably due to their easy functionalization with thiol groups. Brown et al. synthesized AgNPs and AuNPs stabilized in citrate, and then, the NPs were functionalized with ampicillin. The thioether moiety present in the structure of ampicillin was used to attach the antibiotic to the AgNPs and AuNPs. Both nanoparticles functionalized with ampicillin exhibited active broad-spectrum bactericides against *Gram-negative* and *Gram-positive* bacteria. The conjugates are becoming potent bactericidal agents with unique properties that disrupt antibiotic resistance mechanisms of MDR strains.^145^ de Oliveira et al. functionalized chemical synthesized PVP-AgNPs with ampicillin using a multistep method. First, a core–shell of silica in the AgNPs was prepared by a reaction with tetraethyl orthosilicate hydrolysis, forming the corresponding AgSiO_2_NPs. Next, AgSiO_2_NPs were coated with a thin silica/amine layer. In this step, an ethanol solution containing ammonia and AgSiO_2_NPs reacted with tetraethyl orthosilicate (TEOS). The next step consisted of the reaction of the NPs with 3-aminopropyltriethoxysilane (APTES). Finally, the NP dispersion was mixed with an ampicillin solution in an acidic medium using 2-(*N*-morpholino) ethanosulfonic acid.^147^ Gu et al. demonstrated one synthetic route for formulating vancomycin–AuNPs with enhanced antibacterial activity. AuNPs reacted with bis(vancomycin) cystamide to form Au–S bonds that link vancomycin to AuNPs.^146^ Mohammed Fayaz et al. prepared vancomycin bound biogenic AuNPs by stirring the AuNP dispersion and vancomycin for 24 h. The formulation was stable for at least 90 days. The vancomycin–AuNPs showed significant antibacterial activity against *E. coli* and *S. aureus* susceptible and vancomycin-resistant strains. The vancomycin–AuNPs are shown to bind to transpeptidase instead of terminal peptides of the glycopeptide precursors on the cell surface of resistant *S. aureus*, inducing the lysis of the cell wall (Figure 1).^151^ The antibiotic-functionalized NPs were further characterized, and the antimicrobial activity was evaluated.

Gupta et al. performed a slightly different approach, where prior to the combination of antibiotics, the AuNPs were functionalized with thiol ligands. The chemical reduction of the gold salt was performed in the presence of 1-pentanethiol. The thiol protected AuNPs were revealed to be highly stable due to the strong thiol–gold interaction. Next, the ligand functionalization of the AuNP core with hydrophobic ligands was done using a place-exchange method. The influence of the ligands onto the NP surface and their combination with fluoroquinolone antibiotics were studied. This demonstrated the synergistic antimicrobial therapy and decreased antibiotic dosage using hydrophobically functionalized AuNPs and fluoroquinolone antibiotics to fight against MDR bacterial strains. This strategy shows the potential of using AuNPs to “revive” ineffective antibiotics due to the development of resistance by bacteria.^149^ Wei et al. developed norvancomycin-capped AgNPs with notable antibacterial effects against *E. coli*. The antibiotic was grafted to the terminal carboxyl of the mercaptoacetic acid in the AgNPs in the presence of *N*-(3-(dimethylamino)propyl)-*N*′-ethylcarbodiimide hydrochloride (EDAC).^148^ A report depicted this conjugation method with the antibiotic ciprofloxacin and amine-functionalized ZnONPs. The amine functionalization was obtained by a chemical process using 3-ethyldimethylaminopropyl carbodiimide (EDC) and *N*-hydroxysuccinimide (NHS). In regard to antibacterial activity, synergistic effects were observed when ZnONPs were used in conjugation with antibiotics against all tested bacterial strains.^150^

#### 5.1.4 Method D

In the last approach, the synergistic effect was achieved by synthesizing MNPs using antibiotics as reducing agents, converging two steps in one (method D, Table 5).

**Table 5:** Synergic Studies between MNPs and Antibiotics Obtained by MNP Synthesis Using Antibiotics as Reducing Agents: Method D

MNPs, size (nm)	MP synthesis	reducing agent/antibiotic	bacterial strains	antimicrobial results	ref
Ag, 10.8 and 33.9	chemical	ampicillin	*S. aureus*, *S. pyogenes*, *P. aeruginosa*, *E. coli*, *S. typhimurium*, *Klebsiella*, *E. cloacae*, and *S. pneumoniae*	MIC: the synergistic effect was not evaluated; the NPs without functionalization were not obtained nor was the MIC for the antibiotics alone	(83)
Ag, 44.1	chemical	ampicillin	*E. coli*, *S. aureus*, ampicillin-resistant *E. coli* and *S. aureus*, multidrug-resistant *P. aeruginosa*, and *K. pneumonia*	ZoI: *E. coli*, *S. aureus*, resistant *E. coli* and *S. aureus*, resistant *P. aeruginosa*, and *K. pneumonia*, ampicillin	(152)
Au, 18.7	chemical	ampicillin	*S. aureus*, *S. pyogenes*, *P. aeruginosa*, *E. coli*, *S. typhimurium*, *Klebsiella*, *E. cloacae* and, *S. pneumoniae*	MIC: the synergistic effect was not evaluated; the NPs without functionalization were not obtained nor was the MIC for the antibiotics alone	(83)
Au, 52.0 to 23.0	chemical	cefaclor	*S. aureus* and *E. coli*	MIC and inhibition (plate counting): *E. coli*, cefaclor	(153)
CuS, 15.0	chemical	vancomycin	*E. faecium* and *E. faecalis*	synergism not evaluated, enhanced bactericidal effect in antimicrobial phototherapy	(154)

Hur et al. described the functionalization of AuNPs and AgNPs with ampicillin, which acted as a reducing agent to convert gold and silver salts in the respective nanoparticles, minimizing the use of chemical agents during the synthetic route. Curiously, the newly prepared NPs showed excellent antibacterial activity against *S. pyogenes*.^83^ Khatoon et al. published the synthesis of AgNPs using ampicillin as a reducing agent. The PdI was found to be 0.32 and the zeta potential, +33.42 mV, which indicate the long-term stability of ampicillin–AgNP suspension. The ampicillin content on the conjugates was evaluated by thermogravimetric analysis (TGA), where 2.1% to 4.3% of weight loss between 30 and 200 °C was attributed to ampicillin on the surface of the AgNPs. The antibacterial potential of ampicillin–AgNPs was studied against sensitive and drug-resistant bacteria. MIC values of ampicillin–AgNPs against six different bacterial strains were in the range of 3–28 μg mL^–1^, which is much lower than the MIC of ampicillin alone (12–720 μg mL^–1^) and chemically synthesized AgNPs (280–640 μg mL^–1^). The results also indicated that bacterial strains do not show any resistance to ampicillin–AgNPs even after 15 successive cycles.^152^ Rai et al. reported a one-pot synthesis of spherical AuNPs capped with cefaclor without the use of other chemicals. The primary amine group in the cefaclor molecule acted as both the reducing and capping agent for AuNP synthesis, leaving the β-lactam ring of cefaclor available for its antimicrobial action. TEM images and DLS analysis showed the size of the AuNPs ranged from 52 ± 1.5 to 23 ± 2 nm with increasing temperature from 20 to 60 °C of the reaction solution. A red shift of 7 nm was observed in the SPR band centered at 528 nm when cefaclor was used, suggesting a small population of aggregated gold nanostructures in solution as also observed using TEM analysis. The TGA analysis showed three distinct weight losses at three different temperature regions indicating that cefaclor interacts with NPs by physical adsorption (weight loss in the lower temperature region) via rearrangement of bound cefaclor molecules (276 to 470 °C region) and by covalent bonds (515 to 660 °C). FTIR also confirmed the presence of cefaclor with the characteristic β-lactam ring vibrations at 1418, 1395, and 1357 cm^–1^. The antimicrobial activity tests showed the growth inhibition of *E. coli*.^153^ The covalently bonded method is preferred to simple physical adsorption due to the uncontrollable release of the drug from the nanoparticles of the latter. However, few reports were found using this strategy.^152^ Thus, novel experiments are needed to develop metal nanostructures combined with commercial antimicrobial agents to improve their bonding.

### 5.2 Antifungals

**Chart 4:**
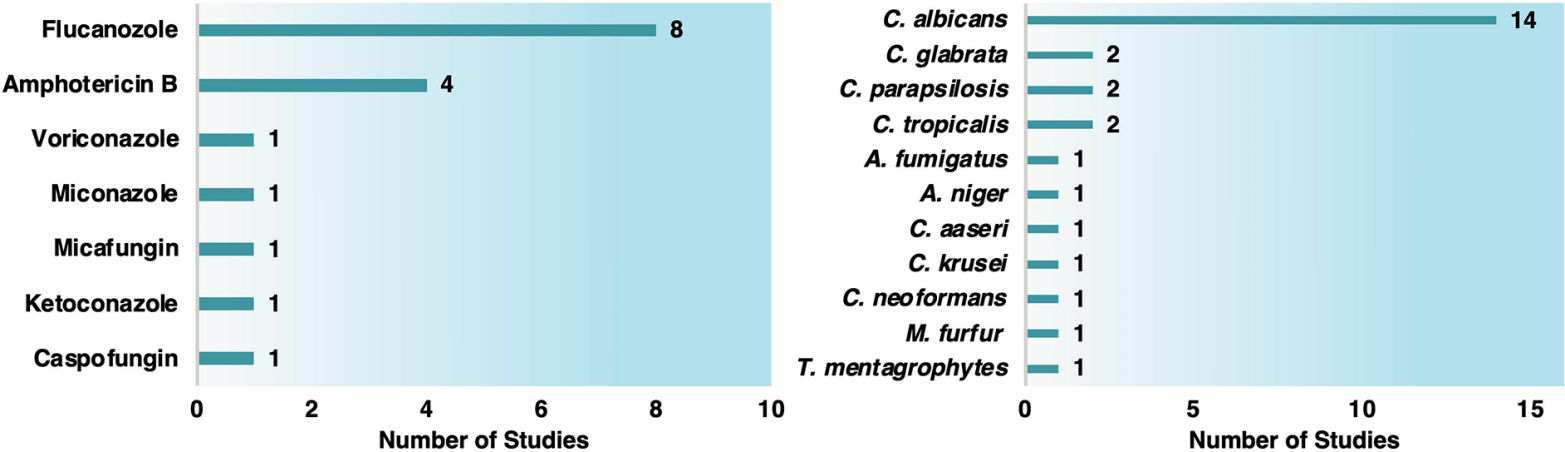
Number of Studies Combining MNPs and Antifungal Agents by Drugs and Fungal Strains

Invasive fungal infections have steadily increased over the past decades, and the mortality rates remains very high, especially in immunocompromised patients. It is estimated that more than 2 million people die annually of invasive fungal infections. This imperatively urges the identification of new classes of treatment options. For immunocompromised patients, the mortality is still very high for infections caused by *Candida albicans* (20–40%), *Candida neoformans* (20–70%), and *Aspergillus fumigatus* (50–90%), reaching a death rate of about 50%.^155,156^ Recently, severe COVID-19 disease was correlated to an increase in pro-inflammatory markers, consequently increasing susceptibility to bacterial and fungal infections such as mucormycosis, candidiasis (*Candida auris*), SARS-CoV-2-associated pulmonary aspergillosis, Pneumocystis pneumonia, and Cryptococcal disease.^157^ The antifungal agents available in current clinical treatments are very limited compared to antibacterial agents. They are not effective or safe due to the development of resistance and host toxicity.^158^ Only five classes of antifungal drugs exist and include the azoles, polyenes, echinocandins, allylamines, and antimetabolites. The available antifungal agents still exhibit several limitations in managing fungal infections. The emergence of drug-resistant fungi and the severe nephrotoxicity of some antifungals make the problem more and more serious.^159,160^ The development of new antifungal agents is not matching the frequency of antifungal-resistance appearance. The development of conjugate commercial antifungals has been attempted, but the trials have shown weak and sometimes contradictory results. Thus, more experiments and more specific recommendations for clinicians are needed.^161^ In the last years, few works have been published considering antifungal agents and MNPs (13 studies). Most of them used AgNPs and azole or polyene drugs. Fluconazole and amphotericin B were the most prevalent antifungal agents. The efficacy of antifungal drugs and MNP combinations against *Candida albicans* (14 studies) was the most studied (Chart 4).

#### 5.2.1 Method A

Method A was the most used strategy to obtain the dispersions with the conjugates. The MNPs were obtained by chemical, electrochemical, or biological methods, and the sizes varied from 1 to 68.7 nm in isolated MNPs or were 80 nm when stabilized onto zeolites (Table 6).

**Table 6:** Synergic Studies between MNPs and Antifungals Obtained by the MNPs’ Synthesis and Their Posterior Combination with Antifungals: Method A

MNPs, size (nm)	MP synthesis	reducing agent	stabilizing agent	combined antifungal agent	fungi strains	test method/synergic results	ref
Ag, 1.0–50.0	biogenic, fungal	fungus *Aspergillus oryzae*	biomass from *Aspergillus oryzae*	fluconazole	*C. albicans*, *C. glabrata*, *C. parapsilosis*, *C. krusie*, *C. tropicalis*, and *C. albicans*	MIC and FIC: *C. albicans*, *C. glabrata*, *C. parapsilosis*, *C. krusie*, *C. tropicalis*, and *C. albicans*	(162)
Ag, 9.8	biogenic, bacteria	supernatant of *Delftia* sp. strain	supernatant of *Delftia* sp.	miconazole	*C. albicans*, *C. parapsilosis*, *C. aaseri*, and *C. glabrata*	MIC: *C. albicans*, *C. parapsilosis*, *C. aaseri*, and *C. glabrata*, miconazole	(163)
Ag, 34.4–68.7	biogenic, plant	phytochemicals from *Polyalthia longifolia*	n.a.	amphotericin B	*C. albicans*	FIC: synergism	(164)
Ag, 24.1	electrochemical	n.a.	PVP	fluconazole and voriconazole	*C. albicans* clinical isolates	FIC: all combinations	(165)
Ag-zeolite, 80.0	chemical	trisodium citrate	trisodium citrate	fluconazole, caspofungin, and micafungin	*C. albicans*	OD: *C. albicans*, caspofungin, and micafungin	(166)
CuO, 6.5	chemical	sodium hydroxide	n.a.	fluconazole	*C. albicans*	FIC: no synergism, just additive effect	(167)
ZnO, 35.0	chemical	sodium hydroxide	n.a.	fluconazole	*C. albicans* isolates	ZoI and MIC: synergism	(168)
CuO, 50.0	n.a.	n.a.	n.a.	fluconazole	*C. albicans*	FIC: no synergism, just an additive effect	(167)
Ag, 8–12	commercial	n.a.	PVP	fluconazole	*C. albicans* SC5314 and clinical isolates	FIC: *C. albicans* clinical isolates	(169)
ZnO (pure and Mn, Cu, Co, or Fe doped), 20.0	commercial	n.a.	n.a.	flucanozole and amphotericin B	*A. fumigatus*, *C. albicans*, *C. neoformans*, and *T. mentagrophytes*	FIC: *T. mentagrophytes*, amphotericin and ZnO doped	(170)

**Figure 2:**
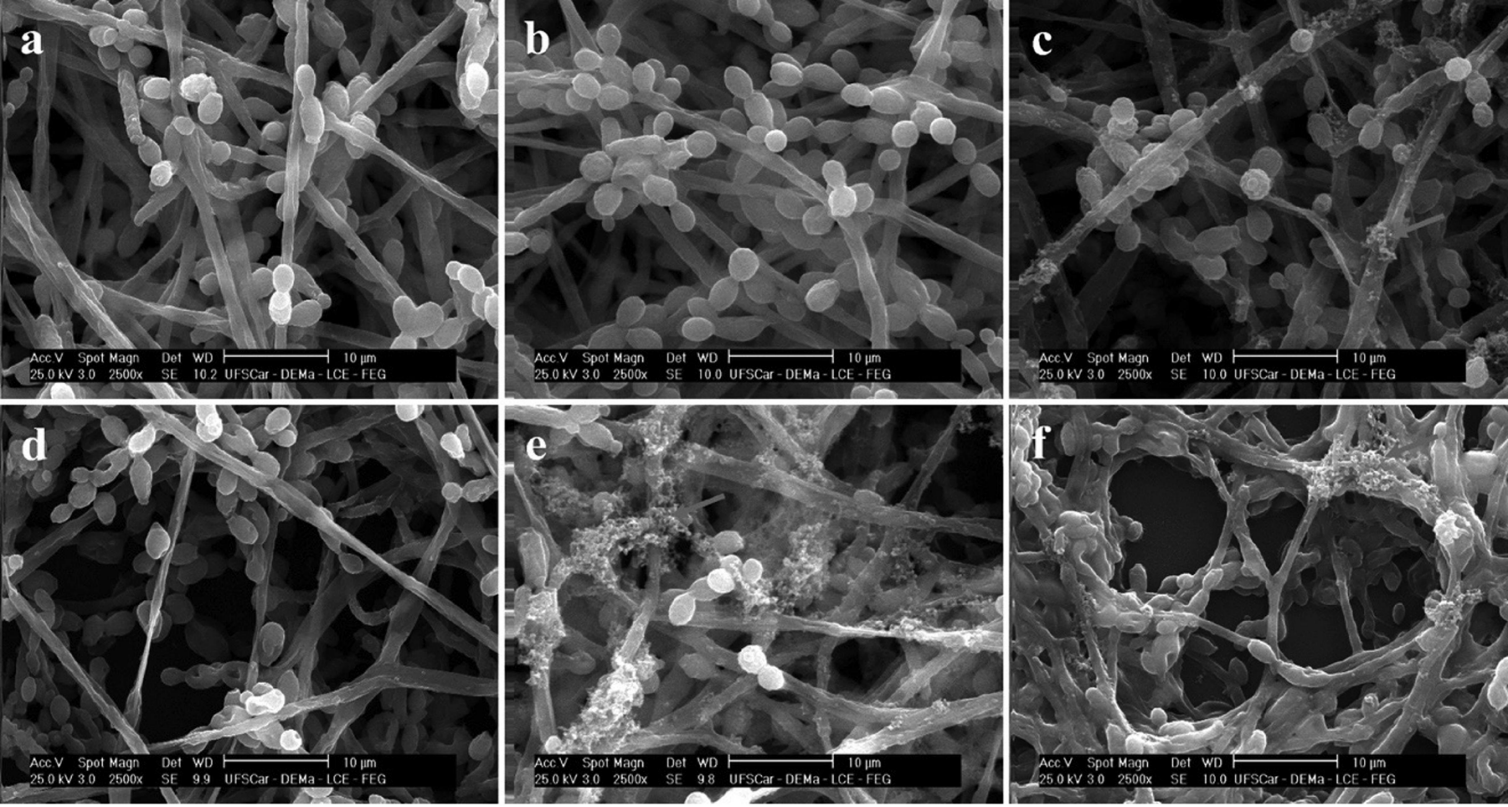
SEM images of dual-species biofilms of *C. albicans* and *C. glabrata* species (a) untreated and treated with (b) 110 μg·mL^–1^ Fe_3_O_4_NPs, (c) 110 μg·mL^–1^ chitosan, (d) 78 μg·mL^–1^ miconazole, and miconazole–Fe_3_O_4_NPs conjugates at (e) 31.2 μg·mL^–1^ and (f) 78 μg·mL^–1^ at a magnification of 2500×. Reproduced with permission from ref (172). Copyright 2020 Elsevier.

Kumar and Poornachandra tested the efficacy of AgNPs combined with miconazole against *Candida* strains, obtaining significant increased fungicidal activity. TEM and FTIR confirmed the NP conjugation with miconazole. TEM images showed monodispersed nanoparticles with an average size of 9.8 and 23.9 nm for AgNPs and miconazole–AgNPs, respectively. The FTIR spectra demonstrated similar functional groups of miconazole in the conjugates, indicating the successful conjugation of the miconazole drug to the AgNPs.^163^ Sun et al. studied the potential synergy between AgNPs and azole antifungals against drug-resistant *C. albicans*. Any inhibition of the drug-resistant *C. albicans* was observed using fluconazole or voriconazole alone. AgNPs alone had only moderate killing ability. However, the combined treatment was effective against the drug-resistant *C. albicans*.^165^ Li et al. referred to the combination of sublethal AgNPs and echinocandin drugs with potent synergistic effects against *C. albicans*.^166^ Weitz et al. tested the combination of CuONPs and fluconazole as a potential treatment against the pathogenic *C. albicans*. However, the results just showed additive effects.^167^ Sharma et al. studied the antimicrobial activity of doped ZnONPs with manganese (Mn), copper (Cu), cobalt (Co), or iron (Fe), where additive and synergistic effects were found depending on the dopant. ZnO doped with Mn (1% and 10%), Co (1% and 10%), or Cu (10%) showed antifungal synergistic results, and the other combinations just displayed additive effects.^170^ MNP conjugates also presented a synergistic effect against biofilms.^171^ SEM images from miconazole–Fe_3_O_4_NPs against dual-species biofilms of *C. albicans* and *C. glabrata* revealed ruptures in the biofilms, generating less dense structures (Figure 2e,f) than the untreated biofilm and also than the biofilms only treated with Fe_3_O_4_NPs, chitosan, or miconazole (Figure 2).^172^

#### 5.2.2 Method B

No works using method B for the conjugation of MNPs with antifungal agents were found in the literature.

#### 5.2.3 Method C

Just two publications were found using method C. After MNP synthesis, the MNPs were functionalized and combined with antifungals. In these works, the AgNPs were functionalized using *N*-[3-(trimethoxysilyl) propyl] diethylenetriamine (ATS) or 1-(3-(dimethylamino)propyl) 3-ethylcarbodiimidehydrochloride (EDC) and hydroxysuccinimide (NHS). Posteriorly, the functionalized NPs were mixed with ketoconazole and amphoteric B, respectively (Table 7).

**Table 7:** Synergic Studies between MNPs and Antifungals Obtained by MNP Synthesis, Subsequent MNPs Functionalization, and a Combination of Antifungals: Method C

MNPs, size (nm)	MP synthesis	reducing agent	stabilizing agent	combined antifungal agent	MNP surface functionalization method	fungi strains	antimicrobial results	ref
Ag, 15.0	chemical	[*N*-[3-(trimethoxysilyl) propyl] diethylenetriamine] (ATS)	ATS	ketoconazole	ATS under a nitrogen atmosphere mixed with silver nitrate for 4 h and mixed with antifungal	*Malassezia furfur* clinical isolate	FIC: *M. furfur*, ketaconazole	(173)
Ag, 15.0	biosynthesis	extract of *Maytenus royleanus*	plant extract and amphotericin B	amphotericin B	AgNPs in acetate buffer were mixed with EDC and NHS; then, the amphotericin B was added and kept under stirring for 4 h	*C. albicans* and *C. tropicalis*	ZoI: *C. albicans* and *C. tropicalis*, amphotericin B	(174)

AgNPs functionalized with ATS were mixed with ketoconazole; the conjugates were shown to be spherical in shape, and a stable dispersion was obtained (without any agglomeration). However, the interactions between the AgNPs and ketoconazole were not studied. The synergistic effect was observed in 17.08% of the isolates.^173^ Amphoteric B–AgNPs also showed a spherical shape and were provided by an ester linkage promoted by the EDC molecules and hydroxyl groups from biomolecules in the AgNP surface. The conjugation of amphotericin B and AgNPs was assessed by UV–visible spectroscopy. The SPR peak of AgNPs alone (424 nm) red-shifted toward a longer wavelength by 24 nm (448 nm), indicating the conjugation of amphotericin B to AgNPs, which was well supported by FTIR and TEM results. AgNPs alone revealed low to moderate antifungal activity (ZoI 4–8 ± 0.2 mm). However, the amphotericin B-conjugated AgNPs exhibited significant activity against *C. albicans* (ZoI 16 ± 1.4 mm) and *C. tropicalis* (ZoI 18 ± 1.5 mm).^174^

#### 5.2.4 Method D

Lastly, a particular case using method D, MNP synthesis using antifungals as reducing agents, was found (Table 8). Here, amphotericin B acted as a reducing and stabilizing/capping agent in the AgNP synthesis. The reaction occurred in an alkaline environment to prevent aggregation and promote AgNP formation. This approach produced monodisperse AgNPs with a size of 7 nm. Amphotericin B–AgNPs were shown to be particularly effective against the most pathogenic fungi responsible for severe mycotic infections.^175^

**Table 8:** Synergic Studies between MNPs and Antifungals Obtained by MNP Synthesis Using Antifungals as Reducing Agents: Method D

MNPs, size (nm)	MP synthesis	reducing agent/antifungal	fungi strains	antimicrobial results	ref
Ag, 7.0	chemical	amphotericin B	*A. niger*, *C. albicans*, and *F. culmorum*	ZoI: *A. niger* and *C. albicans*, amphotericin B	(175)

In summary, the combination of MNPs with antifungals can have additive and synergistic effects depending on the type of MNPs applied. A doping agent can further enhance the antifungal effect under certain conditions. Thus, when one considers the presented results, additional studies need to be performed to improve the knowledge and applicability in a broader range of pathogenic fungi.

### 5.3 Antivirals

Viral infections remain a major threat to global public health and have been responsible for alarming deaths. Viral infections can affect several tissues and organs, namely, the upper respiratory tract and lungs (e.g., coronaviruses, rhinoviruses, and influenza), the colon (e.g., rotavirus), the liver (e.g., hepatitis B virus (HBV)), the spinal cord (e.g., poliovirus), vascular endothelial cells (e.g., ebola), leukocytes (e.g., human immunodeficiency virus (HIV) and ebola), skin (e.g., herpes viruses and papillomaviruses), and neural cells (e.g., enteroviruses).^176−180^ During human history, several virus outbreaks have occurred, causing millions of deaths worldwide.^181^ Also, the current COVID-19 pandemic emerged at the end of 2019, and its health and economic impact continue to represent an exponential hurdle for the entire world.^182^ The strategies for antiviral drugs are focused on two different approaches: targeting the viruses themselves or the host cells. Antiviral drugs that directly target the viruses include the inhibitors of virus attachment, virus entry inhibitors, uncoating inhibitors, polymerase inhibitors, protease inhibitors, inhibitors of nucleoside and nucleotide reverse transcriptase, and the inhibitors of integrase. The inhibitors of protease (ritonavir, atazanavir, and darunavir), viral DNA polymerase (acyclovir, tenofovir, valganciclovir, and valacyclovir), and integrase (raltegravir) are listed among the Top 200 Drugs by sales during the 2010s.^183^ Another antiviral agent class is the neuraminidase inhibitors (oseltamivir, zanamivir, and peramivir) broadly used against influenza. The adamantanes (amantadine) act by blocking the ion channel of the influenza virus, but it is rarely used due to the high resistance of the circulating strains.^184,185^ Thus, the approved drugs that present an inhibitory spectrum against nine human infectious diseases can be recapitulated as follows: human immunodeficiency virus (HIV), human cytomegalovirus (HCMV), HBV, hepatitis C virus (HCV), herpes simplex virus (HSV), influenza virus, respiratory syncytial virus (RSV), varicella zoster virus (VZV), and human papillomavirus (HPV). The drugs may be administrated as mono- or combined therapies.^186^

Despite the advances reached during the last years, new strategies are needed to tackle several critical unsolved issues in antivirals: resistance mechanisms, poor permeability through cell membranes, low selectivity, low stability during storage and application, and being unable to withstand the conditions of the gastrointestinal tract (hindering the oral administration). Moreover, antivirals are renowned for their high cost and toxicity. One of the most common and critical toxicities is related to their proneness to crystallize, which may cause acute kidney failure, seriously limiting therapy concentration.^187,187−189^

Some of these limitations can be overcome using nanotechnology once it is possible to design the nanoparticles (e.g., composition, morphology, dimensions, and surface characteristics) to improve the handling, stability, absorption, and potency of antivirals.^190^ Most of the research studies comprising nanoparticles and antiviral agents aim to use nanoparticles as delivery systems, and very few analyze the synergistic antiviral effects that may occur. Different nanomaterials have been studied as delivery vehicles for antiviral drugs, including lipids, polymers, lipid–polymer hybrids, carbon, and metals.^191^ Inorganic nanoparticles present some advantages when compared to organic ones. They are easier to functionalize and possess fewer storage requirements since they are not sensitive to microbial or hydrolytic degradation.^192^ MNPs have been widely explored for their antiviral activity per se, namely: Ag, Au, CuO, SiO_2_, TiO_2_, and CeO_2_. These nanoparticles have exhibited pronounced efficacy against several viruses such as influenza (H3N2 and H1N1), HBV, HSV, HIV-1, dengue virus type-2, foot and mouth disease virus, and vesicular stomatitis virus. The MNP functionalization with silane or thiol groups has been displayed to improve the interaction of MNPs with biomolecules. They simultaneously enhanced the impedance of viral internalization in cells and allowed the release of the antiviral drugs.^191^ The research works combining MNPs and antiviral agents are very limited, and only 4 studies against influenza H1N1 virus were found in the literature using AgNPs (3 studies) and AuNPs (1 study) (Table 9).

**Table 9:** Studies between MNPs and Antiviral Agents Obtained by the MNP Synthesis in the Presence of Antivirals: Method B

MNPs, size (nm)	MP synthesis	reducing agent	stabilizing agent	combined antiviral agent	virus	antimicrobial results	ref
Ag, 2.0	chemical	vitamin C	n.a.	zanamivir	H1N1	synergism	(193)
Ag, 2.0	chemical	vitamin C	n.a.	amantadine	H1N1	synergism	(194)
Ag, 2.0	chemical	vitamin C	n.a.	oseltamivir	H1N1	synergism	(195)
Au, 2.0	chemical	sodium borohydride	oseltamivir	oseltamivir	H1N1	n.a.	(196)

Here, just chemical methods were found to prepare the MNPs, and all the studies used method B to prepare the MNP conjugate dispersions. These conjugates displayed interesting synergistic effects. The MNPs were combined with antiviral drugs approved to treat H1N1 infections (zanamivir, amantadine, and oseltamivir). The AgNP–antiviral conjugates depicted monodisperse, highly uniform 2 mm spherical particles. Curiously, the AgNPs before functionalization were 3 nm. The superior stability was due to an increase in the zeta potential.^193−195^ Li and co-workers prepared zanamivir, amantadine, and oseltamivir modified AgNPs and investigated the suppression mechanisms of H1N1 viral infections. The conjugates exhibited notable thermodynamics and kinetics stability. More importantly, they and others displayed evident synergistic virus inactivation properties against the influenza virus.^193−195,197^ Stanley et al. studied, for the first time, influenza therapeutics and diagnostics targeting neuraminidase (instead of hemagglutinin) by combining AuNPs and oseltamivir. It was observed that the conjugates interacted with the virus neuraminidase rather than the hemagglutinin. This highlighted the potential of the conjugates to work as novel influenza virus sensors.^196^ Although the results were promising, the application of these concepts in a clinical environment still requires enormous researcher effort.

## 6 Mechanism of Action and Resistance

The conjugation of MNPs and commercial antimicrobial drugs provides conditions to improve antimicrobial activity. The conjugation of MNPs and antimicrobial drugs allow the simultaneous activation of several *modus operandi*. However, their mechanism of action is still poorly understood. This section analyses the studies performed to understand the mechanism of action and resistance of the MNPs alone or in combination with antibiotics, antifungals, and antivirals. The MNPs and common antimicrobial agents display different mechanisms of action and, consequently, distinct resistance strategies.

### 6.1 MNPs

**Scheme 3:**
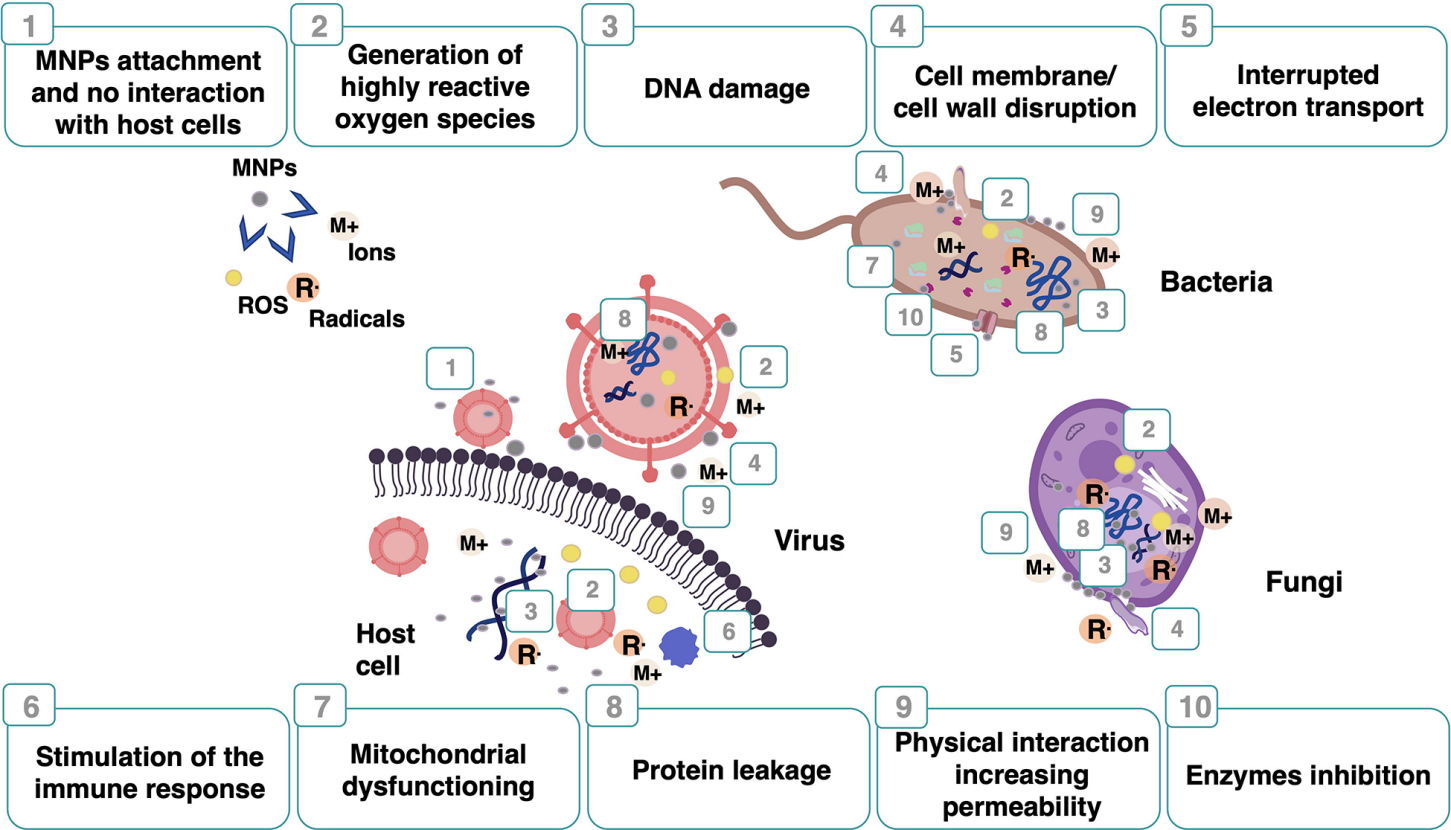
Mechanism of Action of MNPs as Antimicrobial Agents

The antimicrobial mechanism of MNPs is not entirely understood, but it is possible to follow the sequence of events that influence their action as reported in Scheme 3. First, the electrostatic interactions of MNPs with the phospholipid layer of the cell membrane or cell wall components may induce their disruption. Adsorption of MNPs leads to cell wall depolarization, changing the typically negative charge of the wall that becomes more permeable. Due to the disruption, water from the cytosol is released, and the cells try to compensate for the loss through proton efflux pumps and electron transport. Therefore, the microorganism homeostasis is severely compromised due to the imbalance of ions, which impair respiration, interrupt energy transduction and, ultimately, lead to cell death. Moreover, the interactions of MNPs with sulfur-containing molecules within the cell membrane and the metal ions hinder cell wall synthesis. Another antibiotic mechanism of action is the production of ROS and the release of metal ions. These species can denature proteins and damage RNA, DNA, and lipids. Thus, if the cell antioxidant defenses are overwhelmed, ROS can influence the cell wall and membrane permeability, impair enzymatic activity and protein translation, and inhibit ATP production and genetic material replication. The capping agents of MNPs have an important influence in these steps: they can improve or reduce the release of ROS or ions. The MNPs and ions can also bind to cytosolic proteins such as enzymes and nucleic acids.^198,199^

The antiviral function of MNPs can be due to the inhibition of the virus penetration into the cell by the MNP linkage with the virus and stimulation of the nucleus to increase the immune response of the host cell.^200^

Some of the newly reported MNP resistance mechanisms comprise electrostatic repulsion, ion efflux pumps under nonbactericidal concentrations, expression of extracellular matrices, and adaptation through mutations.^201^ However, more studies are needed to unravel the mechanisms behind each of these processes.

### 6.2 Antibiotics

**Scheme 4:**
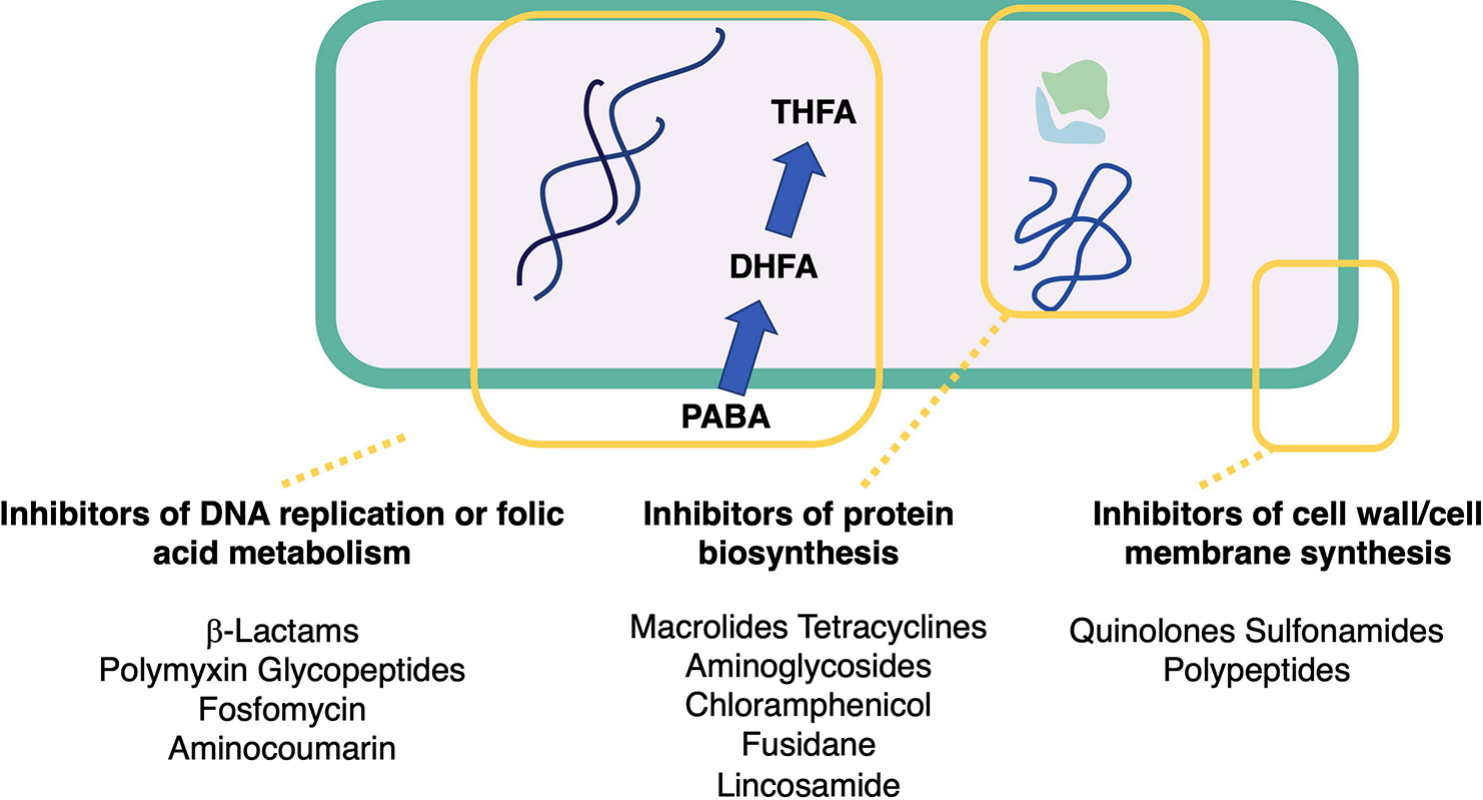
Mechanism of Action of Antibiotics

Antibiotics *modus operandi* are well-known and may be divided by their specific targets: biosynthesis (cell wall and proteins), genome replication, and folic acid metabolism (Scheme 4).^202^ However, several resistance mechanisms have emerged with the appearance of enzymes able to destroy the antibiotic structures, namely, β-lactamase enzyme and chloramphenicol acetyltransferase. Furthermore, mutations in the antibiotic targets, for example, in the enzyme dihydropteroate synthase (DHPS) (the target of sulphonamides), and overexpression of efflux pumps reduced the drug accumulation.^203−208^ Resistance may also occur by replacing the negatively charged groups in the bacterial membrane by neutral groups, thus diminishing the potential electrostatic interaction with MNPs and antibiotics. In addition, genetic mutations may unfold in encoded transport systems.^209−213^

**Scheme 5:**
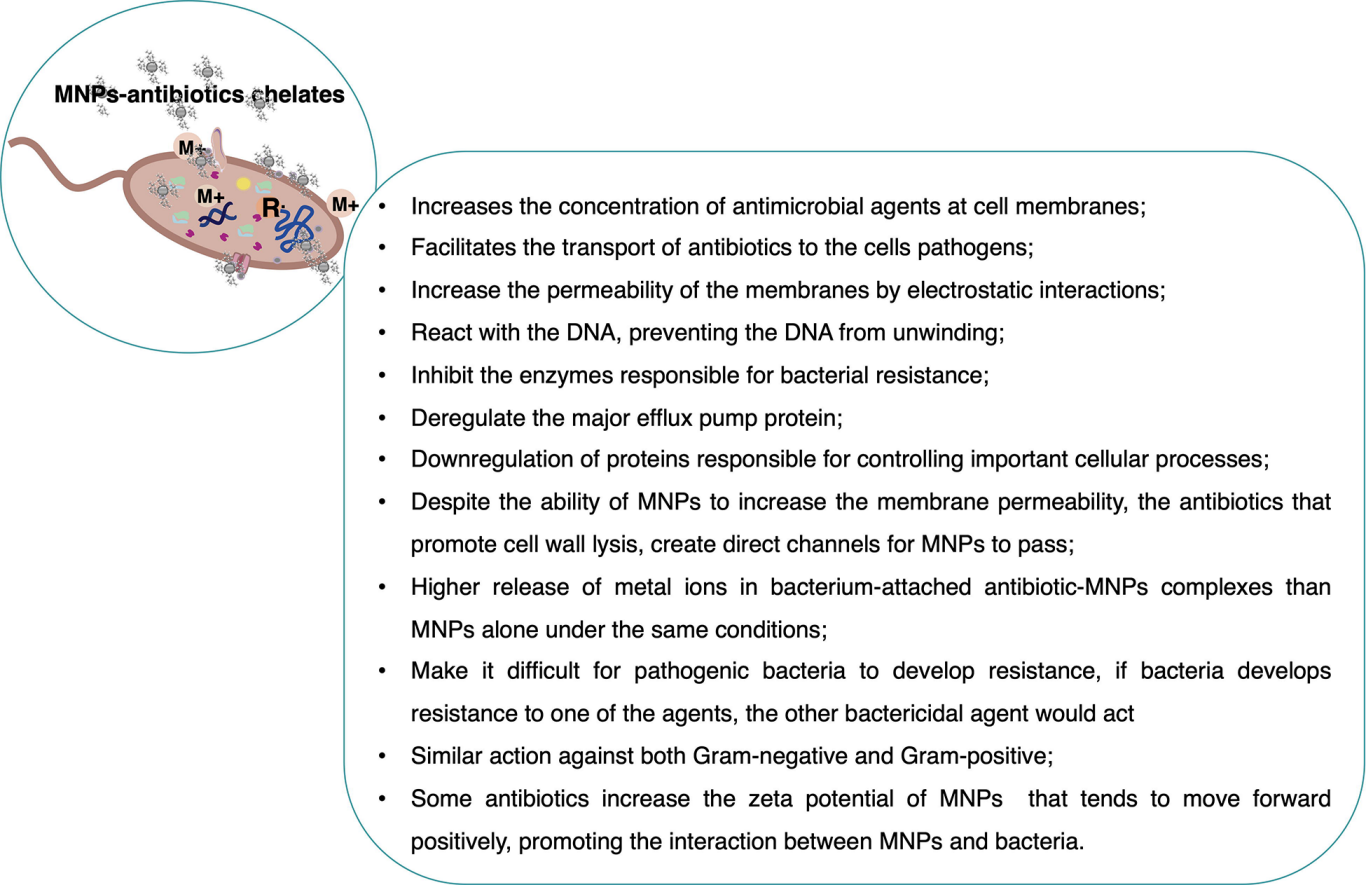
Advantages of MNP–Antibiotic Conjugates and Their Mechanism of Action

The synergistic mechanism of action of combined MNPs and antibiotics is described in Scheme 5. Several works studied the bonding between antibiotics and MNPs by chelation. This chelation increases the concentration of antimicrobial agents at specific points on the cell membrane, where MNPs, acting as a drug carrier, facilitate the transport of antibiotics to the cell surface. In particular, the affinity of AgNPs and AuNPs to sulfur-containing proteins of the bacterial cell membrane enhances the interactions with cells, increasing the permeability of the membranes. This facilitates the infiltration of the antibiotics into the cell. The MNP–chelates can also react with the DNA, increasing the unwound DNA, which due to its higher susceptibly to damage, may result in lethal mutations.^89,117,119,153^

The enzymes responsible for the antibiotic hydrolysis, such as lactamase and carbapenemase, may be inhibited by MNPs, maximizing antibiotic activity.^101^ Gupta et al.^149^ used ethidium bromide (EtBr), which is widely used as a substrate for efflux pumps in cells, to determine the ability of MNPs to act as efflux pump inhibitors. *E. coli* was incubated with hydrophobic AuNPs, and downregulation of the expression of the efflux pumps was observed. The efflux pumps are renowned for their contribution to antibiotic resistance for their role in detoxification. Furthermore, some of the proteins responsible for the assembly of the bacterial outer membrane proteins were strongly deregulated, compromising the integrity of the cell wall. Therefore, synergism between MNPs and antibiotics may be potentiated by the deregulation of major efflux pump proteins.

The hydrophobic nanoparticles can also interact with multiple proteins to disrupt crucial cell survival processes, enhancing the efficacy of the antibiotic.^149^ Wei et al. conjugated AgNPs with norvancomycin. They observed that the permeability of the outer membrane was affected by the AgNP attachment to the lipopolysaccharide membranes, leading to its destabilization and allowing the norvancomycin action.^148^ Fayaz et al. explained the ampicillin–AgNP mechanism against bacteria. First, the ampicillin molecules surround AgNPs by electrostatic attraction. Then, the ampicillin promotes the cell wall lysis creating channels that allow the penetration of AgNPs into the bacteria. The ampicillin–AgNP complex reacts with DNA and prevents DNA from unwinding, which seriously compromises cell viability.^118^ Bhande et al. provided a possible explanation for the enhancement of the synergistic antibacterial mechanism of β-lactam antibiotics and ZnONPs. The contact of ZnONPs with the cell wall and the consequent penetration are more accessible when surrounded by β-lactam antibiotics. Inside the cell, the ZnONP–antibiotic complex reacts with DNA resulting in critical genome damage.^107^ Another research work showed a higher release of metal ions in antibiotic–MNP complexes than AgNPs alone under the same conditions. A localized transient high metal ion concentration near the bacterium’s surface was observed. The metal ions bind to proteins and nucleic acids, causing bacterial death.^103^

These studies suggested that simultaneous action of antibiotics and AgNPs will make it difficult for pathogenic bacteria to develop resistance. If bacteria develops resistance to one agent, the other bactericidal mechanism will kill the bacteria.^94,123^ Another interesting and important fact was the similar synergistic effects against both Gram-negative and Gram-positive bacteria, indicating that the difference in cell wall composition did not influence synergistic efficiency.^101^ In addition, the size and surface properties of MNPs can directly influence the synergistic effects of MNPs when combined with antibiotics. In synergistic studies, smaller MNPs were shown to improve the antimicrobial properties. The effect of the capping agent was also described, and the results showed a more prominent effect with PVP-capped AgNPs as compared to citrate- and SDS-capped ones. As expected, the more positive the charge of the MNPs, the better is the antibacterial action. When the antibiotic gentamicin was added to a MNP dispersion, the zeta potential of the MNPs displayed a more positive charge, promoting the interaction between MNPs and bacteria.^98^ Experimental data related to synergistic effects of different shaped AgNPs with various antibiotics have not been reported yet in the scientific literature.^101^ A long-lasting, single-particle treatment capable of overcoming antibiotic resistance would be highly beneficial in the clinical environment.^214^

### 6.3 Antifungals

**Scheme 6:**
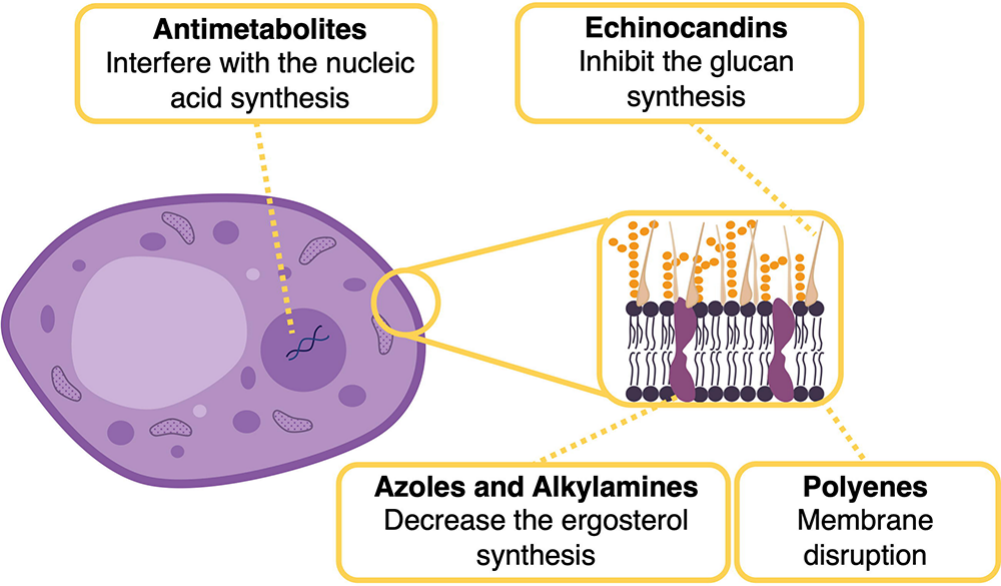
Mechanism of Action of Antifungal Agents

Antifungal drugs mainly have two targets: cell membrane or nucleic acid synthesis. The azoles, alkylamines, polyenes, and echinocandins destabilize the cell membrane, and antimetabolites interfere with nucleic acid synthesis (Scheme 6).

Most of the resistance mechanisms related to antifungal drugs involve target modifications or overexpression of efflux pumps that expel the drug out of the cell, decreasing its intracellular concentration. The target mutation and/or target expression deregulation is another resistance mechanism. For example, the ERG11 gene encodes the enzyme lanosterol 14α-demethylase in yeasts, the target enzyme for azoles. Thus, a mutation or overexpression in this gene alters the azole-binding site, requiring a higher drug concentration. The last mechanism in antifungals’ resistance is attributed to alterations in the ergosterol biosynthesis pathway, where the mutation in the gene encoding the lanosterol 14α-demethylase enzyme also modifies other enzymes from the same biosynthetic pathway.^215,216^

Few studies about the mechanism of action of MNPs combined with antifungals can be found in the literature. Kumar and Poornachandra mentioned that the conjugation of AgNPs with miconazole increased the drug’s efficacy and played a dual mechanism of action by ROS accumulation and inhibition of the ergosterol biosynthesis.^163^ Sun et al. showed that the binding of PVP-coated AgNPs with fluconazole enhanced the antifungal properties. The author theorizes that the antifungal action may be due to the remodeling of the cell membrane in the azole-resistant strains or to an azole transport-specific mechanism. The researchers also noted an increased inhibition of the normal budding process. The mechanism of synergy between PVP-AgNPs and nystatin or chlorhexidine digluconate was attributed to the actions of both MNPs and antifungal drugs. On the other hand, the synergy mechanism between PVP-AgNPs and fluconazole or voriconazole was attributed to the MNP tendency to adhere to the cell membrane and inhibit the budding replication. In addition, the dysregulations of the ergosterol pathway and efflux pumps may serve as a crucial contributor to the synergy between PVP-coated Ag and fluconazole or voriconazole.^165^

### 6.4 Antivirals

**Scheme 7:**
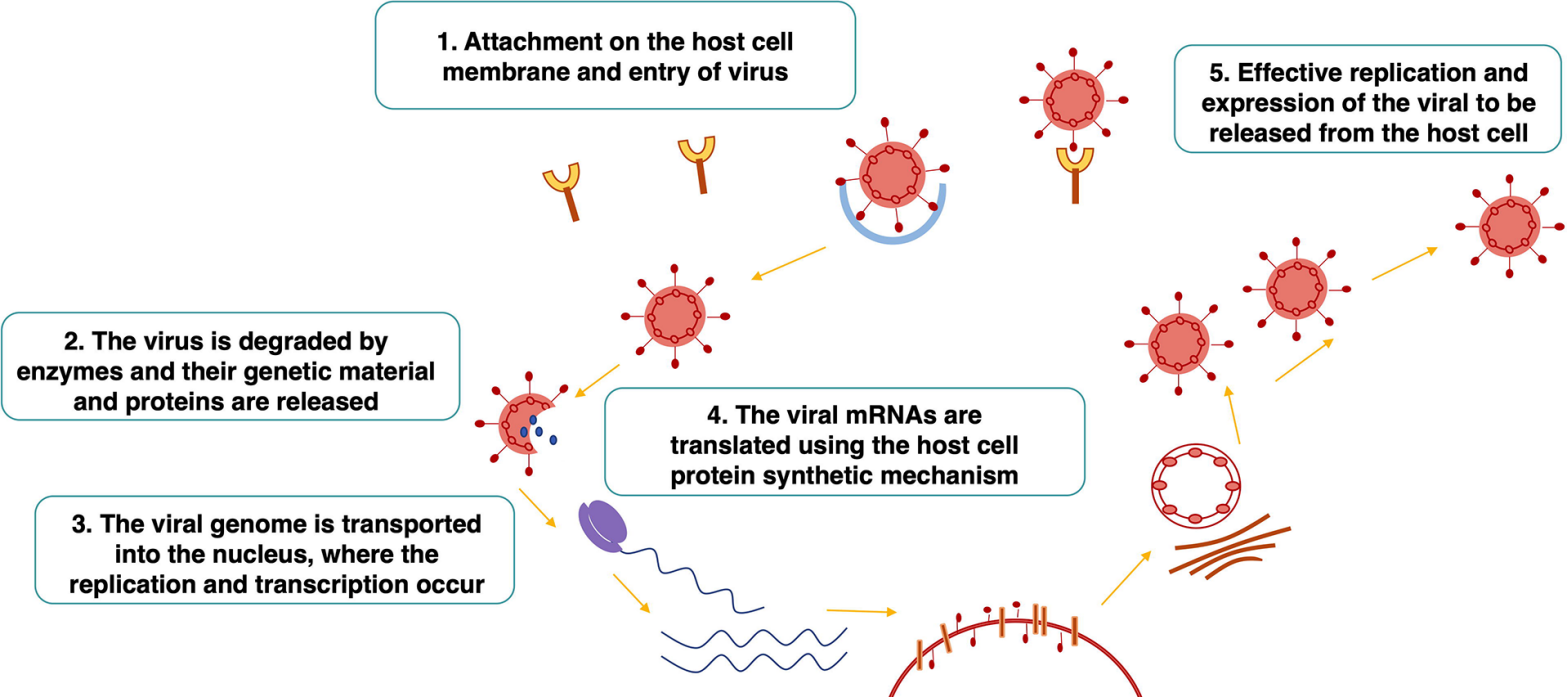
Mechanism of Viral Infection

The development of antiviral agents is challenging, even though similarities in infection processes exist. Viral infection progression may be considerably different among distinctive strains. The most frequent stages in viral infections are (i) attachment, (ii) penetration, (iii) uncoating, (iv) gene expression and replication, and (v) assembly and release (Scheme 7). Antiviral drugs are designed to block one or more of these steps.^188,217^

The available antivirals are frequently restricted by their short spectrum, the rapid emergence of drug resistance, and toxicity.^218^ The approved antiviral drugs include: 5-substituted 2′-deoxyuridine analogues, nucleoside analogues, (nonnucleoside) pyrophosphate analogues, nucleoside reverse transcriptase inhibitors, nonnucleoside reverse transcriptase inhibitors, protease inhibitors, integrase inhibitors, entry inhibitors, acyclic guanosine analogues, acyclic nucleoside phosphonate analogues, hepatitis C virus NS5A and NS5B inhibitors, influenza virus inhibitors and immunostimulators, interferons, oligonucleotides, and antimitotic inhibitors.^186^

A major challenge facing antiviral drug development is resistance. Indeed, drug resistance is a commonly reported issue affecting approved antiviral drugs that directly act against a viral target or virus–host interaction. Drug resistance is particularly problematic concerning RNA viruses due to their rapid rate of viral replication and frequent recombination events. The availability of novel drugs with different mechanisms of action or combination therapy can improve the treatment outcome.^219^ It was previously assumed that viruses could not develop drug resistance against agents that target host factors needed for virus replication once viruses cannot easily replace the missing cellular functions by mutagenesis. However, emerging evidence suggests that viral resistance against host-directed antiviral agents can occur by mutations, such as in fusion protein, mutations near the active site, RNA-dependent RNA polymerase, viral proteins, viral polymerase, and envelope proteins. While not yet fully understood, one possible mechanism underlying the acquisition of drug resistance against host-directed agents is that the virus may use an alternate host factor. Other examples include viruses that have evolved diverse strategies to modulate a host translational apparatus. The most understood mechanism of antiviral drug resistance against virus-directed therapies is that mutations occur in the viral genome at druggable sites. These alter viral susceptibility to the direct action of drugs. Moreover, the precise nature of host factors that may regulate the phenomenon of drug tolerance remains elusive. Model systems are urgently required to evaluate drug resistance/synchronization under complex and dynamic settings, such as drug combinations, multiple viral infections, and seasonality.^220^

The antiviral mechanism of action of conjugates is an unexplored field. Just one group of researchers studied the mechanism of action of an antiviral drug against the influenza virus. Li et al. revealed that AgNP–amantadine/oseltamivir could block the H1N1 virus from infecting host cells and prevent DNA fragmentation, chromatin condensation, and the activity of caspase-3. The conjugates inhibited the accumulation of reactive oxygen species (ROS) and reversed virus-induced apoptosis by the H1N1 virus.^194,195^ The same group in 2017 used flow cytometric analysis and the TUNEL–DAPI assay to evaluate the antiviral mechanisms of AgNP–zanamivir. The potential molecular mechanisms revealed that AgNP–zanamivir inhibited caspase-3 mediated apoptosis via ROS generation.^193^ Overall, the synergistic actions and the mechanism behind them showed several advantages, but most studies are highly speculative and need further investigation. However, the topic requires more exploration. It is crucial to investigate the effects of MNPs with other drugs to fully understand the bioeffects of these complex systems in the virus.

## 7 Cytotoxicity

Cytotoxicity of human exposure to MNPs has rightfully gained attention in the last years. Since these nanomaterials have been intentionally engineered to interact with cells in biomedical applications, it is important to ensure that these activities do not create adverse effects on the human body.^221^ The negative effects of MNPs are related to their physical and chemical properties, including the size, shape, surface charge, chemical compositions (core and shell), and stability. Many types of MNPs are not recognized by the cells protective systems of the human body, which decreases the rate of their degradation and may lead to considerable accumulation of nanoparticles in organs and tissues, resulting in highly toxic and lethal concentrations. Several approaches to design new nanoparticles with lower toxicity than traditional nanoparticles are already available. Advanced methods for the study of the toxicity of the nanoparticles make it possible to analyze different pathways and mechanisms of toxicity at the molecular level and predict possible negative effects on the body. The data relating to the adverse and toxic effects of AgNPs differs strongly in the literature, and several conclusions are controversial.^222^ Moreover, commercial antimicrobial drugs (antibiotics, antiviral, and antifungal) show several issues related to their tolerance. Antibiotic resistance and toxicity are the major limiting factors in the use of antibiotics. Antibiotic toxicity can cause hypersensitivity reactions, blood dyscrasias, nephrotoxicity, neurotoxicity, ototoxicity, and hepatic and renal toxicity.^223^ In antifungal therapies, hepatotoxicity incidence rates are induced by antifungal therapy with azoles.^224^ Despite polyenes’ highly favorable antifungal activity, the treatment remains difficult due to toxicity in critical mammalian organ systems, mainly nephrotoxicity, caused by its lack of selectivity between fungal and animal sterols. The antimetabolites have expressed hepatotoxicity and bone marrow depression in combination therapies.^225,226^ The antiviral drugs have been related to nephrotoxicity (even in low doses) (adefovir), renal and bone toxicity (patients with HIV), and high levels of renal toxicity (acyclovir).^186,227^ In synergistic studies involving MNPs and commercial antimicrobial drugs, testing the cytotoxicity is a factor of extreme importance. However, only the more recent reports (since 2015) include cytotoxicity tests for conjugates. A careful analysis of the literature found only 17 works discussing the toxicity of the conjugates. These studies were carried out using colorimetric tests, namely, the 3-[4,5-dimethylthiazole-2-yl]-2,5-diphenyltetrazolium bromide (MTT) assay or 3-(4,5-dimethylthiazol-2-yl)-5-(3-carboxymethoxyphenyl)-2-(4-sulfophenyl)-2*H*-tetrazolium (MTS) assay. The tests were performed with mortal cells such as NIH 3T3 fibroblasts, keratinocyte HaCaT cells, human embryonic kidney cells (HEK293T), human colon epithelial cells (CCD-841CoTr), human retinal pigment epithelial-1 cells (RPE-1), human gingival fibroblasts (MD-HGF), and mouse peritoneal macrophages. In addition to immortal cells, human acute monocytic leukemia cells (THP-1), human breast cancer cells (MCF7), and rat glioma cells C6 (Table 10) were used.

**Table 10:** Cytotoxicity Synergic Studies between MNPs and a Combined Commercial Antimicrobial Agent

MNPs, size (nm), stabilizing agent	combined commercial antimicrobial agent	pathogens	cell lines	test: cytotoxicity result	ref
Ag, 7.4–18.3, n.a.	cefotaxime	*E. coli* and MRSA	MCF-7 and RPE-1 cells	MTT: no cytotoxic effect on normal cells even at 12 μg/mL for 24 h	(126)
Ag, 12.0, PVP	ampicillin	DH5α susceptible and ampicillin-resistant *E. coli* strains	HEK293T	MTS assay: no significant viability reduction	(147)
Ag, 23.0, citrate	tetracycline, neomycin, and penicillin G	*S. typhimurium* DT104 (ATCC 700408)	HaCaT	MTS assay: tetracycline, AgNPs were not toxic; penicillin, AgNPs were slightly toxic to cells; neomycin, AgNPs slightly stimulated HaCaT cell growth at the 24 h exposure time	(97)
Ag, 25.0, n.a.	vancomycin	*S. aureus* and *E. coli*	mouse peritoneal macrophages	MTT: no cytotoxic effect at 0.05, 0.1, and 0.3 mM concentrations after 24 h	(99)
Ag, 26.0, n.a.	erythromycin, ampicillin, chloramphenicol, cephalothin, clindamycin, tetracycline, gentamycin, amoxicillin, ciprofloxacin, ampicillin, cefpodoxime, and cefuroxime	*S. aureus*, MRSA, *S. mutans*, *S. oralis*, *S. gordonii*, *E. faecalis*, *E. coli*, *A. actinomycetemcomitans*, and *P. aeruginosa*	MD-HGF	live/dead assay: using a low concentration of AgNPs (1.0 mg/mL), the viability of primary human fibroblasts was over 80% even after 7 days of direct culture with the AgNPs	(100)
Ag, 26, gelatin	ampicillin, ampicillin/sulbactam, cefazolin, cefuroxime, cefoxitin, gentamicin, co-trimoxazole, colistin, oxolinic acid, ofloxacin, tetracycline, aztreonam, piperacillin, piperacillin/tazobactam, meropenem, ceftazidime, cefoperazone, cefepime, amikacin, ciprofloxacin, penicillin, oxacillin, chloramphenicol, erythromycin, clindamycin, ciprofloxacin, teicoplanin, and vancomycin	*E. coli* CCM 4225, *P. aeruginosa* CCM 3955, and *S. aureus* CCM 4223	NIH/3T3	MTT assay: >70% of cell viability in tests with MIC concentrations and >90% using sub-MIC concentrations	(101)
Ag, 28, n.a.	cefotaxime, ceftazidime, meropenem, ciprofloxacin, and gentamicin	susceptible and resistant *E. coli* and *K. pneumoniae*	NIH/3T3	MTT assay: AgNPs, antibiotics alone, and AgNP–antibiotic combinations at concentrations of 4 and 2 mg/L, respectively, showed no cytotoxic effect on the mammalian cell lines	(102)
Ag, 44.1, n.a.	ampicillin	*E. coli*, *S. aureus*, ampicillin-resistant *E. coli*, *S. aureus*, multidrug-resistant *P. aeruginosa*, and *K. pneumonia*	HaCaT	MTT assay: nontoxic	(152)
CuS, 15.0, n.a.	vancomycin	*E. faecium* and *E. faecalis*	3T3 and C6	MTT assay: slightly reduced cell viability was yielded when cells were treated with conjugates at the concentration of 16 μg/mL upon NIR irradiation	(154)
Ag-zeolite, 80.0, citrate	fluconazole, caspofungin, and micafungin	*C. albicans* 451	HUVEC	MTT assay: cytotoxicity similar to AgNPs alone	(166)
Ag, 7.0, amphotericin B	amphotericin B	*A. niger*, *C. albicans*, and *F. culmorum*	CCD-841CoTr and THP-1	MTT assay: >80% of cells viable in amphoteric B–AgNPs (1:11 molar ratio)	(175)
Ag, 2.0, n.a.	amantadine	H1N1	MDCK	MTT assay: >90% of cell viability	(194)
Ag, 2.0, n.a.	oseltamivir	H1N1	MDCK	MTT assay: >90% of cell viability	(195)

**Figure 3:**
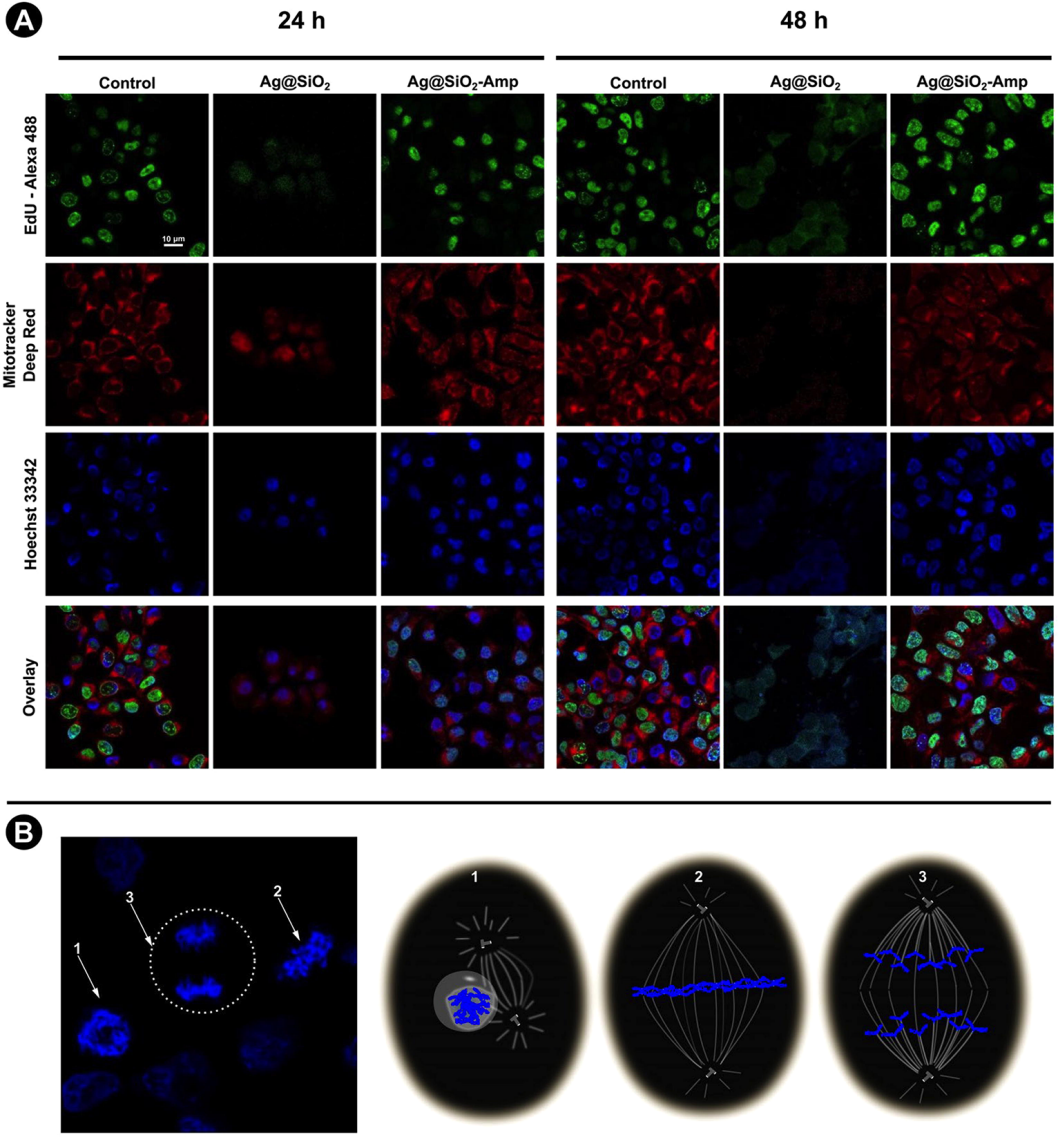
Cell images during the cytotoxicity tests: (A) the first column corresponds to the control test in the absence of nanoparticles for 24 and 48 h of treatment (just ampicillin), the second column corresponds to the cells in the presence of the Ag–SiO_2_, and the third column corresponds to the cells in the presence of the Ag–SiO_2_–ampicillin. The “EdU-Alexa 488” row represents proliferating cells; the “MitoTracker Deep Red” row indicates mitochondria in the cytoplasm; the “Hoechst 33342” row represents the cell nuclei; the last row represents the overlay of the three images for each condition. (B) Mitoses phases were observed in confocal images. The white arrows in the confocal image and Schemes 1, 2, and 3 represent prophase, metaphase, and anaphase, respectively, after 48 h of Ag–SiO_2_–ampicillin treatment. Reproduced with permission from ref (147). Copyright 2017 Springer Nature.

Khatoon et al. tested the cytotoxicity of ampicillin–AgNPs against keratinocyte cell line HaCaT. The conjugates were found to be nontoxic to mammalian cells with significant antibacterial activity against ampicillin-resistant and multidrug-resistant bacteria.^152^ McShan et al. evaluated the cytotoxicity of tetracycline, neomycin, penicillin G, and AgNPs on HaCaT cells to understand whether AgNPs and antibiotics alone or their combination are toxic to human cells in three exposure periods (0.5, 2, and 24 h). They also tested the silver nitrate cytotoxicity as a control. AgNO_2_ was toxic, and its toxicity increased throughout the experiment. At the same concentration, AgNPs showed no toxicity. Also, the three antibiotics were nontoxic up to 16 μM. The tetracycline–AgNP conjugates were not toxic for all three exposure times; penicillin–AgNPs were slightly toxic to the cells, while neomycin–AgNPs slightly stimulated HaCaT cell growth after 24 h of exposure.^97^ Panáček et al. assessed the cytotoxicity of AgNPs, antibiotics with a different mode of action, and their combinations at concentrations equal to and under the MIC values. In the case of cytotoxicity evaluation at concentrations similar to MIC values, AgNPs and antibiotics only slightly inhibited the viability of the cells. When antibiotics were combined with AgNPs, the viability of the cells decreased from 85% to 71% compared to the control. The cytotoxic effect was higher due to the additive cytotoxicity of the antibiotics and AgNPs. A combination of antibiotics and AgNPs shows the highest inhibition of the cell’s viability at concentrations equal to their MIC. In the studies at lower concentrations, below the MIC but still showing antibacterial activity, the agents did not hinder cell viability. When antibiotics were combined with AgNPs, the viability of the cells only decreased to 90–95% compared to the control. It may be assumed that the prevention or treatment of infections would be more effective when the antibiotic combination with the AgNPs occurred at very low concentrations of both antimicrobial substances, minimizing the risk of toxic side effects, since no cytotoxicity was observed in NIH/3T3 cells. However, the adequate dose to be used is still unclear, thus requiring further research to determine if AgNPs combined with antibiotics can be effective for the local and systematic therapy of infectious diseases without showing any side or adverse effects.^101^ de Oliveira et al. studied the possible cytotoxic effect of the Ag–SiO_2_ or Ag–SiO_2_–ampicillin at the highest concentration used during the bactericidal tests using HEK293T cells. The Ag–SiO_2_ system showed a strong cytotoxic effect for both treatment periods, reducing the cell viability to approximately 20% after 48 h of incubation. On the other hand, Ag–SiO_2_–ampicillin showed promising results since no significant viability reduction was observed. The researchers also studied the mitosis phases of Ag–SiO_2_–ampicillin-treated HEK293T cells. The observation during the three mitosis phases, prophase, metaphase, and anaphase, suggest that, for at least 48 h, almost no toxicity or cell growth inhibition was observed in the presence of Ag–SiO_2_–ampicillin and that the antibiotic probably acts as a toxic-protective organic molecule. MNPs coated with ampicillin were not able to interfere during the cellular metabolism since different mitosis cell phases were seen in the presence of Ag–SiO_2_–ampicillin (Figure 3).^147^

**Figure 4:**
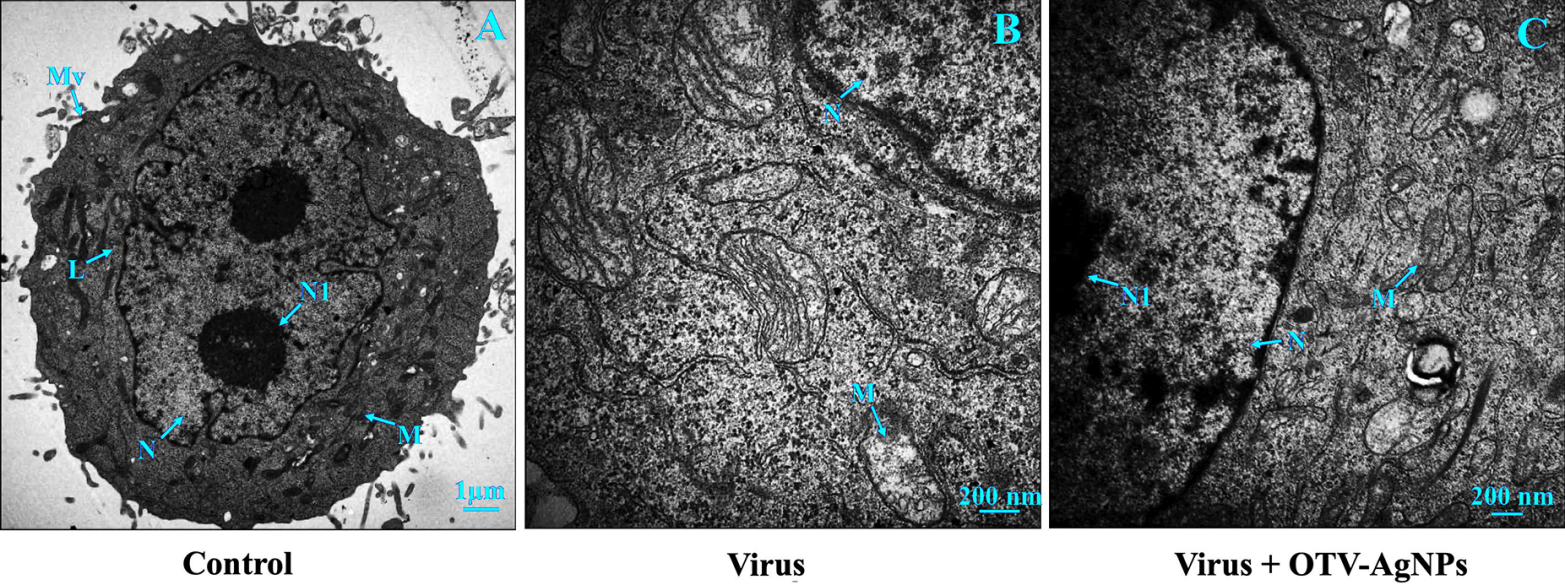
TEM images of thin sections of MDCK cells: (A) control, (B) cells treated with virus, and (C) cells treated with virus and oseltamivir–AgNPs (N: nucleus; N1: nucleolus; M: mitochondria; L: lysosome; Mv: microvillus; Ag: silver; OTV: oseltamivir). Reproduced from ref (195). Copyright 2016 American Chemical Society.

Li et al. evaluated the drug combination of AgNPs at sublethal concentrations with echinocandin drugs. The authors tested the toxicity of the combination with mammalian HUVECs. The combination of echinocandin drugs and a sublethal dose of 80 nm AgNPs showed relatively low cytotoxicity to the mammalian cells. Thus, the combination of echinocandin drugs and AgNPs at sublethal levels could become a new strategy for the clinical treatment of infections with antifungal-resistant strains or even for new drug development.^166^ Tutaj et al. tested the cytotoxicity of amphotericin B–AgNPs in CCD-841CoTr and THP-1 cell lines since colon epithelial cells serve as a model to evaluate amphotericin B transport across the intestinal barrier and monocytes can accumulate the drug. The results of the cytotoxic studies revealed the statistically lower toxicity of amphoteric B–AgNPs (1:11 molar ratio) in comparison with amphotericin B alone (>80%). The differences might be due to the different molecular organization of amphotericin B in each formulation. Amphotericin B alone is in the aggregated form, while in the nanoformulations, amphotericin B is in the monomeric state due to immobilization of the molecule on the MNP surface.^175^ Li et al. studied the cytotoxic effects of the H1N1 influenza virus on MDCK cells and the protective effects of amantadine–AgNPs and oseltamivir–AgNPs by the MTT assay. MDCK cells treated with the H1N1 influenza virus showed cell viability of 39%. Amantadine, oseltamivir, and AgNPs increased the cell viability to 56%, 59%, and 65%, respectively. However, the cell viability was increased to 90% with amantadine–AgNP or oseltamivir–AgNP combinations.^194,195^ A change of the morphology of MDCK cells treated with oseltamivir–AgNPs was observed by TEM. The microvilli and mitochondria showed no morphological alterations in the untreated cells. When incubated with the H1N1 influenza virus, TEM images indicated cells with the disappearance of microvilli, a shrinking cytoplasm, distorted organelles, and condensed chromatin, indicating apoptosis of the MDCK cells. The percentage of cells that lost adhesion and shrunk was decreased after treatment with oseltamivir–AgNPs (Figure 4).^195^

Generally, the cytotoxicity studies are not directed by a specific route in which the MNP conjugates enter the body. Although some works show very interesting results in terms of cytotoxicity, more studies are needed before their therapeutic use.

## 8 Conclusion, Challenges, and Perspectives

This literature review aimed to survey the developed methods and respective results of the conjugation of MNPs with commercial antimicrobial drugs, including antibiotics, antifungals, and antivirals. It was verified that many metals and metal oxides had shown properties of high interest, namely, as drug delivery systems, anticancer therapies, and antimicrobial drugs. However, very few MNPs have been approved for clinical use by the FDA and EMEA. Standards should be urgently developed to decrease the risk of toxicity of nanoparticles and the negative effects of exposure. The pharmacokinetics of the MNPs and the conjugates should be studied to induce high selectivity and reduce the accumulation in nontargeted cells.

A limited number of materials were considered in the preparation of conjugated agents. Thus, several possible combinations emerge and may be studied in future works. Also, several challenges arise in the methods for MNP synthesis and surface functionalization. The environmental and safety component should be improved during the MNP preparation.

Regarding the methods for MNP conjugation, method A was the most used. In this method, the MNPs and antimicrobial agents are only stabilized by ionic interactions and/or the formation of chelates. The MNP surface functionalization can be further explored since covalently bonded synergism is preferred over simple physical adsorption to prevent leaching of the drug from the nanoparticles. In most studies, the conjugation of MNPs and antimicrobial drugs are performed mainly by surface adsorption, where covalent bonding rarely appears. Here, a limited number of reports exist, and these reports mostly focus on AuNPs. Furthermore, in most of the research works, the complete physicochemical characterization of conjugates is not available, making it difficult to associate the MNP design with their antimicrobial effect. This is particularly grievous for works describing simple conjugation (method A).

MNPs per se possess interesting antimicrobial properties, but their conjugation with available agents tends to exhibit relevant advantages, especially by diminishing cytotoxicity.

The antimicrobial performance of the conjugates was challenging to compare due to the use of different evaluation methodologies. Thus, the calculation of the FIC in future works is recommended to allow a comparison and decision-making for the most improved conditions for synergy.

MNPs have been successfully combined with several antibiotic and antifungal molecules. Nevertheless, the combination of MNPs with antivirals remains extremely limited. Mechanistic studies of MNP conjugates are very limited. However, the simultaneous action of MNPs and antimicrobial agents seems to represent an important strategy to circumvent pathogenic resistance and impede its establishment.

In most cases, the conjugation presented lower cytotoxicity than the individual agents, and it is possible to obtain practical antimicrobial effects with lower drug concentrations. Only more recent research works present cytotoxicity studies, and further investigation is needed to determine the optimal balance between effect and toxicity.

Finally, MNP conjugates presented several advantages: decreased individual dosages of drugs, minimized cytotoxicity, and an increased spectrum of antimicrobial coverage.

## References

[ref1] Morsy M. A.; Ali E. M.; Kandeel M.; Venugopala K. N.; Nair A. B.; Greish K.; El-Daly M. Screening and Molecular Docking of Novel Benzothiazole Derivatives as Potential Antimicrobial Agents. Antibiotics 2020, 9 (5), 221 10.3390/antibiotics9050221. 32365587 PMC7277330

[ref2] Ogunsona E. O.; Muthuraj R.; Ojogbo E.; Valerio O.; Mekonnen T. H. Engineered nanomaterials for antimicrobial applications: A review. Applied Materials Today 2020, 18, 100473 10.1016/j.apmt.2019.100473.

[ref3] Mitchell B. G.; Hall L.; White N.; Barnett A. G.; Halton K.; Paterson D. L.; Riley T. V.; Gardner A.; Page K.; Farrington A.; Gericke C. A.; Graves N. An environmental cleaning bundle and health-care-associated infections in hospitals (REACH): a multicentre, randomised trial. Lancet Infectious Diseases 2019, 19 (4), 410–418. 10.1016/S1473-3099(18)30714-X. 30858014

[ref4] D’Andrea M. M.; Fraziano M.; Thaller M. C.; Rossolini G. M. The Urgent Need for Novel Antimicrobial Agents and Strategies to Fight Antibiotic Resistance. Antibiotics 2019, 8 (4), 254 10.3390/antibiotics8040254. 31817707 PMC6963704

[ref5] Vila J.; Moreno-Morales J.; Ballesté-Delpierre C. Current landscape in the discovery of novel antibacterial agents. Clinical Microbiology and Infection 2020, 26 (5), 596–603. 10.1016/j.cmi.2019.09.015. 31574341

[ref6] Haak B. W.; Wiersinga W. J. Uncovering hidden antimicrobial resistance patterns within the hospital microbiome. Nature Medicine 2020, 26 (6), 826–828. 10.1038/s41591-020-0919-z. 32514170

[ref7] Matthiessen L.; Bergström R.; Dustdar S.; Meulien P.; Draghia-Akli R. Increased momentum in antimicrobial resistance research. Lancet 2016, 388 (10047), 865 10.1016/S0140-6736(16)31425-8. 27597459

[ref8] Nathan C. Resisting antimicrobial resistance. Nature Reviews Microbiology 2020, 18 (5), 259–260. 10.1038/s41579-020-0348-5. 32300248

[ref9] Banin E.; Hughes D.; Kuipers O. P. Editorial: Bacterial pathogens, antibiotics and antibiotic resistance. FEMS Microbiology Reviews 2017, 41 (3), 450–452. 10.1093/femsre/fux016. 28486583

[ref10] Ayaz M.; Ullah F.; Sadiq A.; Ullah F.; Ovais M.; Ahmed J.; Devkota H. P. Synergistic interactions of phytochemicals with antimicrobial agents: Potential strategy to counteract drug resistance. Chemico-Biological Interactions 2019, 308, 294–303. 10.1016/j.cbi.2019.05.050. 31158333

[ref11] Nieuwlaat R.; Mbuagbaw L.; Mertz D.; Burrows L. L.; Bowdish D. M. E.; Moja L.; Wright G. D.; Schünemann H. J. Coronavirus Disease 2019 and Antimicrobial Resistance: Parallel and Interacting Health Emergencies. Clinical Infectious Diseases 2021, 72 (9), 1657–1659. 10.1093/cid/ciaa773. 32544232 PMC7337675

[ref12] Tyers M.; Wright G. D. Drug combinations: a strategy to extend the life of antibiotics in the 21st century. Nature Reviews Microbiology 2019, 17 (3), 141–155. 10.1038/s41579-018-0141-x. 30683887

[ref13] Wang L.-L.; Battini N.; Bheemanaboina R. R. Y.; Zhang S.-L.; Zhou C.-H. Design and synthesis of aminothiazolyl norfloxacin analogues as potential antimicrobial agents and their biological evaluation. Eur. J. Med. Chem. 2019, 167, 105–123. 10.1016/j.ejmech.2019.01.072. 30769240

[ref14] Mahira S.; Jain A.; Khan W.; Domb A. J. Chapter 1. Antimicrobial Materials—An Overview. Antimicrobial Materials for Biomedical Applications 2019, 1–37. 10.1039/9781788012638-00001.

[ref15] Arias L.; Pessan J.; Vieira A.; Lima T.; Delbem A.; Monteiro D. Iron Oxide Nanoparticles for Biomedical Applications: A Perspective on Synthesis, Drugs, Antimicrobial Activity, and Toxicity. Antibiotics 2018, 7 (2), 46 10.3390/antibiotics7020046. 29890753 PMC6023022

[ref16] Ramos A. P.; Cruz M. A. E.; Tovani C. B.; Ciancaglini P. Biomedical applications of nanotechnology. Biophysical Reviews 2017, 9 (2), 79–89. 10.1007/s12551-016-0246-2. 28510082 PMC5425815

[ref17] Campos E. V. R.; Pereira A. E. S.; de Oliveira J. L.; Carvalho L. B.; Guilger-Casagrande M.; de Lima R.; Fraceto L. F. How can nanotechnology help to combat COVID-19? Opportunities and urgent need. J. Nanobiotechnol. 2020, 18 (1), 125 10.1186/s12951-020-00685-4. PMC7474329 32891146

[ref18] Chang T.-K.; Cheng T.-M.; Chu H.-L.; Tan S.-H.; Kuo J.-C.; Hsu P.-H.; Su C.-Y.; Chen H.-M.; Lee C.-M.; Kuo T.-R. Metabolic Mechanism Investigation of Antibacterial Active Cysteine-Conjugated Gold Nanoclusters in Escherichia coli. ACS Sustainable Chem. Eng. 2019, 7 (18), 15479–15486. 10.1021/acssuschemeng.9b03048.

[ref19] Yougbaré S.; Chou H.-L.; Yang C.-H.; Krisnawati D. I.; Jazidie A.; Nuh M.; Kuo T.-R. Facet-dependent gold nanocrystals for effective photothermal killing of bacteria. Journal of Hazardous Materials 2021, 407, 124617 10.1016/j.jhazmat.2020.124617. 33359972

[ref20] Yougbare S.; Chang T.-K.; Tan S.-H.; Kuo J.-C.; Hsu P.-H.; Su C.-Y.; Kuo T.-R. Antimicrobial Gold Nanoclusters: Recent Developments and Future Perspectives. International Journal of Molecular Sciences 2019, 20 (12), 2924 10.3390/ijms20122924. 31208013 PMC6627976

[ref21] Li J. J.; Zou L.; Hartono D.; Ong C. N.; Bay B. H.; Lanry Yung L. Y. Gold Nanoparticles Induce Oxidative Damage in Lung Fibroblasts In Vitro. Adv. Mater. 2008, 20 (1), 138–142. 10.1002/adma.200701853.

[ref22] Damasco J. A.; Ravi S.; Perez J. D.; Hagaman D. E.; Melancon M. P. Understanding Nanoparticle Toxicity to Direct a Safe-by-Design Approach in Cancer Nanomedicine. Nanomaterials 2020, 10 (11), 2186 10.3390/nano10112186. 33147800 PMC7692849

[ref23] Huang H.; Feng W.; Chen Y.; Shi J. Inorganic nanoparticles in clinical trials and translations. Nano Today 2020, 35, 100972 10.1016/j.nantod.2020.100972.

[ref24] Dadfar S. M.; Roemhild K.; Drude N. I.; von Stillfried S.; Knüchel R.; Kiessling F.; Lammers T. Iron oxide nanoparticles: Diagnostic, therapeutic and theranostic applications. Adv. Drug Delivery Rev. 2019, 138, 302–325. 10.1016/j.addr.2019.01.005. PMC7115878 30639256

[ref25] Anselmo A. C.; Mitragotri S. Nanoparticles in the clinic: An update. Bioengineering & Translational Medicine 2019, 4 (3), e10143 10.1002/btm2.10143. 31572799 PMC6764803

[ref26] Burduşel A.-C.; Gherasim O.; Grumezescu A. M.; Mogoantă L.; Ficai A.; Andronescu E. Biomedical Applications of Silver Nanoparticles: An Up-to-Date Overview. Nanomaterials 2018, 8 (9), 681 10.3390/nano8090681. 30200373 PMC6163202

[ref27] Gupta N.; Rai D. B.; Jangid A. K.; Kulhari H. Use of nanotechnology in antimicrobial therapy. Nanotechnology 2019, 46, 143–172. 10.1016/bs.mim.2019.04.004.

[ref28] Klębowski B.; Depciuch J.; Parlińska-Wojtan M.; Baran J. Applications of Noble Metal-Based Nanoparticles in Medicine. International Journal of Molecular Sciences 2018, 19 (12), 4031 10.3390/ijms19124031. 30551592 PMC6320918

[ref29] Lin W. Introduction: Nanoparticles in Medicine. Chem. Rev. 2015, 115 (19), 10407–10409. 10.1021/acs.chemrev.5b00534. 26463639

[ref30] Maduray K.; Parboosing R. Metal Nanoparticles: a Promising Treatment for Viral and Arboviral Infections. Biological Trace Element Research 2021, 199 (8), 3159–3176. 10.1007/s12011-020-02414-2. 33029761 PMC7540915

[ref31] Allahverdiyev A. M.; Kon K. V.; Abamor E. S.; Bagirova M.; Rafailovich M. Coping with antibiotic resistance: combining nanoparticles with antibiotics and other antimicrobial agents. Expert Review of Anti-infective Therapy 2011, 9 (11), 1035–1052. 10.1586/eri.11.121. 22029522

[ref32] Xu X.; Xu L.; Yuan G.; Wang Y.; Qu Y.; Zhou M. Synergistic combination of two antimicrobial agents closing each other’s mutant selection windows to prevent antimicrobial resistance. Sci. Rep. 2018, 8 (1), 7237 10.1038/s41598-018-25714-z. 29740150 PMC5940791

[ref33] Faya M.; Kalhapure R. S.; Kumalo H. M.; Waddad A. Y.; Omolo C.; Govender T. Conjugates and nano-delivery of antimicrobial peptides for enhancing therapeutic activity. Journal of Drug Delivery Science and Technology 2018, 44, 153–171. 10.1016/j.jddst.2017.12.010.

[ref34] Pemovska T.; Bigenzahn J. W.; Superti-Furga G. Recent advances in combinatorial drug screening and synergy scoring. Current Opinion in Pharmacology 2018, 42, 102–110. 10.1016/j.coph.2018.07.008. 30193150 PMC6219891

[ref35] Riaz Ahmed K. B.; Nagy A. M.; Brown R. P.; Zhang Q.; Malghan S. G.; Goering P. L. Silver nanoparticles: Significance of physicochemical properties and assay interference on the interpretation of in vitro cytotoxicity studies. Toxicology in Vitro 2017, 38, 179–192. 10.1016/j.tiv.2016.10.012. 27816503

[ref36] Shilo M.; Sharon A.; Baranes K.; Motiei M.; Lellouche J.-P. M.; Popovtzer R. The effect of nanoparticle size on the probability to cross the blood-brain barrier: an in-vitro endothelial cell model. J. Nanobiotechnol. 2015, 13 (1), 19 10.1186/s12951-015-0075-7. PMC4359781 25880565

[ref37] Ceña V.; Játiva P. Nanoparticle crossing of blood–brain barrier: a road to new therapeutic approaches to central nervous system diseases. Nanomedicine 2018, 13 (13), 1513–1516. 10.2217/nnm-2018-0139. 29998779

[ref38] Ha M. K.; Shim Y. J.; Yoon T. H. Effects of agglomeration on in vitro dosimetry and cellular association of silver nanoparticles. Environmental Science: Nano 2018, 5 (2), 446–455. 10.1039/C7EN00965H.

[ref39] Fröhlich E. The role of surface charge in cellular uptake and cytotoxicity of medical nanoparticles. Int. J. Nanomed. 2012, 7, 5577–91. 10.2147/IJN.S36111. PMC3493258 23144561

[ref40] Sun H.; Jiang C.; Wu L.; Bai X.; Zhai S. Cytotoxicity-Related Bioeffects Induced by Nanoparticles: The Role of Surface Chemistry. Frontiers in Bioengineering and Biotechnology 2019, 7, 1 10.3389/fbioe.2019.00414. 31921818 PMC6920110

[ref41] Gahlawat G.; Choudhury A. R. A review on the biosynthesis of metal and metal salt nanoparticles by microbes. RSC Adv. 2019, 9 (23), 12944–12967. 10.1039/C8RA10483B. 35520790 PMC9064032

[ref42] Pareek V.; Gupta R.; Panwar J. Do physico-chemical properties of silver nanoparticles decide their interaction with biological media and bactericidal action? A review. Materials Science and Engineering: C 2018, 90, 739–749. 10.1016/j.msec.2018.04.093. 29853145

[ref43] El Badawy A. M.; Silva R. G.; Morris B.; Scheckel K. G.; Suidan M. T.; Tolaymat T. M. Surface Charge-Dependent Toxicity of Silver Nanoparticles. Environ. Sci. Technol. 2011, 45 (1), 283–287. 10.1021/es1034188. 21133412

[ref44] Cui L.; Wang X.; Sun B.; Xia T.; Hu S. Predictive Metabolomic Signatures for Safety Assessment of Metal Oxide Nanoparticles. ACS Nano 2019, 13 (11), 13065–13082. 10.1021/acsnano.9b05793. 31682760

[ref45] Badawy A. M. E.; Luxton T. P.; Silva R. G.; Scheckel K. G.; Suidan M. T.; Tolaymat T. M. Impact of Environmental Conditions (pH, Ionic Strength, and Electrolyte Type) on the Surface Charge and Aggregation of Silver Nanoparticles Suspensions. Environ. Sci. Technol. 2010, 44 (4), 1260–1266. 10.1021/es902240k. 20099802

[ref46] Dehghanizade S.; Arasteh J.; Mirzaie A. Green synthesis of silver nanoparticles using Anthemis atropatana extract: characterization and in vitro biological activities. Artificial Cells, Nanomedicine, and Biotechnology 2018, 46 (1), 160–168. 10.1080/21691401.2017.1304402. 28368661

[ref47] Wei S.; Wang Y.; Tang Z.; Hu J.; Su R.; Lin J.; Zhou T.; Guo H.; Wang N.; Xu R. A size-controlled green synthesis of silver nanoparticles by using the berry extract of Sea Buckthorn and their biological activities. New J. Chem. 2020, 44 (22), 9304–9312. 10.1039/D0NJ01335H.

[ref48] Shamaila S.; Zafar N.; Riaz S.; Sharif R.; Nazir J.; Naseem S. Gold Nanoparticles: An Efficient Antimicrobial Agent against Enteric Bacterial Human Pathogen. Nanomaterials 2016, 6 (4), 71 10.3390/nano6040071. 28335198 PMC5302575

[ref49] Zhang Y.; Shareena Dasari T. P.; Deng H.; Yu H. Antimicrobial Activity of Gold Nanoparticles and Ionic Gold. Journal of Environmental Science and Health, Part C 2015, 33 (3), 286–327. 10.1080/10590501.2015.1055161. 26072980

[ref50] Al-Hakkani M. F. Biogenic copper nanoparticles and their applications: A review. SN Applied Sciences 2020, 2 (3), 505 10.1007/s42452-020-2279-1.

[ref51] Gawande M. B.; Goswami A.; Felpin F.-X.; Asefa T.; Huang X.; Silva R.; Zou X.; Zboril R.; Varma R. S. Cu and Cu-Based Nanoparticles: Synthesis and Applications in Catalysis. Chem. Rev. 2016, 116 (6), 3722–3811. 10.1021/acs.chemrev.5b00482. 26935812

[ref52] Huber D. L. Synthesis, Properties, and Applications of Iron Nanoparticles. Small 2005, 1 (5), 482–501. 10.1002/smll.200500006. 17193474

[ref53] Cotin G.; Piant S.; Mertz D.; Felder-Flesch D.; Begin-Colin S. Iron Oxide Nanoparticles for Biomedical Applications: Synthesis, Functionalization, and Application. Iron Oxide Nanoparticles for Biomedical Applications 2018, 43–88. 10.1016/B978-0-08-101925-2.00002-4.

[ref54] Arakha M.; Pal S.; Samantarrai D.; Panigrahi T. K.; Mallick B. C.; Pramanik K.; Mallick B.; Jha S. Antimicrobial activity of iron oxide nanoparticle upon modulation of nanoparticle-bacteria interface. Sci. Rep. 2015, 5 (1), 14813 10.1038/srep14813. 26437582 PMC4594095

[ref55] Siddiqi K. S.; ur Rahman A.; Tajuddin; Husen A. Properties of Zinc Oxide Nanoparticles and Their Activity Against Microbes. Nanoscale Res. Lett. 2018, 13 (1), 141 10.1186/s11671-018-2532-3. 29740719 PMC5940970

[ref56] Król A.; Pomastowski P.; Rafińska K.; Railean-Plugaru V.; Buszewski B. Zinc oxide nanoparticles: Synthesis, antiseptic activity and toxicity mechanism. Adv. Colloid Interface Sci. 2017, 249, 37–52. 10.1016/j.cis.2017.07.033. 28923702

[ref57] Hassanpour P.; Panahi Y.; Ebrahimi-Kalan A.; Akbarzadeh A.; Davaran S.; Nasibova A. N.; Khalilov R.; Kavetskyy T. Biomedical applications of aluminium oxide nanoparticles. Micro & Nano Letters 2018, 13 (9), 1227–1231. 10.1049/mnl.2018.5070.

[ref58] McNamara K.; Tofail S. A. M. Nanoparticles in biomedical applications. Advances in Physics: X 2017, 2 (1), 54–88. 10.1080/23746149.2016.1254570. 26024211

[ref59] Sundrarajan M.; Bama K.; Bhavani M.; Jegatheeswaran S.; Ambika S.; Sangili A.; Nithya P.; Sumathi R. Obtaining titanium dioxide nanoparticles with spherical shape and antimicrobial properties using M. citrifolia leaves extract by hydrothermal method. Journal of Photochemistry and Photobiology B: Biology 2017, 171, 117–124. 10.1016/j.jphotobiol.2017.05.003. 28501689

[ref60] Pedone D.; Moglianetti M.; De Luca E.; Bardi G.; Pompa P. P. Platinum nanoparticles in nanobiomedicine. Chem. Soc. Rev. 2017, 46 (16), 4951–4975. 10.1039/C7CS00152E. 28696452

[ref61] Li Y.; Yun K.-H.; Lee H.; Goh S.-H.; Suh Y.-G.; Choi Y. Porous platinum nanoparticles as a high-Z and oxygen generating nanozyme for enhanced radiotherapy in vivo. Biomaterials 2019, 197, 12–19. 10.1016/j.biomaterials.2019.01.004. 30623793

[ref62] Phan T. T. V.; Huynh T.-C.; Manivasagan P.; Mondal S.; Oh J. An Up-To-Date Review on Biomedical Applications of Palladium Nanoparticles. Nanomaterials 2020, 10 (1), 66 10.3390/nano10010066. PMC7023275 31892149

[ref63] Leso V.; Iavicoli I. Palladium Nanoparticles: Toxicological Effects and Potential Implications for Occupational Risk Assessment. International Journal of Molecular Sciences 2018, 19 (2), 503 10.3390/ijms19020503. 29414923 PMC5855725

[ref64] Calderón-Jiménez B.; Johnson M. E.; Montoro Bustos A. R.; Murphy K. E.; Winchester M. R.; Vega Baudrit J. R. Silver Nanoparticles: Technological Advances, Societal Impacts, and Metrological Challenges. Frontiers in Chemistry 2017, 5, 1 10.3389/fchem.2017.00006. 28271059 PMC5318410

[ref65] Tao C. Antimicrobial activity and toxicity of gold nanoparticles: research progress, challenges and prospects. Letters in Applied Microbiology 2018, 67 (6), 537–543. 10.1111/lam.13082. 30269338

[ref66] Chen W.-Y.; Chang H.-Y.; Lu J.-K.; Huang Y.-C.; Harroun S. G.; Tseng Y.-T.; Li Y.-J.; Huang C.-C.; Chang H.-T. Self-Assembly of Antimicrobial Peptides on Gold Nanodots: Against Multidrug-Resistant Bacteria and Wound-Healing Application. Adv. Funct. Mater. 2015, 25 (46), 7189–7199. 10.1002/adfm.201503248.

[ref67] Mehravani B.; Ribeiro A. I.; Zille A. Gold Nanoparticles Synthesis and Antimicrobial Effect on Fibrous Materials. Nanomaterials 2021, 11 (5), 1067 10.3390/nano11051067. 33919401 PMC8143294

[ref68] Malhotra N.; Lee J.-S.; Liman R. A. D.; Ruallo J. M. S.; Villaflores O. B.; Ger T.-R.; Hsiao C.-D. Potential Toxicity of Iron Oxide Magnetic Nanoparticles: A Review. Molecules 2020, 25 (14), 3159 10.3390/molecules25143159. 32664325 PMC7397295

[ref69] Dizaj S. M.; Lotfipour F.; Barzegar-Jalali M.; Zarrintan M. H.; Adibkia K. Antimicrobial activity of the metals and metal oxide nanoparticles. Materials Science and Engineering: C 2014, 44, 278–284. 10.1016/j.msec.2014.08.031. 25280707

[ref70] de Dicastillo C. L.; Patiño C.; Galotto M. J.; Vásquez-Martínez Y.; Torrent C.; Alburquenque D.; Pereira A.; Escrig J. Novel hollow titanium dioxide nanospheres with antimicrobial activity against resistant bacteria. Beilstein Journal of Nanotechnology 2019, 10, 1716–1725. 10.3762/bjnano.10.167. 31501743 PMC6720579

[ref71] Visai L.; De Nardo L.; Punta C.; Melone L.; Cigada A.; Imbriani M.; Arciola C. R. Titanium Oxide Antibacterial Surfaces in Biomedical Devices. International Journal of Artificial Organs 2011, 34 (9), 929–946. 10.5301/ijao.5000050. 22094576

[ref72] Soo J. Z.; Chai L. C.; Ang B. C.; Ong B. H. Enhancing the Antibacterial Performance of Titanium Dioxide Nanofibers by Coating with Silver Nanoparticles. ACS Applied Nano Materials 2020, 3 (6), 5743–5751. 10.1021/acsanm.0c00925.

[ref73] Krause B. C.; Kriegel F. L.; Rosenkranz D.; Dreiack N.; Tentschert J.; Jungnickel H.; Jalili P.; Fessard V.; Laux P.; Luch A. Aluminum and aluminum oxide nanomaterials uptake after oral exposure - a comparative study. Sci. Rep. 2020, 10 (1), 2698 10.1038/s41598-020-59710-z. 32060369 PMC7021764

[ref74] Some S.; Kumar Sen I.; Mandal A.; Aslan T.; Ustun Y.; Yilmaz E. Ş.; Katı A.; Demirbas A.; Mandal A. K.; Ocsoy I. Biosynthesis of silver nanoparticles and their versatile antimicrobial properties. Materials Research Express 2019, 6 (1), 012001 10.1088/2053-1591/aae23e.

[ref75] Jeevanandam J.; Barhoum A.; Chan Y. S.; Dufresne A.; Danquah M. K. Review on nanoparticles and nanostructured materials: history, sources, toxicity and regulations. Beilstein Journal of Nanotechnology 2018, 9, 1050–1074. 10.3762/bjnano.9.98. 29719757 PMC5905289

[ref76] Ribeiro A. I.; Modic M.; Cvelbar U.; Dinescu G.; Mitu B.; Nikiforov A.; Leys C.; Kuchakova I.; De Vrieze M.; Felgueiras H. P.; Souto A. P.; Zille A. Effect of Dispersion Solvent on the Deposition of PVP-Silver Nanoparticles onto DBD Plasma-Treated Polyamide 6,6 Fabric and Its Antimicrobial Efficiency. Nanomaterials 2020, 10 (4), 607 10.3390/nano10040607. 32224934 PMC7221693

[ref77] Amini S. M. Preparation of antimicrobial metallic nanoparticles with bioactive compounds. Materials Science and Engineering: C 2019, 103, 109809 10.1016/j.msec.2019.109809. 31349497

[ref78] Ali J.; Ali N.; Wang L.; Waseem H.; Pan G. Revisiting the mechanistic pathways for bacterial mediated synthesis of noble metal nanoparticles. J. Microbiol. Methods 2019, 159, 18–25. 10.1016/j.mimet.2019.02.010. 30797020

[ref79] Rana A.; Yadav K.; Jagadevan S. A comprehensive review on green synthesis of nature-inspired metal nanoparticles: Mechanism, application and toxicity. Journal of Cleaner Production 2020, 272, 122880 10.1016/j.jclepro.2020.122880.

[ref80] Dauthal P.; Mukhopadhyay M. Noble Metal Nanoparticles: Plant-Mediated Synthesis, Mechanistic Aspects of Synthesis, and Applications. Ind. Eng. Chem. Res. 2016, 55 (36), 9557–9577. 10.1021/acs.iecr.6b00861.

[ref81] Neouze M.-A.; Schubert U. Surface Modification and Functionalization of Metal and Metal Oxide Nanoparticles by Organic Ligands. Monatshefte für Chemie - Chemical Monthly 2008, 139 (3), 183–195. 10.1007/s00706-007-0775-2.

[ref82] Hu P.; Chen L.; Kang X.; Chen S. Surface Functionalization of Metal Nanoparticles by Conjugated Metal–Ligand Interfacial Bonds: Impacts on Intraparticle Charge Transfer. Acc. Chem. Res. 2016, 49 (10), 2251–2260. 10.1021/acs.accounts.6b00377. 27690382

[ref83] Hur Y. E.; Kim S.; Kim J.-H.; Cha S.-H.; Choi M.-J.; Cho S.; Park Y. One-step functionalization of gold and silver nanoparticles by ampicillin. Mater. Lett. 2014, 129, 185–190. 10.1016/j.matlet.2014.05.032.

[ref84] Doern C. D. When Does 2 Plus 2 Equal 5? A Review of Antimicrobial Synergy Testing. Journal of Clinical Microbiology 2014, 52 (12), 4124–4128. 10.1128/JCM.01121-14. 24920779 PMC4313275

[ref85] Laxminarayan R.; Van Boeckel T.; Frost I.; Kariuki S.; Khan E. A.; Limmathurotsakul D.; Larsson D. G. J.; Levy-Hara G.; Mendelson M.; Outterson K.; Peacock S. J.; Zhu Y.-G. The Lancet Infectious Diseases Commission on antimicrobial resistance: 6 years later. Lancet Infectious Diseases 2020, 20 (4), e51–e60. 10.1016/S1473-3099(20)30003-7. 32059790

[ref86] Liu J.; Gefen O.; Ronin I.; Bar-Meir M.; Balaban N. Q. Effect of tolerance on the evolution of antibiotic resistance under drug combinations. Science 2020, 367 (6474), 200–204. 10.1126/science.aay3041. 31919223

[ref87] Hwang I.-s.; Hwang J. H.; Choi H.; Kim K.-J.; Lee D. G. Synergistic effects between silver nanoparticles and antibiotics and the mechanisms involved. Journal of Medical Microbiology 2012, 61 (12), 1719–1726. 10.1099/jmm.0.047100-0. 22956753

[ref88] Wan G.; Ruan L.; Yin Y.; Yang T.; Ge M.; Cheng X. Effects of silver nanoparticles in combination with antibiotics on the resistant bacteria Acinetobacter baumannii. Int. J. Nanomed. 2016, 11, 3789–3800. 10.2147/IJN.S104166. PMC4990392 27574420

[ref89] Smekalova M.; Aragon V.; Panacek A.; Prucek R.; Zboril R.; Kvitek L. Enhanced antibacterial effect of antibiotics in combination with silver nanoparticles against animal pathogens. Veterinary Journal 2016, 209, 174–179. 10.1016/j.tvjl.2015.10.032. 26832810

[ref90] Lopez-Carrizales M.; Velasco K.; Castillo C.; Flores A.; Magaña M.; Martinez-Castanon G.; Martinez-Gutierrez F. In Vitro Synergism of Silver Nanoparticles with Antibiotics as an Alternative Treatment in Multiresistant Uropathogens. Antibiotics 2018, 7 (2), 50 10.3390/antibiotics7020050. 29921822 PMC6023009

[ref91] Salarian A. A.; Bahari Mollamahale Y.; Hami Z.; Soltani-Rezaee-Rad M. Cephalexin nanoparticles: Synthesis, cytotoxicity and their synergistic antibacterial study in combination with silver nanoparticles. Mater. Chem. Phys. 2017, 198, 125–130. 10.1016/j.matchemphys.2017.05.059.

[ref92] Kora A. J.; Rastogi L. Enhancement of Antibacterial Activity of Capped Silver Nanoparticles in Combination with Antibiotics, on Model Gram-Negative and Gram-Positive Bacteria. Bioinorganic Chemistry and Applications 2013, 2013, 1–7. 10.1155/2013/871097. PMC3732601 23970844

[ref93] Rogowska A.; Rafińska K.; Pomastowski P.; Walczak J.; Railean-Plugaru V.; Buszewska-Forajta M.; Buszewski B. Silver nanoparticles functionalized with ampicillin. Electrophoresis 2017, 38 (21), 2757–2764. 10.1002/elps.201700093. 28704596

[ref94] Li P.; Li J.; Wu C.; Wu Q.; Li J. Synergistic antibacterial effects of β-lactam antibiotic combined with silver nanoparticles. Nanotechnology 2005, 16 (9), 1912–1917. 10.1088/0957-4484/16/9/082.

[ref95] Kaur A.; Kumar R. Enhanced bactericidal efficacy of polymer stabilized silver nanoparticles in conjugation with different classes of antibiotics. RSC Adv. 2019, 9 (2), 1095–1105. 10.1039/C8RA07980C. 35517620 PMC9059492

[ref96] Mazur P.; Skiba-Kurek I.; Mrowiec P.; Karczewska E.; Drożdż R. Synergistic ROS-Associated Antimicrobial Activity of Silver Nanoparticles and Gentamicin Against Staphylococcus epidermidis. Int. J. Nanomed. 2020, 15, 3551–3562. 10.2147/IJN.S246484. PMC7246328 32547013

[ref97] McShan D.; Zhang Y.; Deng H.; Ray P. C.; Yu H. Synergistic Antibacterial Effect of Silver Nanoparticles Combined with Ineffective Antibiotics on Drug ResistantSalmonella typhimuriumDT104. Journal of Environmental Science and Health, Part C 2015, 33 (3), 369–384. 10.1080/10590501.2015.1055165. 26072671

[ref98] Wang Y.-W.; Tang H.; Wu D.; Liu D.; Liu Y.; Cao A.; Wang H. Enhanced bactericidal toxicity of silver nanoparticles by the antibiotic gentamicin. Environmental Science: Nano 2016, 3 (4), 788–798. 10.1039/C6EN00031B.

[ref99] Kaur A.; Preet S.; Kumar V.; Kumar R.; Kumar R. Synergetic effect of vancomycin loaded silver nanoparticles for enhanced antibacterial activity. Colloids Surf., B 2019, 176, 62–69. 10.1016/j.colsurfb.2018.12.043. 30594704

[ref100] Ipe D. S.; Kumar P. T. S.; Love R. M.; Hamlet S. M. Silver Nanoparticles at Biocompatible Dosage Synergistically Increases Bacterial Susceptibility to Antibiotics. Frontiers in Microbiology 2020, 11, 1 10.3389/fmicb.2020.01074. 32670214 PMC7326045

[ref101] Panáček A.; Smékalová M.; Kilianová M.; Prucek R.; Bogdanová K.; Večeřová R.; Kolář M.; Havrdová M.; Płaza G.; Chojniak J.; Zbořil R.; Kvítek L. Strong and Nonspecific Synergistic Antibacterial Efficiency of Antibiotics Combined with Silver Nanoparticles at Very Low Concentrations Showing No Cytotoxic Effect. Molecules 2016, 21 (1), 26 10.3390/molecules21010026. PMC6273824 26729075

[ref102] Panáček A.; Smékalová M.; Večeřová R.; Bogdanová K.; Röderová M.; Kolář M.; Kilianová M.; Hradilová Š.; Froning J. P.; Havrdová M.; Prucek R.; Zbořil R.; Kvítek L. Silver nanoparticles strongly enhance and restore bactericidal activity of inactive antibiotics against multiresistant Enterobacteriaceae. Colloids Surf., B 2016, 142, 392–399. 10.1016/j.colsurfb.2016.03.007. 26970828

[ref103] Deng H.; McShan D.; Zhang Y.; Sinha S. S.; Arslan Z.; Ray P. C.; Yu H. Mechanistic Study of the Synergistic Antibacterial Activity of Combined Silver Nanoparticles and Common Antibiotics. Environ. Sci. Technol. 2016, 50 (16), 8840–8848. 10.1021/acs.est.6b00998. 27390928 PMC5300770

[ref104] Tom R. T.; Suryanarayanan V.; Reddy P. G.; Baskaran S.; Pradeep T. Ciprofloxacin-Protected Gold Nanoparticles. Langmuir 2004, 20 (5), 1909–1914. 10.1021/la0358567. 15801462

[ref105] Eleftheriadou I.; Giannousi K.; Protonotariou E.; Skoura L.; Arsenakis M.; Dendrinou-Samara C.; Sivropoulou A. Cocktail of CuO, ZnO, or CuZn Nanoparticles and Antibiotics for Combating Multidrug-Resistant Pseudomonas aeruginosa via Efflux Pump Inhibition. ACS Applied Nano Materials 2021, 4 (9), 9799–9810. 10.1021/acsanm.1c02208.

[ref106] Vernaya O. I.; Shabatin V. P.; Semenov A. M.; Shabatina T. I.; Melnikov M. Y. Low-Temperature Synthesis and Antibacterial Activity of Hybrid Systems of Gentamicin Sulfate with Copper and Iron Nanoparticles. Moscow University Chemistry Bulletin 2020, 75 (4), 258–260. 10.3103/S0027131420040094.

[ref107] Bhande R. M.; Khobragade C. N.; Mane R. S.; Bhande S. Enhanced synergism of antibiotics with zinc oxide nanoparticles against extended spectrum β-lactamase producers implicated in urinary tract infections. J. Nanopart. Res. 2013, 15 (1), 1413 10.1007/s11051-012-1413-4.

[ref108] Rath G.; Hussain T.; Chauhan G.; Garg T.; Goyal A. K. Development and characterization of cefazolin loaded zinc oxide nanoparticles composite gelatin nanofiber mats for postoperative surgical wounds. Materials Science and Engineering: C 2016, 58, 242–253. 10.1016/j.msec.2015.08.050. 26478308

[ref109] Chandrasekaran K.; Varaprasad K.; Venugopal S. K.; Arun L.; Hameed A. S. H. Synergistic Antibacterial Effect of the Magnesium-Doped ZnO Nanoparticles with Chloramphenicol. BioNanoScience 2020, 10 (1), 106–111. 10.1007/s12668-019-00696-y.

[ref110] Fakhri A.; Tahami S.; Naji M. Synthesis and characterization of core-shell bimetallic nanoparticles for synergistic antimicrobial effect studies in combination with doxycycline on burn specific pathogens. Journal of Photochemistry and Photobiology B: Biology 2017, 169, 21–26. 10.1016/j.jphotobiol.2017.02.014. 28254569

[ref111] Banoee M.; Seif S.; Nazari Z. E.; Jafari-Fesharaki P.; Shahverdi H. R.; Moballegh A.; Moghaddam K. M.; Shahverdi A. R. ZnO nanoparticles enhanced antibacterial activity of ciprofloxacin against Staphylococcus aureus and Escherichia coli. Journal of Biomedical Materials Research Part B: Applied Biomaterials 2010, 93B (2), 557–561. 10.1002/jbm.b.31615. 20225250

[ref112] Wypij M.; Świecimska M.; Czarnecka J.; Dahm H.; Rai M.; Golinska P. Antimicrobial and cytotoxic activity of silver nanoparticles synthesized from two haloalkaliphilic actinobacterial strains alone and in combination with antibiotics. J. Appl. Microbiol. 2018, 124 (6), 1411–1424. 10.1111/jam.13723. 29427473

[ref113] Shahverdi A. R.; Fakhimi A.; Shahverdi H. R.; Minaian S. Synthesis and effect of silver nanoparticles on the antibacterial activity of different antibiotics against Staphylococcus aureus and Escherichia coli. Nanomedicine: Nanotechnology, Biology and Medicine 2007, 3 (2), 168–171. 10.1016/j.nano.2007.02.001. 17468052

[ref114] Katva S.; Das S.; Moti H. S.; Jyoti A.; Kaushik S. Antibacterial Synergy of Silver Nanoparticles with Gentamicin and Chloramphenicol against Enterococcus faecalis. Pharmacognosy Magazine 2018, 13 (Suppl 4), S828–S833. 29491640 10.4103/pm.pm_120_17 PMC5822507

[ref115] Naik M. M.; Prabhu M. S.; Samant S. N.; Naik P. M.; Shirodkar S. Synergistic Action of Silver Nanoparticles Synthesized from Silver Resistant Estuarine Pseudomonas aeruginosa Strain SN5 with Antibiotics against Antibiotic Resistant Bacterial Human Pathogens. Thalassas: An International Journal of Marine Sciences 2017, 33 (1), 73–80. 10.1007/s41208-017-0023-4.

[ref116] Baker S.; Pasha A.; Satish S. Biogenic nanoparticles bearing antibacterial activity and their synergistic effect with broad spectrum antibiotics: Emerging strategy to combat drug resistant pathogens. Saudi Pharmaceutical Journal 2017, 25 (1), 44–51. 10.1016/j.jsps.2015.06.011. 28223861 PMC5310154

[ref117] Naqvi S. Z.; Kiran U.; Ali; Jamal; Hameed; Ahmed; Ali Combined efficacy of biologically synthesized silver nanoparticles and different antibiotics against multidrug-resistant bacteria. Int. J. Nanomed. 2013, 8, 3187–95. 10.2147/IJN.S49284. PMC3754765 23986635

[ref118] Fayaz A. M.; Balaji K.; Girilal M.; Yadav R.; Kalaichelvan P. T.; Venketesan R. Biogenic synthesis of silver nanoparticles and their synergistic effect with antibiotics: a study against gram-positive and gram-negative bacteria. Nanomedicine: Nanotechnology, Biology and Medicine 2010, 6 (1), 103–109. 10.1016/j.nano.2009.04.006. 19447203

[ref119] Chopade B. A.; Singh R.; Wagh P.; Wadhwani S.; Gaidhani S.; Kumbhar A.; Bellare J. Synthesis, optimization, and characterization of silver nanoparticles from Acinetobacter calcoaceticus and their enhanced antibacterial activity when combined with antibiotics. Int. J. Nanomed. 2013, 8, 4277–4290. 10.2147/IJN.S48913. PMC3826770 24235826

[ref120] Dar M. A.; Ingle A.; Rai M. Enhanced antimicrobial activity of silver nanoparticles synthesized by Cryphonectria sp. evaluated singly and in combination with antibiotics. Nanomedicine: Nanotechnology, Biology and Medicine 2013, 9 (1), 105–110. 10.1016/j.nano.2012.04.007. 22633901

[ref121] Barapatre A.; Aadil K. R.; Jha H. Synergistic antibacterial and antibiofilm activity of silver nanoparticles biosynthesized by lignin-degrading fungus. Bioresources and Bioprocessing 2016, 3 (1), 8 10.1186/s40643-016-0083-y.

[ref122] Ranpariya B.; Salunke G.; Karmakar S.; Babiya K.; Sutar S.; Kadoo N.; Kumbhakar P.; Ghosh S. Antimicrobial Synergy of Silver-Platinum Nanohybrids With Antibiotics. Frontiers in Microbiology 2021, 11, 1 10.3389/fmicb.2020.610968. PMC7882503 33597929

[ref123] Chopade B.; Ghosh; Patil; Ahire; Kitture; Jabgunde; Kale; Pardesi; Cameotra; Bellare; Dhavale Synthesis of silver nanoparticles using Dioscorea bulbifera tuber extract and evaluation of its synergistic potential in combination with antimicrobial agents. Int. J. Nanomed. 2012, 7, 483–96. 10.2147/IJN.S24793. PMC3273981 22334779

[ref124] Saratale G. D.; Saratale R. G.; Benelli G.; Kumar G.; Pugazhendhi A.; Kim D.-S.; Shin H.-S. Anti-diabetic Potential of Silver Nanoparticles Synthesized with Argyreia nervosa Leaf Extract High Synergistic Antibacterial Activity with Standard Antibiotics Against Foodborne Bacteria. Journal of Cluster Science 2017, 28 (3), 1709–1727. 10.1007/s10876-017-1179-z.

[ref125] Rastogi L.; Kora A. J.; Sashidhar R. B. Antibacterial effects of gum kondagogu reduced/stabilized silver nanoparticles in combination with various antibiotics: a mechanistic approach. Applied Nanoscience 2015, 5 (5), 535–543. 10.1007/s13204-014-0347-9.

[ref126] Halawani E. M.; Hassan A. M.; Gad El-Rab S. M. F. Nanoformulation of Biogenic Cefotaxime-Conjugated-Silver Nanoparticles for Enhanced Antibacterial Efficacy Against Multidrug-Resistant Bacteria and Anticancer Studies. Int. J. Nanomed. 2020, 15, 1889–1901. 10.2147/IJN.S236182. PMC7090159 32256066

[ref127] Abo-Shama U. H.; El-Gendy H.; Mousa W. S.; Hamouda R. A.; Yousuf W. E.; Hetta H. F.; Abdeen E. E. Synergistic and Antagonistic Effects of Metal Nanoparticles in Combination with Antibiotics Against Some Reference Strains of Pathogenic Microorganisms. Infection and Drug Resistance 2020, 13, 351–362. 10.2147/IDR.S234425. 32104007 PMC7012269

[ref128] Lin P.; Wang F.-Q.; Li C.-T.; Yan Z.-F. An Enhancement of Antibacterial Activity and Synergistic Effect of Biosynthesized Silver Nanoparticles by Eurotium cristatum with Various Antibiotics. Biotechnology and Bioprocess Engineering 2020, 25 (3), 450–458. 10.1007/s12257-019-0506-7.

[ref129] Jyoti K.; Baunthiyal M.; Singh A. Characterization of silver nanoparticles synthesized using Urtica dioica Linn. leaves and their synergistic effects with antibiotics. Journal of Radiation Research and Applied Sciences 2016, 9 (3), 217–227. 10.1016/j.jrras.2015.10.002.

[ref130] Patra J. K.; Baek K.-H. Antibacterial Activity and Synergistic Antibacterial Potential of Biosynthesized Silver Nanoparticles against Foodborne Pathogenic Bacteria along with its Anticandidal and Antioxidant Effects. Frontiers in Microbiology 2017, 08, 1 10.3389/fmicb.2017.00167. PMC5309230 28261161

[ref131] Yaqub A.; Malkani N.; Shabbir A.; Ditta S. A.; Tanvir F.; Ali S.; Naz M.; Kazmi S. A. R.; Ullah R. Novel Biosynthesis of Copper Nanoparticles Using Zingiber and Allium sp. with Synergic Effect of Doxycycline for Anticancer and Bactericidal Activity. Curr. Microbiol. 2020, 77 (9), 2287–2299. 10.1007/s00284-020-02058-4. 32535649

[ref132] Arul Selvaraj R. C.; Rajendran M.; Nagaiah H. P. Re-Potentiation of β-Lactam Antibiotic by Synergistic Combination with Biogenic Copper Oxide Nanocubes against Biofilm Forming Multidrug-Resistant Bacteria. Molecules 2019, 24 (17), 3055 10.3390/molecules24173055. 31443467 PMC6749510

[ref133] Ehsan S.; Sajjad M. Bioinspired Synthesis of Zinc Oxide Nanoparticle and its Combined Efficacy with Different Antibiotics against Multidrug Resistant Bacteria. Journal of Biomaterials and Nanobiotechnology 2017, 08 (02), 159–175. 10.4236/jbnb.2017.82011.

[ref134] MadhumitaGhosh; Nallal V. U.; Prabha K.; Muthupandi S.; Razia M. Synergistic antibacterial potential of plant-based Zinc oxide Nanoparticles in combination with antibiotics against Pseudomonas aeruginosa. Materials Today: Proceedings 2022, 49 (7), 2632–2635. 10.1016/j.matpr.2021.08.046.

[ref135] Sabir D. Synergistic Effect of Silver Nanoparticles Combined with Different Antibiotics against Multidrug-Resistant Acinetobacter Baumannii Strain H72721. 3nd International Conference of Natural Science 2018-Biotechnology 2018, 7–11. 10.31530/17028.

[ref136] Malawong S.; Thammawithan S.; Sirithongsuk P.; Daduang S.; Klaynongsruang S.; Wong P. T.; Patramanon R. Silver Nanoparticles Enhance Antimicrobial Efficacy of Antibiotics and Restore That Efficacy against the Melioidosis Pathogen. Antibiotics 2021, 10 (7), 839 10.3390/antibiotics10070839. 34356761 PMC8300767

[ref137] Vazquez-Munoz R.; Meza-Villezcas A.; Fournier P. G. J.; Soria-Castro E.; Juarez-Moreno K.; Gallego-Hernandez A. L.; Bogdanchikova N.; Vazquez-Duhalt R.; Huerta-Saquero A. Enhancement of antibiotics antimicrobial activity due to the silver nanoparticles impact on the cell membrane. PLoS One 2019, 14 (11), e0224904 10.1371/journal.pone.0224904. 31703098 PMC6839893

[ref138] Surwade P.; Ghildyal C.; Weikel C.; Luxton T.; Peloquin D.; Fan X.; Shah V. Augmented antibacterial activity of ampicillin with silver nanoparticles against methicillin-resistant Staphylococcus aureus (MRSA). Journal of Antibiotics 2019, 72 (1), 50–53. 10.1038/s41429-018-0111-6. 30361634 PMC7372723

[ref139] Ghasemi F.; Jalal R. Antimicrobial action of zinc oxide nanoparticles in combination with ciprofloxacin and ceftazidime against multidrug-resistant Acinetobacter baumannii. Journal of Global Antimicrobial Resistance 2016, 6, 118–122. 10.1016/j.jgar.2016.04.007. 27530853

[ref140] MadhumitaGhosh; Nallal V. U.; Prabha K.; Muthupandi S.; Razia M. Synergistic antibacterial potential of plant-based Zinc oxide Nanoparticles in combination with antibiotics against Pseudomonas aeruginosa. Materials Today: Proceedings 2022, 49, 2632–2635. 10.1016/j.matpr.2021.08.046.

[ref141] Saha B.; Bhattacharya J.; Mukherjee A.; Ghosh A. K.; Santra C. R.; Dasgupta A. K.; Karmakar P. In Vitro Structural and Functional Evaluation of Gold Nanoparticles Conjugated Antibiotics. Nanoscale Res. Lett. 2007, 2 (12), 614–622. 10.1007/s11671-007-9104-2.

[ref142] Ma K.; Dong P.; Liang M.; Yu S.; Chen Y.; Wang F. Facile Assembly of Multifunctional Antibacterial Nanoplatform Leveraging Synergistic Sensitization between Silver Nanostructure and Vancomycin. ACS Appl. Mater. Interfaces 2020, 12 (6), 6955–6965. 10.1021/acsami.9b22043. 31977179

[ref143] Murei A.; Ayinde W. B.; Gitari M. W.; Samie A. Functionalization and antimicrobial evaluation of ampicillin, penicillin and vancomycin with Pyrenacantha grandiflora Baill and silver nanoparticles. Sci. Rep. 2020, 10 (1), 11596 10.1038/s41598-020-68290-x. 32665625 PMC7360584

[ref144] Ganesh M.; Aziz A. S.; Ubaidulla U.; Hemalatha P.; Saravanakumar A.; Ravikumar R.; Peng M. M.; Choi E. Y.; Jang H. T. Sulfanilamide and silver nanoparticles-loaded polyvinyl alcohol-chitosan composite electrospun nanofibers: Synthesis and evaluation on synergism in wound healing. Journal of Industrial and Engineering Chemistry 2016, 39, 127–135. 10.1016/j.jiec.2016.05.021.

[ref145] Brown A. N.; Smith K.; Samuels T. A.; Lu J.; Obare S. O.; Scott M. E. Nanoparticles Functionalized with Ampicillin Destroy Multiple-Antibiotic-Resistant Isolates of Pseudomonas aeruginosa and Enterobacter aerogenes and Methicillin-Resistant Staphylococcus aureus. Appl. Environ. Microbiol. 2012, 78 (8), 2768–2774. 10.1128/AEM.06513-11. 22286985 PMC3318834

[ref146] Gu H.; Ho P. L.; Tong E.; Wang L.; Xu B. Presenting Vancomycin on Nanoparticles to Enhance Antimicrobial Activities. Nano Lett. 2003, 3 (9), 1261–1263. 10.1021/nl034396z.

[ref147] de Oliveira J. F. A.; Saito Â.; Bido A. T.; Kobarg J.; Stassen H. K.; Cardoso M. B. Defeating Bacterial Resistance and Preventing Mammalian Cells Toxicity Through Rational Design of Antibiotic-Functionalized Nanoparticles. Sci. Rep. 2017, 7 (1), 1326 10.1038/s41598-017-01209-1. 28465530 PMC5430956

[ref148] Wei Q.; Ji J.; Fu J.; Shen J. Norvancomycin-capped silver nanoparticles: Synthesis and antibacterial activities against E. coli. Science in China Series B: Chemistry 2007, 50 (3), 418–424. 10.1007/s11426-007-0028-6.

[ref149] Gupta A.; Saleh N. M.; Das R.; Landis R. F.; Bigdeli A.; Motamedchaboki K.; Rosa Campos A.; Pomeroy K.; Mahmoudi M.; Rotello V. M. Synergistic antimicrobial therapy using nanoparticles and antibiotics for the treatment of multidrug-resistant bacterial infection. Nano Futures 2017, 1 (1), 015004 10.1088/2399-1984/aa69fb.

[ref150] Tyagi P. K.; Gola D.; Tyagi S.; Mishra A. K.; Kumar A.; Chauhan N.; Ahuja A.; Sirohi S. Synthesis of zinc oxide nanoparticles and its conjugation with antibiotic: Antibacterial and morphological characterization. Environmental Nanotechnology, Monitoring & Management 2020, 14, 100391 10.1016/j.enmm.2020.100391.

[ref151] Mohammed Fayaz A.; Girilal M.; Mahdy S. A.; Somsundar S. S.; Venkatesan R.; Kalaichelvan P. T. Vancomycin bound biogenic gold nanoparticles: A different perspective for development of anti VRSA agents. Process Biochemistry 2011, 46 (3), 636–641. 10.1016/j.procbio.2010.11.001.

[ref152] Khatoon N.; Alam H.; Khan A.; Raza K.; Sardar M. Ampicillin Silver Nanoformulations against Multidrug resistant bacteria. Sci. Rep. 2019, 9 (1), 6848 10.1038/s41598-019-43309-0. 31048721 PMC6497658

[ref153] Rai A.; Prabhune A.; Perry C. C. Antibiotic mediated synthesis of gold nanoparticles with potent antimicrobial activity and their application in antimicrobial coatings. J. Mater. Chem. 2010, 20 (32), 6789 10.1039/c0jm00817f.

[ref154] Zou Z.; Sun J.; Li Q.; Pu Y.; Liu J.; Sun R.; Wang L.; Jiang T. Vancomycin modified copper sulfide nanoparticles for photokilling of vancomycin-resistant enterococci bacteria. Colloids Surf., B 2020, 189, 110875 10.1016/j.colsurfb.2020.110875. 32087532

[ref155] Wu Y.; Dockendorff C. Synthesis of Simplified Azasordarin Analogs as Potential Antifungal Agents. Journal of Organic Chemistry 2019, 84 (9), 5292–5304. 10.1021/acs.joc.9b00296. 30919633

[ref156] Zhu P.; Zhou L.; Song Y.; Cai L.; Ji M.; Wang J.; Ruan G.; Chen J. Encapsulating insoluble antifungal drugs into oleic acid-modified silica mesocomposites with enhanced fungicidal activity. J. Mater. Chem. B 2020, 8 (22), 4899–4907. 10.1039/D0TB00106F. 32314756

[ref157] Bhatt K.; Agolli A.; Patel M. H.; Garimella R.; Devi M.; Garcia E.; Amin H.; Domingue C.; Del Castillo R. G.; Sanchez-Gonzalez M. High mortality co-infections of COVID-19 patients: mucormycosis and other fungal infections. Discoveries 2021, 9 (1), e126 10.15190/d.2021.5. 34036149 PMC8137279

[ref158] Campoy S.; Adrio J. L. Antifungals. Biochem. Pharmacol. 2017, 133, 86–96. 10.1016/j.bcp.2016.11.019. 27884742

[ref159] Liu W.; Yuan L.; Wang S. Recent Progress in the Discovery of Antifungal Agents Targeting the Cell Wall. J. Med. Chem. 2020, 63 (21), 12429–12459. 10.1021/acs.jmedchem.0c00748. 32692166

[ref160] Gintjee T. J.; Donnelley M. A.; Thompson G. R. Aspiring Antifungals: Review of Current Antifungal Pipeline Developments. Journal of Fungi 2020, 6 (1), 28 10.3390/jof6010028. 32106450 PMC7151215

[ref161] Campitelli M.; Zeineddine N.; Samaha G.; Maslak S. Combination Antifungal Therapy: A Review of Current Data. Journal of Clinical Medicine Research 2017, 9 (6), 451–456. 10.14740/jocmr2992w. 28496543 PMC5412516

[ref162] Zainab S.; Hamid S.; Sahar S.; Ali N. Fluconazole and biogenic silver nanoparticles-based nano-fungicidal system for highly efficient elimination of multi-drug resistant Candida biofilms. Mater. Chem. Phys. 2022, 276, 125451 10.1016/j.matchemphys.2021.125451.

[ref163] Kumar C. G.; Poornachandra Y. Biodirected synthesis of Miconazole-conjugated bacterial silver nanoparticles and their application as antifungal agents and drug delivery vehicles. Colloids Surf., B 2015, 125, 110–119. 10.1016/j.colsurfb.2014.11.025. 25460601

[ref164] Halbandge S. D.; Mortale S. P.; Karuppayil S. M. Biofabricated Silver Nanoparticles Synergistically Activate Amphotericin B Against Mature Biofilm Forms of Candida Albicans. Open Nanomedicine Journal 2017, 4 (1), 1–16. 10.2174/1875933501704010001.

[ref165] Sun L.; Liao K.; Li Y.; Zhao L.; Liang S.; Guo D.; Hu J.; Wang D. Synergy Between Polyvinylpyrrolidone-Coated Silver Nanoparticles and Azole Antifungal Against Drug-Resistant Candida albicans. J. Nanosci. Nanotechnol. 2016, 16 (3), 2325–2335. 10.1166/jnn.2016.10934. 27455637

[ref166] Li H.; Wang L.; Chai Y.; Cao Y.; Lu F. Synergistic effect between silver nanoparticles and antifungal agents on Candida albicans revealed by dynamic surface-enhanced Raman spectroscopy. Nanotoxicology 2018, 12 (10), 1230–1240. 10.1080/17435390.2018.1540729. 30501538

[ref167] Weitz I. S.; Maoz M.; Panitz D.; Eichler S.; Segal E. Combination of CuO nanoparticles and fluconazole: preparation, characterization, and antifungal activity against Candida albicans. J. Nanopart. Res. 2015, 17 (8), 342 10.1007/s11051-015-3149-4.

[ref168] abedzadeh hajar A.; dakhili m.; saghazadeh m.; aghaei S. S.; Nazari R. Synergistic Antifungal Effect of Fluconazole Combined with ZnO Nanoparticles against Candida albicans Strains from Vaginal Candidiasis. Medical Laboratory Journal 2020, 14 (3), 26–32. 10.29252/mlj.14.3.26.

[ref169] Jia D.; Sun W. Silver nanoparticles offer a synergistic effect with fluconazole against fluconazole-resistant Candida albicans by abrogating drug efflux pumps and increasing endogenous ROS. Infection, Genetics and Evolution 2021, 93, 104937 10.1016/j.meegid.2021.104937. 34029724

[ref170] Sharma N.; Jandaik S.; Kumar S. Synergistic activity of doped zinc oxide nanoparticles with antibiotics: ciprofloxacin, ampicillin, fluconazole and amphotericin B against pathogenic microorganisms. Anais da Academia Brasileira de Ciências 2016, 88 (3 suppl), 1689–1698. 10.1590/0001-3765201620150713. 27737336

[ref171] Hamad K. M.; Mahmoud N. N.; Al-Dabash S.; Al-Samad L. A.; Abdallah M.; Al-Bakri A. G. Fluconazole conjugated-gold nanorods as an antifungal nanomedicine with low cytotoxicity against human dermal fibroblasts. RSC Adv. 2020, 10 (43), 25889–25897. 10.1039/D0RA00297F. 35518580 PMC9055348

[ref172] Arias L. S.; Pessan J. P.; de Souza Neto F. N.; Lima B. H. R.; de Camargo E. R.; Ramage G.; Delbem A. C. B.; Monteiro D. R. Novel nanocarrier of miconazole based on chitosan-coated iron oxide nanoparticles as a nanotherapy to fight Candida biofilms. Colloids Surf., B 2020, 192, 111080 10.1016/j.colsurfb.2020.111080. 32361504

[ref173] Mussin J. E.; Roldán M. V.; Rojas F.; Sosa M. d. l. Á.; Pellegri N.; Giusiano G. Antifungal activity of silver nanoparticles in combination with ketoconazole against Malassezia furfur. AMB Express 2019, 9 (1), 131 10.1186/s13568-019-0857-7. 31432275 PMC6702292

[ref174] Ahmad A.; Wei Y.; Syed F.; Tahir K.; Taj R.; Khan A. U.; Hameed M. U.; Yuan Q. Amphotericin B-conjugated biogenic silver nanoparticles as an innovative strategy for fungal infections. Microbial Pathogenesis 2016, 99, 271–281. 10.1016/j.micpath.2016.08.031. 27591110

[ref175] Tutaj K.; Szlazak R.; Szalapata K.; Starzyk J.; Luchowski R.; Grudzinski W.; Osinska-Jaroszuk M.; Jarosz-Wilkolazka A.; Szuster-Ciesielska A.; Gruszecki W. I. Amphotericin B-silver hybrid nanoparticles: synthesis, properties and antifungal activity. Nanomedicine: Nanotechnology, Biology and Medicine 2016, 12 (4), 1095–1103. 10.1016/j.nano.2015.12.378. 26772425

[ref176] Harper A.; Vijayakumar V.; Ouwehand A. C.; ter Haar J.; Obis D.; Espadaler J.; Binda S.; Desiraju S.; Day R. Viral Infections, the Microbiome, and Probiotics. Frontiers in Cellular and Infection Microbiology 2021, 10, 1 10.3389/fcimb.2020.596166. PMC7907522 33643929

[ref177] Salata C.; Calistri A.; Alvisi G.; Celestino M.; Parolin C.; Palù G. Ebola Virus Entry: From Molecular Characterization to Drug Discovery. Viruses 2019, 11 (3), 274 10.3390/v11030274. 30893774 PMC6466262

[ref178] Coates M.; Blanchard S.; MacLeod A. S. Innate antimicrobial immunity in the skin: A protective barrier against bacteria, viruses, and fungi. PLOS Pathogens 2018, 14 (12), e1007353 10.1371/journal.ppat.1007353. 30522130 PMC6283644

[ref179] Kohil A.; Jemmieh S.; Smatti M. K.; Yassine H. M. Viral meningitis: an overview. Arch. Virol. 2021, 166 (2), 335–345. 10.1007/s00705-020-04891-1. 33392820 PMC7779091

[ref180] Renu K.; Prasanna P. L.; Valsala Gopalakrishnan A. Coronaviruses pathogenesis, comorbidities and multi-organ damage – A review. Life Sciences 2020, 255, 117839 10.1016/j.lfs.2020.117839. 32450165 PMC7243768

[ref181] Luo G.; Gao S. J. Global health concerns stirred by emerging viral infections. Journal of Medical Virology 2020, 92 (4), 399–400. 10.1002/jmv.25683. 31967329 PMC7166855

[ref182] Arthi V.; Parman J. Disease, downturns, and wellbeing: Economic history and the long-run impacts of COVID-19. Explorations in Economic History 2021, 79, 101381 10.1016/j.eeh.2020.101381. 33162564 PMC7606070

[ref183] Kausar S.; Said Khan F.; Ishaq Mujeeb Ur Rehman M.; Akram M.; Riaz M.; Rasool G.; Hamid Khan A.; Saleem I.; Shamim S.; Malik A. A review: Mechanism of action of antiviral drugs. International Journal of Immunopathology and Pharmacology 2021, 35, 205873842110026 10.1177/20587384211002621. PMC7975490 33726557

[ref184] Lampejo T. Influenza and antiviral resistance: an overview. European Journal of Clinical Microbiology & Infectious Diseases 2020, 39 (7), 1201–1208. 10.1007/s10096-020-03840-9. 32056049 PMC7223162

[ref185] Melville K.; Rodriguez T.; Dobrovolny H. M. Investigating Different Mechanisms of Action in Combination Therapy for Influenza. Frontiers in Pharmacology 2018, 9, 1 10.3389/fphar.2018.01207. 30405419 PMC6206389

[ref186] De Clercq E.; Li G. Approved Antiviral Drugs over the Past 50 Years. Clin. Microbiol. Rev. 2016, 29 (3), 695–747. 10.1128/CMR.00102-15. 27281742 PMC4978613

[ref187] Tortella G. R.; Rubilar O.; Diez M. C.; Padrão J.; Zille A.; Pieretti J. C.; Seabra A. B. Advanced Material Against Human (Including Covid-19) and Plant Viruses: Nanoparticles As a Feasible Strategy. Global Challenges 2021, 5 (3), 2000049 10.1002/gch2.202000049. 33614127 PMC7883180

[ref188] Tompa D. R.; Immanuel A.; Srikanth S.; Kadhirvel S. Trends and strategies to combat viral infections: A review on FDA approved antiviral drugs. Int. J. Biol. Macromol. 2021, 172, 524–541. 10.1016/j.ijbiomac.2021.01.076. 33454328 PMC8055758

[ref189] Sportelli M. C.; Izzi M.; Kukushkina E. A.; Hossain S. I.; Picca R. A.; Ditaranto N.; Cioffi N. Can Nanotechnology and Materials Science Help the Fight against SARS-CoV-2?. Nanomaterials 2020, 10 (4), 802 10.3390/nano10040802. 32326343 PMC7221591

[ref190] Delshadi R.; Bahrami A.; McClements D. J.; Moore M. D.; Williams L. Development of nanoparticle-delivery systems for antiviral agents: A review. J. Controlled Release 2021, 331, 30–44. 10.1016/j.jconrel.2021.01.017. PMC7803629 33450319

[ref191] Chakravarty M.; Vora A. Nanotechnology-based antiviral therapeutics. Drug Delivery and Translational Research 2021, 11 (3), 748–787. 10.1007/s13346-020-00818-0. 32748035 PMC7398286

[ref192] Sokolova V.; Westendorf A. M.; Buer J.; Überla K.; Epple M. The potential of nanoparticles for the immunization against viral infections. J. Mater. Chem. B 2015, 3 (24), 4767–4779. 10.1039/C5TB00618J. 32262665

[ref193] Lin Z.; Li Y.; Guo M.; Xu T.; Wang C.; Zhao M.; Wang H.; Chen T.; Zhu B. The inhibition of H1N1 influenza virus-induced apoptosis by silver nanoparticles functionalized with zanamivir. RSC Adv. 2017, 7 (2), 742–750. 10.1039/C6RA25010F.

[ref194] Li Y.; Lin Z.; Zhao M.; Guo M.; Xu T.; Wang C.; Xia H.; Zhu B. Reversal of H1N1 influenza virus-induced apoptosis by silver nanoparticles functionalized with amantadine. RSC Adv. 2016, 6 (92), 89679–89686. 10.1039/C6RA18493F.

[ref195] Li Y.; Lin Z.; Zhao M.; Xu T.; Wang C.; Hua L.; Wang H.; Xia H.; Zhu B. Silver Nanoparticle Based Codelivery of Oseltamivir to Inhibit the Activity of the H1N1 Influenza Virus through ROS-Mediated Signaling Pathways. ACS Appl. Mater. Interfaces 2016, 8 (37), 24385–24393. 10.1021/acsami.6b06613. 27588566

[ref196] Stanley M.; Cattle N.; McCauley J.; Martin S. R.; Rashid A.; Field R. A.; Carbain B.; Streicher H. ‘TamiGold’: phospha-oseltamivir-stabilised gold nanoparticles as the basis for influenza therapeutics and diagnostics targeting the neuraminidase (instead of the hemagglutinin). MedChemComm 2012, 3 (11), 1373 10.1039/c2md20034a.

[ref197] Chen L.; Liang J. An overview of functional nanoparticles as novel emerging antiviral therapeutic agents. Materials Science and Engineering: C 2020, 112, 110924 10.1016/j.msec.2020.110924. 32409074 PMC7195146

[ref198] Slavin Y. N.; Asnis J.; Häfeli U. O.; Bach H. Metal nanoparticles: understanding the mechanisms behind antibacterial activity. J. Nanobiotechnol. 2017, 15 (1), 65 10.1186/s12951-017-0308-z. PMC5627441 28974225

[ref199] Gold K.; Slay B.; Knackstedt M.; Gaharwar A. K. Antimicrobial Activity of Metal and Metal-Oxide Based Nanoparticles. Advanced Therapeutics 2018, 1 (3), 1700033 10.1002/adtp.201700033.

[ref200] Zhou J.; Hu Z.; Zabihi F.; Chen Z.; Zhu M. Progress and Perspective of Antiviral Protective Material. Advanced Fiber Materials 2020, 2 (3), 123–139. 10.1007/s42765-020-00047-7. PMC7309218 38624352

[ref201] Niño-Martínez N.; Salas Orozco M. F.; Martínez-Castañón G.-A.; Torres Méndez F.; Ruiz F. Molecular Mechanisms of Bacterial Resistance to Metal and Metal Oxide Nanoparticles. International Journal of Molecular Sciences 2019, 20 (11), 2808 10.3390/ijms20112808. 31181755 PMC6600416

[ref202] Kapoor G.; Saigal S.; Elongavan A. Action and resistance mechanisms of antibiotics: A guide for clinicians. Journal of Anaesthesiology Clinical Pharmacology 2017, 33 (3), 300 10.4103/joacp.JOACP_349_15. 29109626 PMC5672523

[ref203] Lingzhi L.; Haojie G.; Dan G.; Hongmei M.; Yang L.; Mengdie J.; Chengkun Z.; Xiaohui Z. The role of two-component regulatory system in β-lactam antibiotics resistance. Microbiological Research 2018, 215, 126–129. 10.1016/j.micres.2018.07.005. 30172298

[ref204] Retsema J.; Fu W. Macrolides: structures and microbial targets. Int. J. Antimicrob. Agents 2001, 18, 3–10. 10.1016/S0924-8579(01)00401-0. 11574188

[ref205] Vázquez-Laslop N.; Mankin A. S. How Macrolide Antibiotics Work. Trends Biochem. Sci. 2018, 43 (9), 668–684. 10.1016/j.tibs.2018.06.011. 30054232 PMC6108949

[ref206] Grossman T. H. Tetracycline Antibiotics and Resistance. Cold Spring Harbor Perspectives in Medicine 2016, 6 (4), a025387 10.1101/cshperspect.a025387. 26989065 PMC4817740

[ref207] Fàbrega A.; Madurga S.; Giralt E.; Vila J. Mechanism of action of and resistance to quinolones. Microbial Biotechnology 2009, 2 (1), 40–61. 10.1111/j.1751-7915.2008.00063.x. 21261881 PMC3815421

[ref208] Kim D.-W.; Thawng C. N.; Lee K.; Wellington E. M. H.; Cha C.-J. A novel sulfonamide resistance mechanism by two-component flavin-dependent monooxygenase system in sulfonamide-degrading actinobacteria. Environ. Int. 2019, 127, 206–215. 10.1016/j.envint.2019.03.046. 30928844

[ref209] Khondker A.; Rheinstädter M. C. How do bacterial membranes resist polymyxin antibiotics?. Communications Biology 2020, 3 (1), 77 10.1038/s42003-020-0803-x. 32066819 PMC7026071

[ref210] Doi Y.; Wachino J.-i.; Arakawa Y. Aminoglycoside Resistance. Infectious Disease Clinics of North America 2016, 30 (2), 523–537. 10.1016/j.idc.2016.02.011. 27208771 PMC4878400

[ref211] Zeng D.; Debabov D.; Hartsell T. L.; Cano R. J.; Adams S.; Schuyler J. A.; McMillan R.; Pace J. L. Approved Glycopeptide Antibacterial Drugs: Mechanism of Action and Resistance. Cold Spring Harbor Perspectives in Medicine 2016, 6 (12), a026989 10.1101/cshperspect.a026989. 27663982 PMC5131748

[ref212] Miller M. B.; Gilligan P. H. Mechanisms and Detection of Antimicrobial Resistance. Principles and Practice of Pediatric Infectious Diseases 2012, 1421–1433.e7. 10.1016/B978-1-4377-2702-9.00292-0.

[ref213] Michalopoulos A. S.; Livaditis I. G.; Gougoutas V. The revival of fosfomycin. International Journal of Infectious Diseases 2011, 15 (11), e732–e739. 10.1016/j.ijid.2011.07.007. 21945848

[ref214] Geilich B. M.; van de Ven A. L.; Singleton G. L.; Sepúlveda L. J.; Sridhar S.; Webster T. J. Silver nanoparticle-embedded polymersome nanocarriers for the treatment of antibiotic-resistant infections. Nanoscale 2015, 7 (8), 3511–3519. 10.1039/C4NR05823B. 25628231

[ref215] Kontoyiannis D. P. Antifungal Resistance: An Emerging Reality and A Global Challenge. Journal of Infectious Diseases 2017, 216 (suppl_3), S431–S435. 10.1093/infdis/jix179. 28911044

[ref216] Fuentefria A. M.; Pippi B.; Dalla Lana D. F.; Donato K. K.; de Andrade S. F. Antifungals discovery: an insight into new strategies to combat antifungal resistance. Letters in Applied Microbiology 2018, 66 (1), 2–13. 10.1111/lam.12820. 29112282

[ref217] Ryu W.-S. Virus Life Cycle. Molecular Virology of Human Pathogenic Viruses 2017, 31–45. 10.1016/B978-0-12-800838-6.00003-5.

[ref218] Kaufmann S. H. E.; Dorhoi A.; Hotchkiss R. S.; Bartenschlager R. Host-directed therapies for bacterial and viral infections. Nat. Rev. Drug Discovery 2018, 17 (1), 35–56. 10.1038/nrd.2017.162. 28935918 PMC7097079

[ref219] Adamson C. S.; Chibale K.; Goss R. J. M.; Jaspars M.; Newman D. J.; Dorrington R. A. Antiviral drug discovery: preparing for the next pandemic. Chem. Soc. Rev. 2021, 50 (6), 3647–3655. 10.1039/D0CS01118E. 33524090

[ref220] Kumar N.; Sharma S.; Kumar R.; Tripathi B. N.; Barua S.; Ly H.; Rouse B. T. Host-Directed Antiviral Therapy. Clin. Microbiol. Rev. 2020, 33 (3), 1 10.1128/CMR.00168-19. PMC7227448 32404434

[ref221] Makvandi P.; Wang C. y.; Zare E. N.; Borzacchiello A.; Niu L. n.; Tay F. R. Metal-Based Nanomaterials in Biomedical Applications: Antimicrobial Activity and Cytotoxicity Aspects. Adv. Funct. Mater. 2020, 30 (22), 1910021 10.1002/adfm.201910021.

[ref222] Sukhanova A.; Bozrova S.; Sokolov P.; Berestovoy M.; Karaulov A.; Nabiev I. Dependence of Nanoparticle Toxicity on Their Physical and Chemical Properties. Nanoscale Res. Lett. 2018, 13 (1), 44 10.1186/s11671-018-2457-x. 29417375 PMC5803171

[ref223] Rehman K.; Kamran S. H.; Hamid Akash M. S. Toxicity of antibiotics. Antibiotics and Antimicrobial Resistance Genes in the Environment 2020, 234–252. 10.1016/B978-0-12-818882-8.00016-4.

[ref224] Tverdek F. P.; Kofteridis D.; Kontoyiannis D. P. Antifungal agents and liver toxicity: a complex interaction. Expert Review of Anti-infective Therapy 2016, 14 (8), 765–776. 10.1080/14787210.2016.1199272. 27275514

[ref225] Rauseo A. M; Coler-Reilly A.; Larson L.; Spec A. Hope on the Horizon: Novel Fungal Treatments in Development. Open Forum Infectious Diseases 2020, 7 (2), ofaa016 10.1093/ofid/ofaa016. 32099843 PMC7031074

[ref226] Niemirowicz K.; Durnaś B.; Piktel E.; Bucki R. Development of antifungal therapies using nanomaterials. Nanomedicine 2017, 12 (15), 1891–1905. 10.2217/nnm-2017-0052. 28703684

[ref227] Kayaaslan B.; Guner R. Adverse effects of oral antiviral therapy in chronic hepatitis B. World Journal of Hepatology 2017, 9 (5), 227 10.4254/wjh.v9.i5.227. 28261380 PMC5316843

